# Nanostructured Cathode Materials for Rechargeable Lithium-Ion Batteries: Synthesis, Morphology, and Performances

**DOI:** 10.3390/ijms27156797

**Published:** 2026-07-29

**Authors:** Rasha S. El-Tawil, Ashraf E. Abdel-Ghany, Ahmed M. Hashem, Alain Mauger, Christian M. Julien

**Affiliations:** 1 Inorganic Chemistry Department, National Research Center, 33 El Bohouth Street, Dokki, Giza 12622, Egypt; r2samir@yahoo.com (R.S.E.-T.); achraf_28@yahoo.com (A.E.A.-G.); ahmedh242@yahoo.com (A.M.H.); 2 Institut de Minéralogie, de Physique des Matériaux et Cosmologie (IMPMC), Sorbonne Université, UMR-CNRS 7590, 4 Place Jussieu, 75752 Paris, France; alain.mauger@sorbonne-universite.fr

**Keywords:** lithium-ion batteries, cathode materials, layered structure, spinel LiMn_2_O_4_, polyanionic phosphates, lithium and nickel-rich oxides, synthesis processes

## Abstract

High-performance energy sources for electric vehicles and portable electronic devices require state-of-the-art lithium-ion batteries (LIBs) characterized by high energy density, superior power output, and excellent long-term cycling stability. Among the various components of LIBs, the cathode material plays a decisive role in determining the electrochemical performance, safety, and commercial viability of the battery. To meet the growing demands of modern applications, these materials must combine high capacity with structural stability, thermal safety, cost-effectiveness, and excellent rate capability. This article reviews the most widely used cathode materials with layered, spinel, and olivine structures. Their key advantages and intrinsic limitations are analyzed in detail, alongside strategies to enhance performance through elemental doping and surface coating approaches. Furthermore, the review presents simple, cost-effective, and industrially scalable synthesis methods, and highlights advanced characterization techniques that provide a deeper understanding of their nanostructured features and electrochemical behavior.

## 1. Introduction

The persistent reliance on fossil fuels—particularly oil, coal, and natural gas—has led to severe environmental pollution and an escalating global energy crisis. Consequently, considerable research effort has been devoted to developing sustainable and renewable energy resources, such as solar, wind, and tidal energy. However, the intermittent and fluctuating nature of these renewable sources necessitates the advancement of efficient energy storage materials and devices to ensure a stable and continuous power supply to the grid.

**Figure 1:**
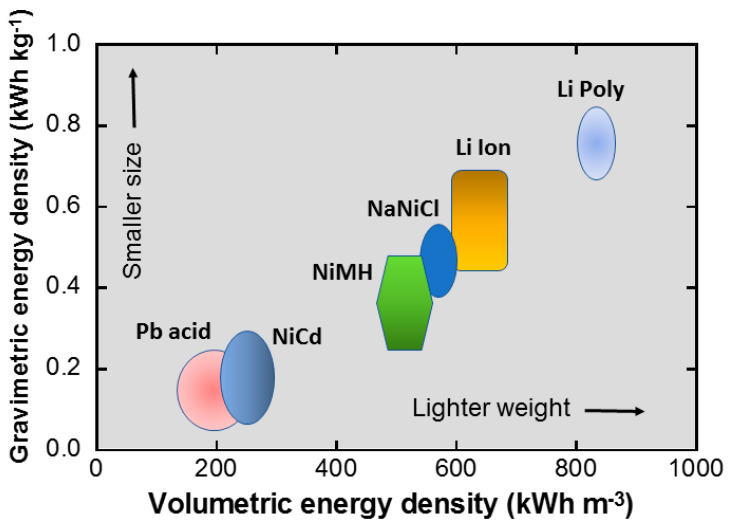
Comparison of energy density of lithium-ion batteries with other types of batteries.

Lithium-ion batteries (LIBs) have established themselves as the dominant rechargeable energy storage technology since their commercialization by Sony in 1991 [1]. Their widespread adoption in portable consumer electronics and electric vehicles stems from a unique combination of desirable properties, including high energy density, long cycle life, high efficiency, lightweight construction, environmental compatibility, and relatively low maintenance requirements [2]. Unlike primary cells, which are designed for single-use applications, LIBs are secondary batteries that rely on reversible electrochemical reactions, enabling repeated charge–discharge cycles and prolonged operational lifespan. The conceptual foundations of rechargeable lithium batteries date back to the early 1970s, when Whittingham introduced TiS_2_ as a cathode material paired with lithium metal for the anode [3,4]. This pioneering work demonstrated the feasibility of lithium intercalation chemistry and marked the beginning of modern research into rechargeable lithium batteries. Shortly thereafter, Besenhard proposed using graphite as a host anode material in combination with transition-metal oxide cathodes, thereby contributing to the development of safer and more stable electrochemical systems [5]. A major breakthrough occurred in 1979 when Goodenough and Mizushima successfully employed layered LiCoO_2_ as a cathode material, thereby laying the technological foundation for modern LIBs [6]. These pioneering contributions collectively accelerated the advancement and commercialization of LIB technology. As illustrated in Figure 1 and summarized in Table 1, comparative analyses demonstrate that LIBs outperform conventional secondary batteries in terms of energy density, power capability, energy efficiency, and low self-discharge rates. These superior electrochemical characteristics have firmly established LIBs as the dominant rechargeable power source for next-generation energy storage systems [7]. Furthermore, their high energy density, compact size, and lightweight configuration make LIBs particularly attractive for advanced applications, including electric vehicles (EVs) and hybrid electric vehicles (HEVs), and large-scale renewable energy storage technologies.

This review presents an in-depth overview of recent advances in cathode materials for Li-ion batteries, especially focusing on lithiated oxides with rock-salt, spinel, and olivine structures, and their derivatives. Their key advantages and intrinsic limitations are discussed in detail, alongside strategies to enhance performance through elemental doping and surface coating approaches. In addition, facile, scalable, and cost-effective synthesis methods for these cathode materials and advanced characterization techniques employed to achieve a deep understanding of their nanostructured features and electrochemical behavior are discussed. However, a comprehensive cross-family comparison that systematically links these modification strategies to advanced characterization tools and incorporates the most recent industrial and recycling trends remains notably absent in the existing literature.

To address this gap, unlike previous articles that generally focus on a single cathode family (e.g., only Ni-rich layered oxides, or only olivine phosphates) or on a single modification level (doping, or coating, or synthesis), the present review provides an integrated, cross-family comparison spanning layered oxides, spinel oxides, and polyanionic (olivine and non-olivine) cathodes, explicitly linking particle-scale nanostructuring, surface/interface engineering, and bulk doping to the characterization techniques used to verify them. In addition, this review consolidates the three generations of intraparticle compositional engineering (discrete core–shell, concentration gradient, and full-concentration-gradient architectures) within a single comparative framework, and incorporates the most recent (2024–2026) developments in single-crystal Ni-rich cathodes, gradient/dual-doping strategies, solid-state-electrolyte-compatible interfaces, and cathode upcycling/recycling, which are largely absent from earlier reviews on this topic.

## 2. Components and Function of LIBs

**Figure 2:**
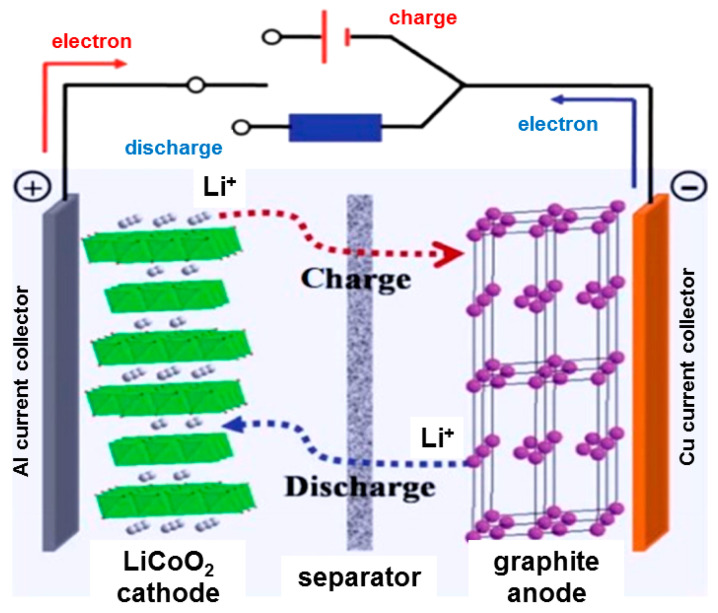
Schematic diagram of a lithium-ion battery. Reproduced from [8]. Copyright 2020 under the terms of the Creative Commons Attribution Non-Commercial License.

Like other rechargeable battery systems, LIBs are secondary electrochemical cells that convert the chemical energy stored in their active materials into electrical energy. A typical LIB cell consists of a positive electrode (cathode) and a negative electrode (anode), in between a porous separator impregnated with an electrolyte solution [8]. In conventional LIBs, both electrodes act as host materials capable of reversibly inserting and extracting lithium ions during charging and discharging processes (Figure 2). Graphitic carbon is commonly used as the anode material, while lithium transition-metal oxides, such as Li*M*O_2_ (where *M* = Co, Mn, or Ni), are widely employed as cathode materials. The separator plays a crucial role in battery safety and performance by preventing direct physical contact between the electrodes—thereby avoiding short circuits—while allowing the transport of lithium ions through the electrolyte. The electrolyte, typically composed of a lithium salt dissolved in an organic solvent, serves as an ionic conductor facilitating ion migration between the electrodes, while remaining an electronic insulator [9].

During the charging process of an LIB, Li^+^ ions migrate from the cathode, which progressively becomes a lithium-deficient Li_1−x_*M*O_2_ compound, to the anode, where lithium is stored, thereby forming a lithium-rich phase. This migration occurs through the electrolyte under the influence of an external energy source and is accompanied by the oxidation (delithiation) of the cathode material. During discharge, the reverse process takes place: lithium ions return to the cathode via lithiation (reduction), thereby restoring the initial chemical composition of the electrode materials. The specific capacity of an LIB is primarily determined by the amount of lithium that can be reversibly extracted and reinserted into the cathode structure, as well as by the kinetics of these processes. Consequently, the battery’s energy capacity and operating voltage depend heavily on the structural, electronic, and chemical properties of the cathode material. This highlights the pivotal role of the cathode in determining the overall electrochemical performance of LIBs. Beyond its electrochemical importance, the cathode is also one of the most expensive components of an LIB, often accounting for more than half of the total manufacturing cost. For this reason, significant research efforts have been devoted to developing new cathode materials with superior electrochemical properties, and to optimizing existing compounds [10].

## 3. Cathode Materials for Rechargeable LIBs

**Figure 3:**
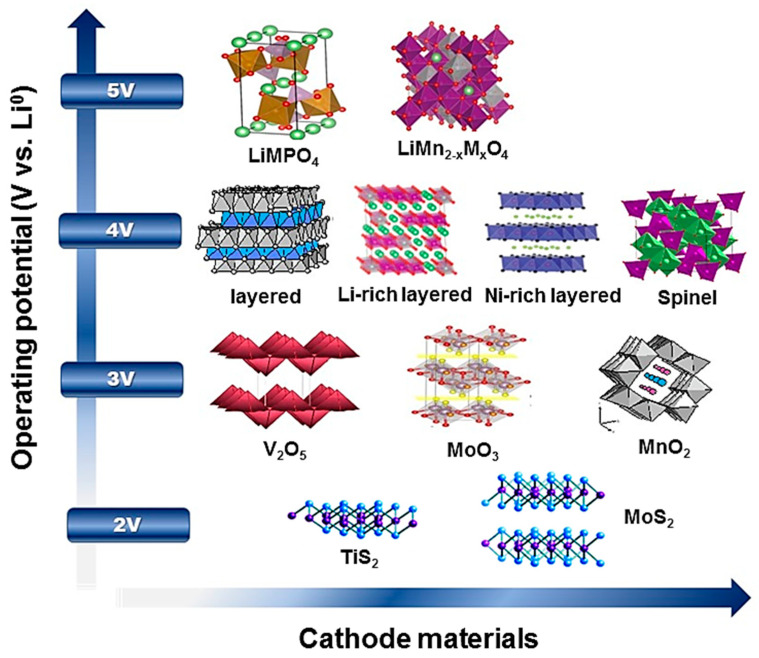
Scheme for different cathode materials based on operating potential vs. lithium.

Based on their average operating voltage relative to metallic lithium, cathode materials can generally be classified into four main categories, as illustrated in Figure 3.

(I) Low-voltage cathode materials (∼2 V): This category includes transition-metal dichalcogenides with a two-dimensional (2D) structure, such as titanium disulfide (TiS_2_) and molybdenum disulfide (MoS_2_). These materials are initially lithium-free and therefore require lithium insertion during the first discharge cycle to become electrochemically active.
(II) Intermediate-voltage cathode materials (∼3 V): This group includes manganese dioxide (MnO_2_), molybdenum trioxide (MoO_3_), and lithium iron phosphate (LiFePO_4_), as well as vanadium-based oxides such as V_2_O_5_ and LiV_3_O_8_. As with the low-voltage category, several of these compounds are initially unlithiated and undergo lithiation during the first discharge. Among them, LiFePO_4_ has attracted significant interest due to its excellent thermal stability, long cycle life, and environmental compatibility, despite a moderate operating voltage and low electrical conductivity.
(III) High-voltage cathode materials (∼4 V): This category includes the most commercially successful cathode material, lithium cobalt oxide (LiCoO_2_), as well as other layered oxides such as lithium nickel oxide (LiNiO_2_), lithium nickel manganese cobalt oxide (LiNi_1/3_Mn_1/3_Co_1/3_O_2_), and nickel-rich compositions like NMC811 (LiNi_0.8_Co_0.1_Mn_0.1_O_2_). Furthermore, materials with a three-dimensional (3D) spinel structure—notably lithium manganese oxide (LiMn_2_O_4_)—also belong to this group. These cathode materials are already lithiated and are highly valued for their high operating voltage, high energy density, and good cycling stability, making them particularly well suited for commercial LIB applications.
(IV) Ultra-high-voltage cathode materials (∼5 V): This category comprises advanced cathode materials capable of operating at very high potentials, notably olivine-structured phosphates such as lithium manganese phosphate (LiMnPO_4_) and lithium cobalt phosphate (LiCoPO_4_), as well as spinel-type compounds with the formula Li*M*_x_Mn_4−x_O_8_ (where *M* = Fe, Co). Thanks to their robust three-dimensional frameworks, these materials exhibit enhanced structural stability during lithium insertion and extraction processes. Although still under active development, ultra-high-voltage cathodes are considered promising candidates for next-generation LIBs due to their potential to significantly increase energy density and operating voltage.

Overall, these cathode materials—encompassing one-dimensional (1D), two-dimensional (2D), and three-dimensional (3D) structural frameworks—have been extensively studied and widely implemented in commercial LIB technologies. The diversity of their structures and electrochemical properties offers a wide range of performance characteristics, enabling the development of batteries tailored to specific applications, from portable electronics to electric vehicles and large-scale energy storage systems. Continued progress in the design and optimization of cathode materials remains essential for enhancing the performance of next-generation LIBs.

To achieve high electrochemical performance and practical viability, cathode materials must satisfy several key requirements:

- *Large lithium chemical potential difference*: A substantial potential difference between the cathode and anode is necessary to obtain a high cell voltage and, consequently, a high energy output.
- *High lithium-ion storage capability*: The cathode should be capable of reversibly accommodating a large quantity of lithium ions per formula unit in order to maximize the specific capacity of the battery.
- *Structural stability during cycling*: The crystal structure of the cathode material must maintain sufficient integrity during repeated lithium intercalation and deintercalation reactions to ensure a long cycle life and reliable rechargeability. Although lithium intercalation and deintercalation reactions inevitably induce some degree of lattice strain, volume change, and localized structural rearrangement, these changes must remain largely reversible and not lead to progressive structural degradation during long-term cycling.
- *Efficient electronic and ionic transport*: High electronic conductivity and rapid lithium-ion diffusion are essential for improving charge–discharge kinetics and reducing internal resistance.
- *Electrochemical and thermal stability*: Cathode materials must remain chemically and structurally stable within the operating voltage and temperature ranges, particularly in contact with the electrolyte, in order to minimize side reactions and material degradation.
- *Low Cost and environmental compatibility*: For large-scale commercialization and sustainable deployment, cathode materials should be economically feasible, abundant, and environmentally benign.
- *Highly reversible Li^+^ intercalation/deintercalation*: Lithium insertion and extraction must occur reversibly at a sufficiently high redox potential to ensure both high energy efficiency and prolonged cycling stability.

**Figure 4:**
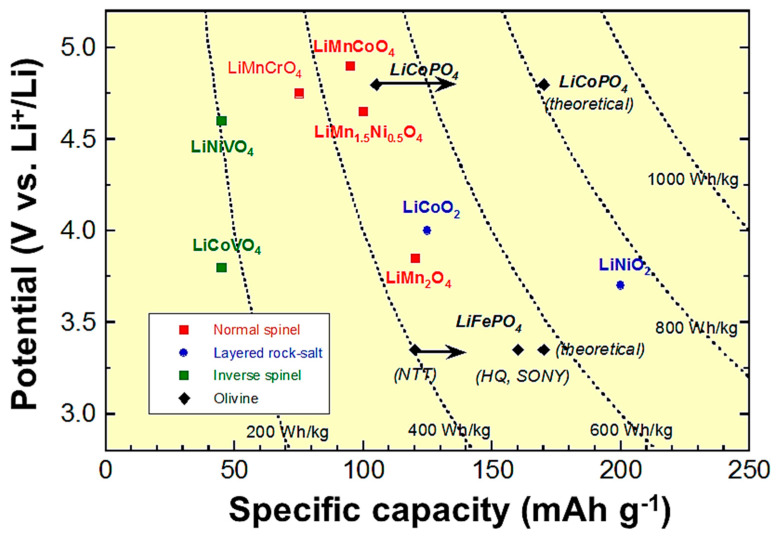
Potential vs. specific capacity for various cathode materials used in LIBs. Reproduced from [12]. Copyright 2016 Springer.

The continued development and widespread adoption of LIBs therefore depend heavily on advances in cathode materials that simultaneously deliver high energy density, enhanced safety, and economic viability [11]. Figure 4 presents the relationship between cell voltage and specific capacity for various cathode materials, thereby providing an overview of their respective electrochemical performance domains [12].

### 3.1. Layered LiMO_2_ Transition-Metal Oxides (4 V)

Layered transition-metal oxides with the general formula Li*M*O_2_ (where *M* = Co, Mn, Ni) constitute the most widely used class of cathode materials in commercial rechargeable LIBs. Their two-dimensional layered crystal structure enables reversible intercalation and deintercalation of lithium ions with high efficiency, thereby facilitating high energy density and excellent electrochemical performance. Due to their relatively high operating voltage (~4 V), favorable cycling behavior, and proven synthesis processes, these materials have become central to the development of high-performance LIB technologies.

#### 3.1.1. Lithium Cobalt Oxide (LiCoO_2_, LCO)

**Figure 5:**
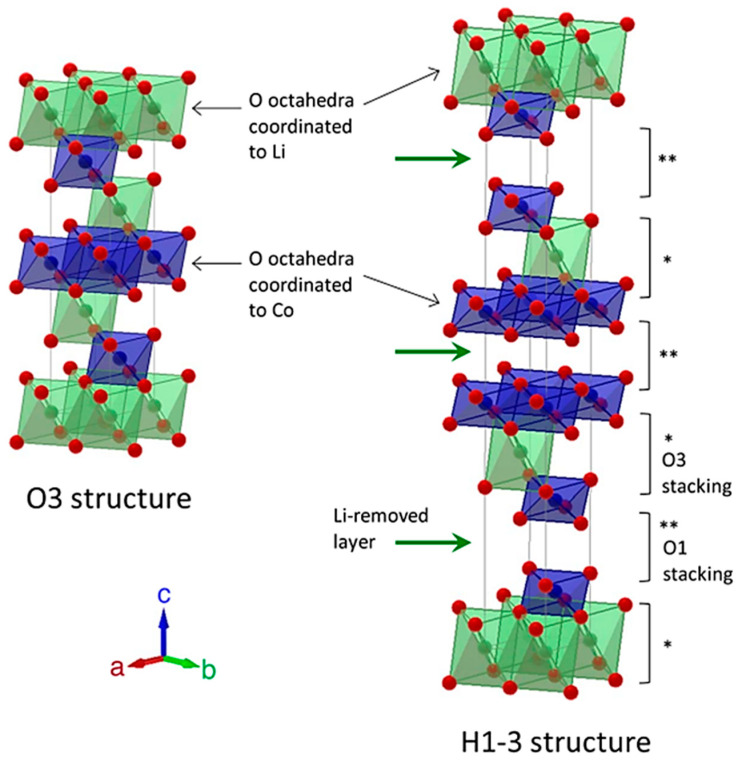
Representation of the crystal structure of layered rock-salt LiCoO_2_ associated with Li deinsertion during charging. Li_x_CoO_2_ transitions from O3 (octahedral triple-phase) to H1–3 (hybrid-phase of octahedral single-phase O1 and O3) at a potential of ~4.5 V (x~0.3). * indicates the O2 stacking and ** the O1 stacking. Reproduced from [15]. Copyright 2021 under the terms of the Creative Commons Attribution 4.0 License (CC-BY).

Lithium cobalt oxide (LCO) was first proposed as a reversible lithium insertion material by John Goodenough’s team in 1980 [6], and subsequently used by Akira Yoshino in the prototypal rechargeable lithium-ion battery unveiled in 1986 [13]. Since then, LCO has remained one of the most widely investigated and commercially utilized cathode materials in LIB technology, owing to its well-defined layered structure and reliable electrochemical performance. LCO is typically synthesized at temperatures around 750 °C, forming a layered trigonal structure (often referred to as hexagonal) belonging to the *R*$\bar{3}$*m* space group [14]. In this structure, Li^+^ and Co^3+^ ions occupy the octahedral 3*a* and 3*b* sites, respectively, and are separated by layers of oxygen ions arranged in a cubic close-packed configuration. As illustrated in Figure 5, the unit cell of O3-type LCO consists of three slabs of edge-sharing CoO_6_ octahedra alternating with lithium layers, thereby creating a stable layered framework [15]. During discharge (lithiation), LCO retains its hexagonal structure. During charging, lithium ions are extracted from the lattice to form Li_1−x_CoO_2_-type compounds, accompanied by the oxidation of Co^3+^ ions to maintain electrical neutrality. However, when more than approximately 50% of the lithium is removed, a structural phase transition occurs, shifting the material from the hexagonal to a monoclinic structure; this results in reduced structural stability and diminished electrochemical reversibility. This intrinsic structural instability, combined with the high cost and limited thermal safety of cobalt-based materials, represents one of the main limitations of LCO cathodes. Consequently, the practical specific capacity of LCO is generally limited to approximately 140 mAh g^−1^—nearly half of its theoretical capacity (280 mAh g^−1^)—because further delithiation accelerates structural degradation and compromises cycling stability.

**Figure 6:**
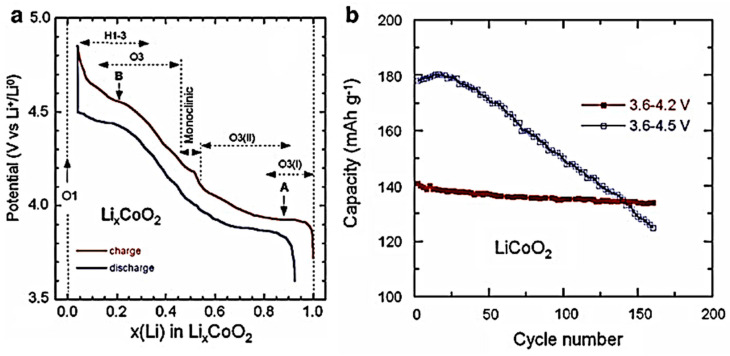
Electrochemical characteristics of the LiCoO_2_ cathode material. (**a**) Charge/discharge profile of a Li//LiCoO_2_ half-cell cycled in the potential range 3.0–4.8 V. Powders were synthesized by the sol–gel method. (**b**) Capacity retention as a function of the working region. Reproduced from [12]. Copyright 2016 Springer Nature.

Despite its widespread commercial use, LCO exhibits several critical limitations, including structural instability at high states of charge, high cost associated with cobalt content, and significant safety concerns under abusive operating conditions. These drawbacks have motivated extensive research aimed at developing alternative cathode materials and devising modification strategies to improve the electrochemical performance and stability of LCO. Among the various approaches investigated, elemental doping has proven particularly effective. Substitutional doping with main-group elements (such as Al, Mg, and B) or transition metals (such as Ti, Mn, Cr, and Fe) has been shown to reinforce structural stability, inhibit detrimental phase transitions, and improve capacity retention during cycling. Concurrently, surface coating with metal oxides effectively reduces electrolyte decomposition and minimizes interfacial resistance, thereby stabilizing LCO at high operating voltages. By combining these strategies, optimized LCO-based cathodes have demonstrated discharge capacities exceeding 200 mAh g^−1^ under carefully controlled conditions [16,17,18,19]. This enhancement is primarily attributed to the stabilization of the crystal lattice and the inhibition of harmful phase transitions during cycling. Figure 6 illustrates the electrochemical behavior of a nanostructured LiCoO_2_ electrode synthesized via the sol–gel method. Within the 2.5−4.3 V voltage window, the charge/discharge profiles exhibit the characteristic plateau at approximately 3.92 V, corresponding to the initial H1 → H2 phase transition associated with a semiconductor-to-metal transition (Figure 6a). For cells cycled between 3.6 and 4.2 V, the reversible capacity remains relatively stable over repeated cycles. In contrast, a marked capacity fading is observed when the upper cut-off voltage is extended to 4.5 V (3.6−4.5 V range), primarily due to oxygen loss and structural degradation induced by extensive lithium extraction (Figure 6b).

*Critical assessment.* LiCoO_2_ still remains one of the most widely studied and commercially utilized cathode materials, owing to its well-established layered structure and stable electrochemical performance within a moderate operating voltage window (≈140 mAh g^−1^ practical vs. a theoretical 274 mAh g^−1^; see Section 3.1.1). However, its practical energy density remains fundamentally limited by the irreversible hexagonal-to-monoclinic transition and oxygen release that accompany extensive delithiation beyond approximately 4.2–4.3 V. Although elemental doping and surface coating strategies have enabled practical capacities exceeding 200 mAh g^−1^ [16,17,18,19], these improvements depend heavily on the chemical nature of the dopant, coating uniformity, and synthesis conditions. Furthermore, they are typically demonstrated in half-cell configurations with low electrode loading rather than under realistic full-cell conditions, making direct quantitative comparisons between different studies difficult.

#### 3.1.2. Lithium Nickel Oxide (LNO)

Lithium nickel oxide (LiNiO_2_, LNO) was first introduced as a cathode material by Dyer et al. [20]. By replacing Co^3+^ with Ni^3+^, while preserving the same layered crystal framework as LiCoO_2_, LNO was initially viewed as a lower-cost and potentially safer alternative to LCO, as illustrated in Figure 5. LNO exhibits an average operating voltage of approximately 4 V vs. Li^+^/Li, a high theoretical specific capacity close to 250 mAh g^−1^, and an electrode density of about 3.3 g cm^−3^, making it an attractive candidate for high-energy-density LIBs [21,22]. Despite these promising characteristics, the practical application of LNO has been significantly limited by several intrinsic challenges. A major difficulty lies in synthesizing LNO with the ideal *R*$\bar{3}$*m* layered structure. During synthesis, nickel ions tend to migrate into lithium sites because the ionic radii of Ni^2+^ and Li^+^ are relatively similar. This cation mixing disrupts the layered ordering, hinders lithium-ion diffusion, and deteriorates electrochemical performance [23]. Furthermore, LNO suffers from structural instability at high states of delithiation, leading to phase transitions, surface degradation, and poor cycling stability [24]. Thermal instability and high reactivity with the electrolyte at high voltages also raise safety concerns, thereby restricting its widespread commercial implementation. To overcome these limitations, numerous strategies have been investigated, including surface coating and partial substitution with other metal ions such as manganese (Mn), cobalt (Co), and aluminum (Al). These modifications aim to reduce cation disorder, reinforce structural stability, and enhance electrochemical performance. More recently, Huang et al. examined the effect of introducing a small amount (1 mol%) of niobium (Nb^5+^) into LNO. Due to its high valence state, relatively large ionic radius (0.64 Å), and strong Nb–O bonding, Nb doping effectively reduced Ni^2+^/Li^+^ cation mixing and improved the structural stability of the layered framework. The modified compound, LiNi_0.99_Nb_0.01_O_2_, demonstrated enhanced electrochemical performance, notably a capacity retention of 91.4% after 100 cycles at 0.5 C, and excellent rate capability (143 mAh g^−1^ at 5 C). In comparison, unmodified LNO exhibited only 69.2% capacity retention and a discharge capacity of 127 mAh g^−1^ under identical conditions. These results highlight the effectiveness of targeted doping strategies in mitigating the intrinsic drawbacks of LNO and improving its suitability for high-performance LIB applications [25].

**Figure 7:**
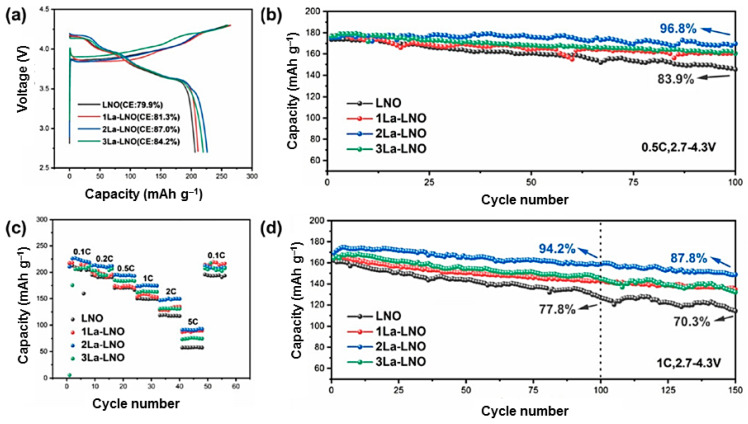

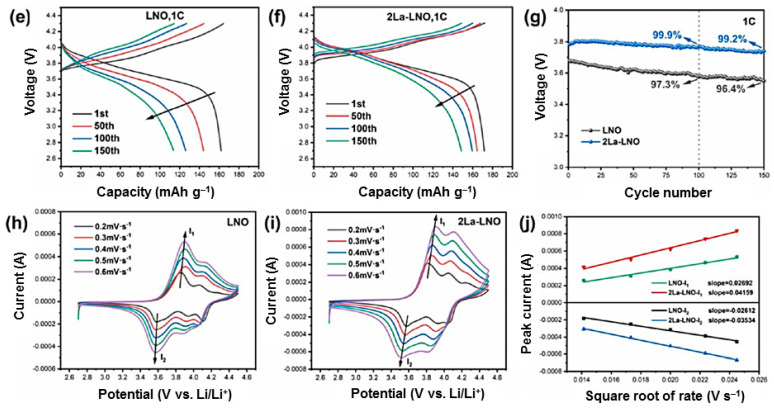
Electrochemical behavior of 1–3 wt.% La-doped LiNiO_2_ cathodes. (**a**) Initial charge and discharge curve of all samples at 0.1 C; (**b**) cycle performance curve under 2.7–4.3 V at 0.5 C; (**c**) rate performance; (**d**) cycle performance curve under 2.7–4.3 V at 1 C; (**e**,**f**) charge and discharge curves of pristine LNO and 2 wt.% La-LNO for different cycles in (**d**); (**g**) middle discharge voltage of pristine LNO and 2 wt.% La-LNO in (**d**); (**h**,**i**) CV curves at different sweep speeds of LNO and 2 wt.% La-LNO; (**j**) linear relationship between the anodic/cathodic peak current (*I*_p_) and the square root of the scan rate (ν^1/2^) in (**h**,**i**). Reproduced from [26]. Copyright 2025 American Chemical Society.

In another study, Wei et al. proposed a lanthanum-doping strategy aimed at stabilizing lattice oxygen in LNO cathodes to achieve highly durable LIBs. Incorporating La at Ni sites strengthened the transition metal–oxygen bond and mitigated charge compensation effects, thereby improving the structural stability of the layered framework. The optimized LNO electrode, doped with 2 wt.% La, delivered a reversible specific capacity of approximately 160 mAh g^−1^ after 100 cycles at 1 C, along with excellent capacity retention of 94.2% (Figure 7). Density functional theory (DFT) calculations further confirmed the beneficial role of La doping on the electronic structure of LNO. Specifically, substituting Ni with La reduced the space-charge polarization within the La–O bonds and shifted the d-band center of La-doped LNO toward higher energy levels, thereby contributing to enhanced lattice stability and improved electrochemical performance [26].

*Critical assessment.* Despite its high theoretical gravimetric capacity (≈275 mAh g^−1^) and cobalt-free composition, LiNiO_2_ remains fundamentally limited by the persistent phenomenon of Ni^2+^/Li^+^ cation mixing—driven by the similar ionic radii of Ni^2+^ and Li^+^—which is difficult to eliminate completely using conventional synthesis methods such as co-precipitation or the sol–gel process [23]. Although numerous doping strategies have been proposed to alleviate this limitation, their effectiveness depends heavily on synthesis conditions, and direct comparison among studies is often complicated by the diversity of manufacturing and testing protocols. Consequently, the scale-up and long-term efficacy of these modification strategies remain major unresolved challenges.

#### 3.1.3. Lithium Manganese Oxides (LMnO)

Lithiated manganese oxide (LiMnO_2_, LMnO) has attracted significant interest as a cathode material due to the natural abundance, low cost, and eco-friendliness of manganese-based compounds. In addition to these advantages, LMnO is electrochemically active and exhibits a high theoretical capacity of approximately 285 mAh g^−1^. Structurally, LiMnO_2_ can exist in two principal polymorphic forms: an orthorhombic “zigzag” phase with *Pmnm* symmetry and a monoclinic phase with *C*2*/m* symmetry. To improve structural stability during cycling and address issues associated with low crystallinity, considerable effort has been devoted to synthesizing stoichiometric layered LMnO materials. However, as with LNO, obtaining phase-pure LMnO with a stable layered structure remains a challenge. Furthermore, compared to LCO, LMnO exhibits lower structural stability during the charging process, primarily due to the tendency of manganese ions to undergo structural rearrangements and phase transformations upon lithium extraction. These instabilities impair cycling performance and have limited the large-scale practical application of layered LMnO cathodes [26,27].

**Figure 8:**
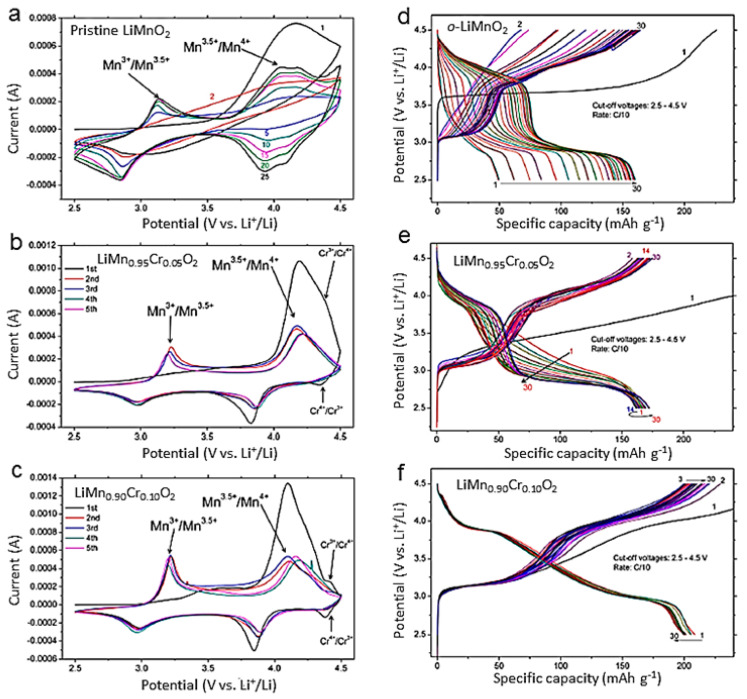
Electrochemical performance of the layered Cr-doped LiMn_1−x_Cr_x_O_2_ cathode materials. Cyclic voltammograms of the three-electrode cells, composed of (**a**) pristine LiMnO_2_, (**b**) 5% Cr-doped, and (**c**) 10% Cr-doped cathodes, performed between 2.5 and 4.5 V at a potential scan rate of 0.1 mV s^−1^. Charge/discharge profiles of the coin-type cells composed of (**d**) pristine LiMnO_2_, (**e**) 5% Cr-doped, and (**f**) 10% Cr-doped LiMnO_2_ cathodes cycled at C/10 rate with cut-off voltages of 2.5 and 4.5 V. Reproduced from [30]. Copyright 2013 Elsevier.

Layered LiMnO_2_ also suffers from severe structural distortion during electrochemical cycling, primarily due to the Jahn–Teller activity of Mn^3+^ ions. During lithium extraction, the layered framework tends to transform irreversibly into spinel-like phases, resulting in rapid capacity fading and poor cycling stability. As illustrated in Figure 8, these structural transformations are accompanied by significant lattice rearrangements that deteriorate lithium-ion diffusion pathways and reduce electrochemical reversibility [28,29,30]. To address these shortcomings, various approaches have been explored, including cation substitution, nanostructuring, and surface modification. Partial substitution of Mn with transition metals such as Ni, Co, Cr, or Fe has been shown to inhibit Jahn–Teller distortion and stabilize the layered structure during cycling. Furthermore, reducing particle size and engineering surface coatings can enhance lithium diffusion kinetics and minimize undesirable side reactions with the electrolyte. These strategies have contributed to improved cycling performance and enhanced structural stability, thereby increasing the potential of manganese-based layered oxides for next-generation lithium-ion batteries. Despite these efforts, the intrinsic limitations of LMnO have hindered its large-scale commercialization. Consequently, significant research has focused on developing binary and ternary transition-metal oxide systems that combine the strengths of LCO, LNO, and LMnO, while mitigating their individual drawbacks. These multi-component cathode materials offer a promising pathway toward high-performance, cost-effective, and thermally stable LIB technologies.

*Critical assessment.* Although LiMnO_2_ represents an attractive cobalt-free and low-cost alternative cathode, its practical application is fundamentally limited by thermodynamic instability; this leads to a gradual transformation into a spinel-like phase during cycling, resulting in capacity fading and voltage decay. While cation substitution (Ni, Co, Cr, Fe) has proven effective in mitigating this degradation, the reported improvements vary markedly depending on dopant concentration, crystalline phase, and synthesis conditions, making direct comparisons between studies difficult. Consequently, developing a universally effective strategy to stabilize the layered LiMnO_2_ framework remains a major challenge.

#### 3.1.4. Mixed Transition-Metal Oxide (LiNi_1−x_M_x_O_2_, *M* = Co, Mn)

Significant effort has been devoted to developing mixed transition-metal layered oxides—obtained by partially substituting nickel with monovalent or multivalent cations—to improve structural and thermal stability, electrochemical performance, and cycling behavior. Among these materials, LiNi_0.5_Mn_0.5_O_2_, first reported by Tsutomu Ohzuku and his colleagues [31], is an equimolar solid solution of LiNiO_2_ and LiMnO_2_. Compared to LiNiO_2_, it exhibits improved thermal stability and cycling performance, while maintaining a high operating voltage and reversible capacity [32]. In this structure, Ni^2+^ acts as an electrochemically active species, whereas Mn^4+^ stabilizes the layered framework during repeated lithium insertion and extraction processes [33]. This material operates within a voltage range of 3.6 to 4.3 V, delivering capacities of approximately 175 to 250 mAh g^−1^ depending on the upper cut-off voltage [34].

Despite these advantages, advanced characterization techniques have revealed partial Li/Ni cation mixing, with approximately 8–10% of Ni^2+^ occupying lithium sites [35,36,37,38]. This cationic disorder hinders lithium-ion diffusion, thereby reducing rate capability (power) and long-term cycling stability. Nevertheless, LiNi_0.5_Mn_0.5_O_2_ still outperforms LiCoO_2_ in terms of energy density and thermal stability, maintaining structural integrity up to approximately 300 °C [39,40]. Consequently, several strategies have been explored to mitigate this cation mixing, notably ion-exchange synthesis, which reduces Li/Ni disorder to approximately 4% and significantly improves electrochemical performance [41,42]. Similarly, Al^3+^ doping enhances structural stability and increases discharge capacity, while lithium-rich compositions (Li_1+x_Ni_0.5_Mn_0.5_O_2+δ_) form Li_2_MnO_3_-like domains; the latter activate Mn redox reactions, enabling capacities of approximately 200 mAh g^−1^ with excellent stability over 100 cycles [43].

**Figure 9:**
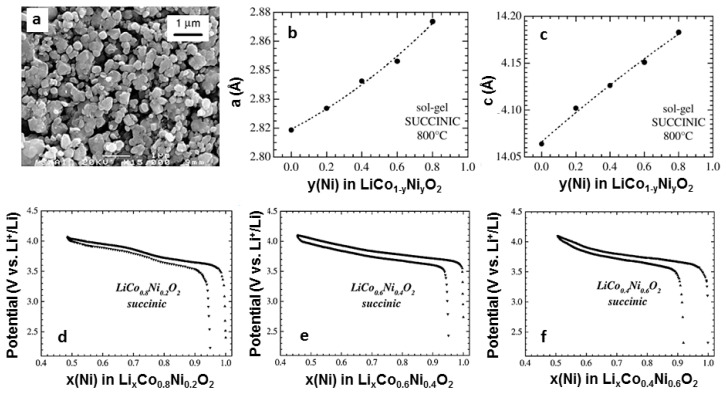
(**a**) Typical SEM image of LiCo_0.2_Ni_0.8_O_2_ synthesized by wet chemistry via a succinic acid-assisted method. (**b**,**c**) Variation in the lattice parameters a and c with Ni content. (**d**–**f**) Galvanostatic discharge–charge curves recorded at a C/10 rate in the potential range 2.5–4.1 V for LiCo_1−y_Ni_y_O_2_ (**d**) *y* = 0.2, (**e**) *y* = 0.4, and (**f**) *y* = 0.6. Reproduced from [44]. Copyright 2001 Elsevier.

**Figure 10:**
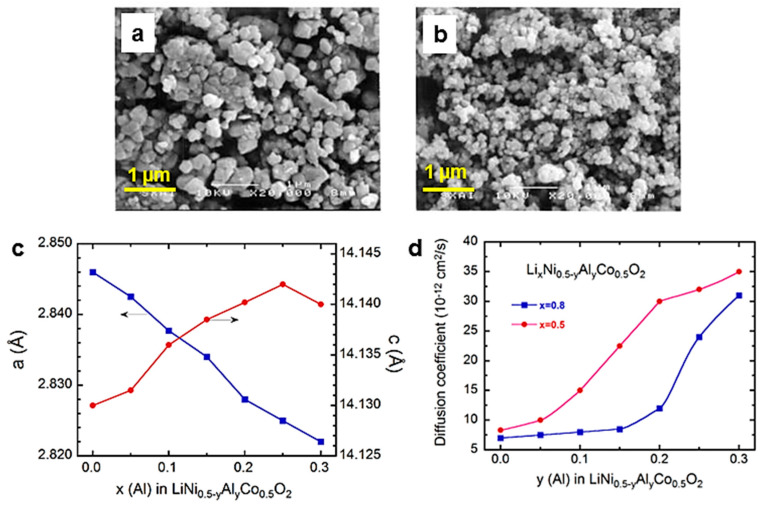
SEM micrographs of LiNi_0.5_Co_0.5_O_2_ (**a**) and LiNi_0.2_Al_0.3_Co_0.5_O_2_ (**b**) showing the decrease in particle size. (**c**) Variation in the lattice parameters with Al dopant content. (**d**) Evolution of the Li^+^ diffusion coefficient of Li_x_Ni_0.5−y_Al_y_Co_0.5_O_2_ with *x*(Li) and *y*(Al). Reproduced from [46]. Copyright 2003 Elsevier.

To further overcome the limitations of LiCoO_2_ and LiNiO_2_, considerable attention has also been devoted to LiNi_1−y_Co_y_O_2_ solid solutions synthesized via wet-chemical methods, which offer improved compositional homogeneity and crystallinity (Figure 9) [44]. Among the investigated compositions, LiCo_0.6_Ni_0.4_O_2_ exhibited a reversible capacity of approximately 150 mAh g^−1^ within the 2.5–4.1 V voltage range. Further improvement was achieved through substitution with Al^3+^ ions; due to their smaller ionic radius compared to Ni^3+^ ions, these ions enhance structural stability, limit degradation associated with overcharging, and increase the lithium insertion potential [45,46]. As illustrated in Figure 10, aluminum doping reduces particle size, alters the lattice parameters through successful incorporation into the host structure, enlarges the interlayer spacing, and facilitates lithium-ion diffusion; this results in improved electrochemical kinetics and discharge capacities of approximately 115 mAh g^−1^ at a cut-off voltage of 4.2 V.

*Critical assessment.* LiNi_0.5_Mn_0.5_O_2_ and the related series such as LiCo_1−y_Ni_y_O_2_ (including Al-doped variants) highlight a recurring challenge associated with low-cobalt layered cathodes: the persistent phenomenon of Ni^2+^/Li^+^ cation mixing—typically estimated at 8–10% for materials synthesized via conventional methods—which remains difficult to eliminate [36,37,38]. Although ion exchange, Al doping, the use of Li-excess compositions, and particle size reduction have each demonstrated the ability to mitigate cation disorder or enhance electrochemical performance [41,42,43,44,45,46], these approaches invariably entail trade-offs between structural order, capacity, process complexity, and industrial scalability. Consequently, no single modification strategy has yet succeeded in simultaneously reconciling minimized cation mixing, high reversible capacity, and a simple, reproducible synthesis route suitable for large-scale production.

#### 3.1.5. Ternary Transition-Metal Oxides (LiNi_1−x−γ_Mn_x_Co_γ_O_2_, NMC)

**Figure 11:**
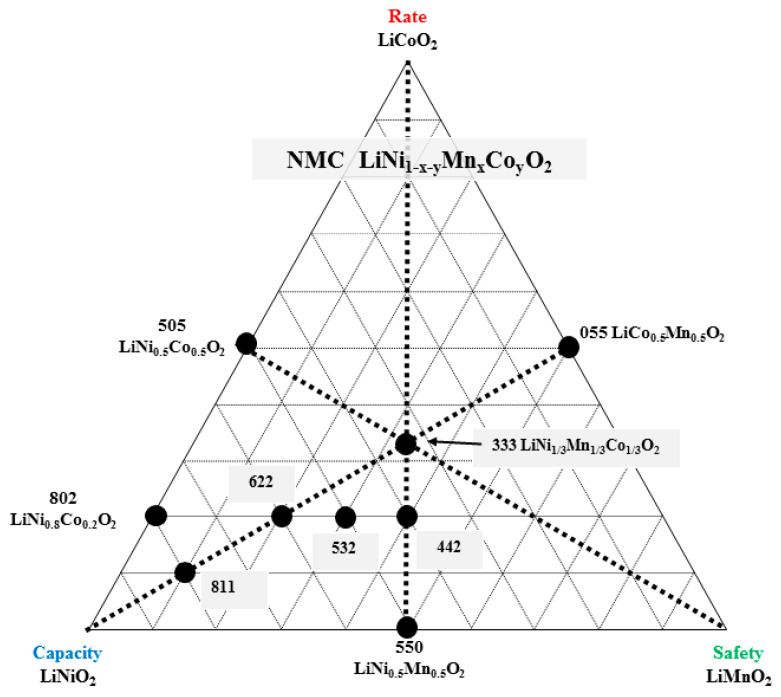
Ternary phase diagram of LiNi_1−x−y_Mn_x_Co_x_O_2_ formed from LiCoO_2_-LiNiO_2_-LiMnO_2_ solid solution.

To overcome the limitations associated with single-transition-metal layered oxides, Ohzuku et al. [31] reported in 2001 the successful synthesis of a ternary solid solution composed of three transition-metal ions—Co, Mn, and Ni—in equal proportions, corresponding to the general formula LiNi_1−x−γ_Mn_x_Co_γ_O_2_ (designed as NMC). The design of this solid solution was based on the triangular phase diagram of the LiCoO_2_–LiNiO_2_–LiMnO_2_ system, leading to the formation of a layered two-dimensional (2D) crystal structure, as shown in Figure 11. The development of NMC materials was primarily driven by the need to synergistically combine the advantages of Ni-, Mn-, and Co-based oxides, while simultaneously alleviating their individual limitations. Electrochemical studies revealed that LiNi_1/3_Mn_1/3_Co_1/3_O_2_ (NMC333) operates at a high average voltage of approximately 4.7 V and exhibits outstanding electrochemical performance. Specifically, NMC333 delivered a reversible capacity of 160 mAh g^−1^ within the 2.5–4.4 V voltage window, a figure that reached nearly 200 mAh g^−1^ during cycling between 2.8 and 4.6 V [47].

In this system, each transition-metal ion plays a specific and complementary role:

- Manganese (Mn), owing to its low cost and environmental benignity, primarily enhances the structural stability of the cathode during cycling.
- Nickel (Ni) constitutes the main electrochemically active species; it increases the specific capacity through the Ni^2+^/Ni^4+^ redox couple.
- Cobalt (Co) plays a critical role by mitigating Li/Ni cation mixing—an effect impossible to eliminate entirely—thereby improving reversibility and capacity retention [48,49,50].

The synergistic interaction between these three transition metals has made NMC cathodes one of the most versatile and commercially successful families of materials for LIBs, offering an effective balance of energy density, service life (cycle life), cost, and safety.

**Figure 12:**
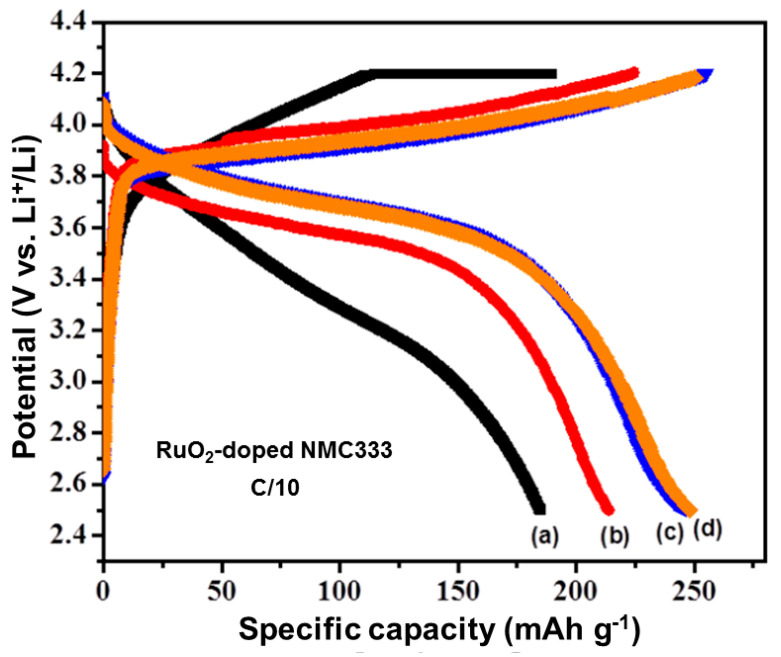
Galvanostatic charge/discharge test of (a) pristine NMC333, (b) 1% RuO_2_ doped NMC333, (c) 2% RuO_2_ doped NMC333, and (d) 3% RuO_2_ doped NMC333 at 0.1 C rate. Reproduced from [52]. Copyright 2021 under a Creative Commons Attribution 4.0 International License.

During the charging process, lithium ions are extracted from the layered structure through the oxidation of Ni^2+^ to Ni^4+^ (via the intermediate Ni^3+^) and the oxidation of Co^3+^ to Co^4+^. In contrast, Mn^4+^ remains electrochemically inactive and primarily contributes to structural stabilization during cycling. This redox behavior enables NMC333 to exhibit high reversible capacity and stable discharge performance over long cycling periods, even at elevated temperatures such as 55 °C. However, it has been reported that prolonged aging (45 days at 70 °C) of lithium-deficient NMC333 triggers a phase transition from the layered structure to a spinel-type phase, thereby deteriorating its electrochemical performance [51]. To mitigate these degradation mechanisms, various material engineering strategies have been extensively explored, including cationic doping, surface coating, and particle size optimization. These modifications aim to stabilize the layered structure, inhibit detrimental phase transitions, minimize cation mixing, and ultimately enhance the electrochemical stability of NMC cathodes during prolonged cycling. Such approaches are particularly important for meeting the stringent performance and durability requirements of electric vehicle applications. Among these strategies, RuO_2_ doping has proven particularly effective in enhancing the electrochemical performance. As illustrated in Figure 12, the pristine NMC333 electrode delivered an initial discharge capacity of 194.9 mAh g^−1^, whereas electrodes doped with 1, 2, and 3 wt.% RuO_2_ exhibited significantly higher capacities, reaching 214.9, 242.9, and 251.2 mAh g^−1^, respectively. In addition to the substantial increase in specific capacity, the sample containing 3 wt.% RuO_2_ demonstrated markedly improved cycling stability compared to the other compositions. These results highlight the critical role of targeted cationic doping in simultaneously enhancing capacity retention and long-term structural durability in NMC cathode materials [52].

*Critical assessment.* Although NMC333 provides a favorable trade-off between capacity, cycling stability, and thermal safety, raising the upper cut-off voltage to achieve higher specific capacities inevitably accelerates surface degradation, structural instability, and phase transformation (shifting from a layered structure to a spinel-like structure) during prolonged high-temperature storage, thereby compromising long-term electrochemical performance [51]. Furthermore, the exceptionally high capacities reported for modified NMC333 compositions must be interpreted in light of the associated cycling stability, Coulombic efficiency, and testing conditions, as an increase in initial capacity does not necessarily translate into sustained electrochemical performance. Consequently, optimizing NMC333 requires striking a balance between energy density and long-term structural and interfacial stability, rather than focusing solely on maximizing reversible capacity.

#### 3.1.6. High-Voltage Layered Cathode Materials (>4 V)

**Figure 13:**
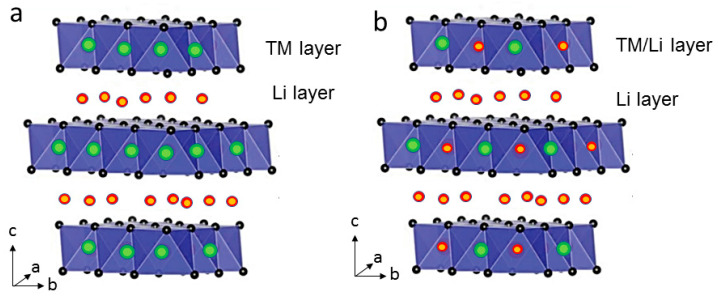
Structural components of the Li-rich cathode material, represented as a composite between the (**a**) Li*M*O_2_ (*M* = Co, Mn, Ni) phase and (**b**) Li_2_MnO_3_ phase.

Lithium-rich (e.g., Li_1+x_Mn_0.54_Co_0.13_Ni_0.13_O_2_) and nickel-rich (e.g., LiNi_0.8_Mn_0.1_Co_0.1_O_2_; commonly referred to as LLMCN and NMC811, respectively) layered oxides are emerging as promising next-generation cathode materials for LIBs. These materials can operate at high cut-off voltages exceeding 4 V and, when cycled within the 2.4–4.7 V voltage window, offer several significant advantages: reduced cobalt content, improved cost-effectiveness, enhanced safety, high energy density, and remarkable specific capacities approaching 260 mAh g^−1^. Their reversible capacity is nearly double that of conventional LiCoO_2_ [53,54,55,56]. Lithium-rich layered oxides are generally described as having a composite (or two components) structure comprising a rhombohedral layered Li*M*O_2_-type framework (*M* = Co, Mn, Ni; space group *R*$\bar{3}$*m*) associated with a monoclinic Li_2_MnO_3_-type (space group *C*2*/m*), as illustrated in Figure 13. The synergistic interaction between the Li*M*O_2_ layered framework and the ordered LiMn_2_O_3_-type domains is largely responsible for the high capacity and enhanced electrochemical performance of these materials.

Despite their high specific capacity, these materials present several significant limitations. When charged to voltages approaching 4.6 V, they undergo irreversible capacity loss and display low Coulombic efficiency during the initial cycles. This behavior is primarily attributed to the formation of Li_2_O, resulting from the simultaneous extraction of lithium and oxygen from the electrochemically inactive Li_2_MnO_3_ phase. The resulting structural instability leads to poor cycling stability and significant voltage drop—a phenomenon driven by the gradual transition from the layered phase to a spinel-like phase, ultimately causing a decline in capacity [57]. Furthermore, the dissolution of manganese into the electrolyte during cycling further compromises the long-term electrochemical stability and overall performance of these materials. To overcome this limitation, considerable effort has been devoted to optimizing various synthesis methods—such as co-precipitation, the sol–gel process, solid-state reaction, hydrothermal synthesis, and combustion—which are reviewed and critically analyzed in Ref. [58]. These challenges underscore the need for continued research into structural stabilization strategies—such as doping, surface modification, and interface engineering—to fully harness the potential of lithium-and nickel-rich layered oxides as high-capacity cathodes for advanced LIB applications.

It should be emphasized that, according to the most reliable studies conducted to date using diffraction, pair-distribution-function analysis, and atomic-resolution microscopy, the rhombohedral Li*M*O_2_ (*R*$\bar{3}$*m*) and monoclinic Li_2_MnO_3_ (*C*2/*m*) constituents of Li-rich layered oxides generally cannot be distinguished as two physically separate crystalline phases via conventional laboratory X-ray diffraction [59,60,61]. Instead, the two components are intimately intergrown at the atomic scale and share a common close-packed oxygen sublattice; the Li_2_MnO_3_-type cation ordering typically manifests only as weak superlattice reflections in the 20–25° (2θ, Cu Kα) range, rather than as an independently indexable second phase [60]. Consequently, the common description in terms of a “composite” (or “two-phase” system) should be interpreted as a structural and compositional model—describing Li_2_MnO_3_-like honeycomb-ordered domains embedded within a single, integrated layered framework [59,61]—rather than as evidence of a true physical mixture of two crystallographically distinct and individually discernible phases. This distinction makes it possible to reconcile the terminology commonly adopted in the literature with the crystallographic observations derived from conventional X-ray diffraction.

A defining electrochemical characteristic of Li-rich layered cathodes—distinguishing them from the other high-voltage layered oxides discussed in this review—is the so-called “activation” process that occurs during the first few charge cycles when the upper cut-off voltage is extended to ≈4.8–4.9 V [62,63]. During this activation process, Li_2_MnO_3_-like domains, initially electrochemically inactive, undergo simultaneous lithium extraction and partial, largely irreversible oxygen release; this results in a characteristic voltage plateau near 4.5 V, which is absent in conventional layered oxides. Once activated, the material can deliver specific capacities in the range of 240–250 mAh g^−1^, which remain relatively stable during subsequent cycles. This activation process thus constitutes the most distinctive electrochemical feature of the Li-rich layered cathode family, fundamentally differentiating it from cathodes based on conventional layered oxides.

The origin of the capacity delivered by Li-rich layered oxides—beyond that accessible via conventional transition-metal redox reactions (i.e., exceeding the practical limit of ≈220–230 mAh g^−1^ associated with the complete Ni^2+^/Ni^4+^ and Co^3+^/Co^4+^ couples in typical Li- and Mn-rich Ni-Co-Mn oxide compositions, known as LLNMC)—has been the subject of numerous studies over the past decade [62,63,64,65]. It is now well established, through O K-edge X-ray absorption spectroscopy, resonant inelastic X-ray scattering (RIXS), X-ray photoelectron spectroscopy (hard/soft), and operando differential electrochemical mass spectrometry, that this additional capacity stems primarily from a reversible (or partially reversible) anionic redox process within the oxygen sublattice, rather than from an additional cationic redox couple. Consequently, capacities exceeding the practical transition-metal redox limit (≈220–230 mAh g^−1^) cannot be explained by cationic redox alone. This process is generally associated with the formation of localized hole states on O 2p orbitals and/or short-range peroxo-/superoxo-like (O–O)^n−^ species generated during the extensive delithiation of Li_2_MnO_3_-like domains; Li-O-Li configurations enable oxidation of the anionic lattice without a compensatory transition-metal oxidation step [62,64].

It is important to note that the oxygen redox process is an intrinsic feature of the Li-rich—dominated by Mn and exhibiting “honeycomb” ordering—a phenomenon observed even in undoped LLNMC compositions free of Ru or any other 4d/5d-type dopant [63,65]. As discussed in Section 3.1.5, modification with RuO_2_ further enhances the reversibility and kinetics of the oxygen redox process by suppressing irreversible oxygen loss and stabilizing the process itself, although it is not the source of the anomalously high capacity observed. Since the capacity derived from conventional transition-metal redox reactions is intrinsically limited by the number of redox couples involving accessible d-electrons, the reversible anionic redox process is currently regarded as the primary experimentally validated pathway for achieving higher energy densities in layered oxide cathodes [63]. Nevertheless, the practical implementation of this mechanism remains hindered by irreversible oxygen loss and gas evolution, transition-metal migration into the lithium layer, hysteresis and voltage decay, as well as the resulting structural instability. Consequently, achieving long-term stabilization of the reversible oxygen redox process—rather than merely demonstrating its existence—remains the major challenge for commercialization of Li-rich layered cathodes.

Several strategies have been proposed to address the intrinsic limitations of Li-rich layered cathode materials and enhance their electrochemical performance:

- *Surface coating*: The application of protective surface layers minimizes direct contact between the cathode and the electrolyte, thereby suppressing parasitic side reactions and improving cycling stability [58,66,67].
- *Nanosizing*: The synthesis of nanoscale particles through optimized preparation methods enhances reaction kinetics, shortens Li^+^ diffusion paths, and consequently improves rate capability [68,69].
- *Cationic doping*: The incorporation of divalent or multivalent cations such as Mg^2+^, Zn^2+^, or Mo^6+^ into the crystal lattice enhances electronic conductivity and promotes Li^+^ diffusivity, contributing to improved structural stability during cycling [70,71,72].

Additional strategies have also been reported to further enhance the electrochemical performance of Li-rich layered cathode materials:

- *Anion substitution*: several studies [73,74] have shown that partial substitution of oxygen with fluorine during the first cycle decreases the amount of Li_2_O extracted from the Li_2_MnO_3_ phase, thereby improving Coulombic efficiency and mitigating irreversible capacity loss.
- *Alkali metal substitution*: Replacing a fraction of Li^+^ ions with larger monovalent cations such as Na^+^ [75] or K^+^ [76] expands the Li^+^ diffusion channels, enhances segregation between alkali and transition-metal ions, and improves cycling stability as well as rate capability. In particular, partial substitution with Na^+^ (ionic radius 1.02 Å compared to 0.76 Å for Li^+^) strengthens Li–O bonding, suppresses oxygen release, and enlarges the interlayer spacing, thereby facilitating Li^+^ diffusion and improving electrochemical performance [72].
- *Advanced coatings*: Although Al- and Cr-based coatings have been reported to increase rate capability, they do not fully prevent voltage fade, which is primarily associated with bulk structural changes [77]. More promising results have been achieved with AlF_3_ coatings, which provide both structural protection and enhanced electrochemical performance. For example, Li_1.2_Ni_0.2_Mn_0.6_O_2_ coated with AlF_3_ exhibited stable capacities of approximately 250 mAh g^−1^ after 50 cycles, together with improved rate capability, using a simple and scalable synthesis approach [58].

**Figure 14:**
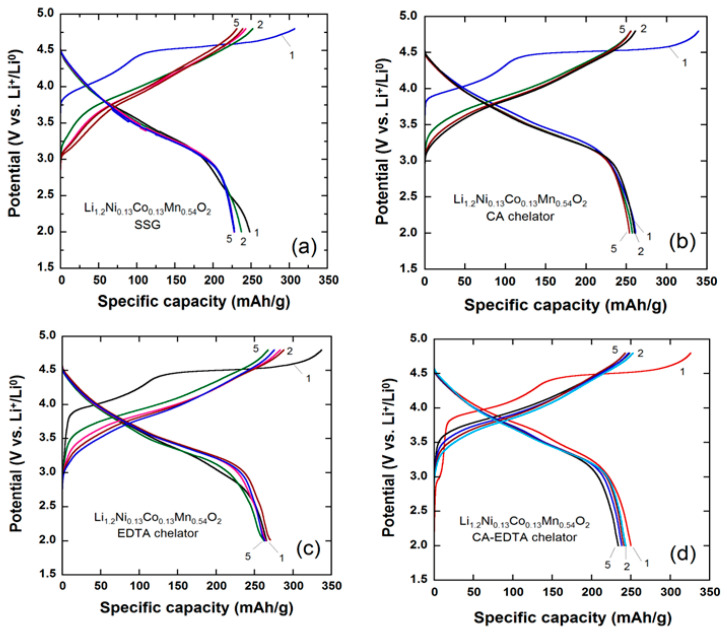
Galvanostatic charge–discharge profiles of nanostructured Li_1.2_Ni_0.13_Mn_0.54_Co_0.13_O_2_ electrodes synthesized by wet chemistry using different chelating agents: (**a**) self-sol–gel (SSG), (**b**) citric acid (CA), (**c**) EDTA, and (**d**) CA-EDTA. Electrochemical tests were carried out at a 0.1 C rate in the potential range between 2.0 and 4.8 V vs. Li^+^/Li. Reproduced from [69]. Copyright 2020 Springer.

Overall, these modification strategies highlight the crucial role of surface and interface engineering, crystal lattice doping, and structural stabilization in promoting the practical application of Li-rich cathode materials. Continued efforts in these areas are essential to mitigate voltage decay, reduce irreversible capacity loss, and improve long-term cycling stability, thereby paving the way for the development of next-generation, high-energy LIBs. For example, Abdel-Ghany and co-workers examined the effect of carboxylic-acid-based chelating agents—specifically, a concentrated citric acid solution and an ethylene diamine tetra-acetic acid (EDTA) solution—on the particle size, morphology, and electrochemical performance of layered Li-rich Li_1.2_Ni_0.13_Mn_0.54_Co_0.13_O_2_ synthesized via the sol–gel method. Their results demonstrated that the sample prepared using EDTA as an organic complexing agent exhibited superior electrochemical performance, characterized by higher initial capacity and better rate capability (Figure 14) [69].

**Figure 15:**
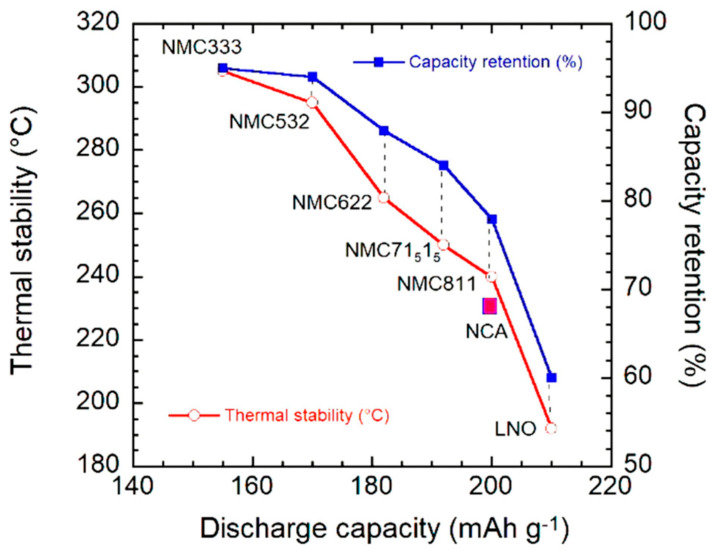
Illustration of the composition–performance relationship of NMC and NCA (red square) materials. Evolution of the thermal stability and capacity retention as a function of the discharge. Reproduced from [71]. Copyright 2020 under the terms of the Creative Commons Attribution (CC-BY) license.

Nickel-rich layered transition-metal oxides represent one of the most advanced families of cathode materials for high-energy LIBs. Their appeal stems from the high redox potential of the Ni^3+^/Ni^4+^ couple, which enables energy densities significantly higher than cobalt- or manganese-dominant compositions [78,79]. Common and important compositions, such as LiNi_0.8_Mn_0.1_Co_0.1_O_2_ (NMC811) and LiNi_0.8_Co_0.15_Al_0.05_O_2_ (NCA), adopt an O3-type layered structure—isostructural with LiCoO_2_—in which lithium layers alternate with *M*O_2_ slabs [78,80]. Figure 15 illustrates the relationship between composition and performance for NMC materials, emphasizing that the choice of cathode requires a careful balance between specific capacity and thermal stability [78]. The data also highlight the critical role of nickel content: Ni-rich formulations consistently achieve higher specific capacity and energy density than compositions with lower Ni content, such as NMC532 or NMC622 [79,81].

Among these materials, NCA has rapidly advanced toward large-scale applications, particularly in the electric vehicle (EV) sector, thanks to a favorable combination of high energy density, reduced cost (achieved by minimizing cobalt content), and enhanced chemical stability resulting from Al substitution [78,82]. Aluminum, with an ionic radius comparable to that of Ni^3+^ and Co^3+^, can be readily incorporated into the transition-metal sites of the NCA crystal structure, where it serves as a structural stabilizer. By occupying these sites, Al strengthens the crystal framework, improves structural durability, and helps maintain the high operating voltage associated with nickel redox activity [78]. This beneficial role of Al has led to the widespread adoption of NCA compositions in battery systems for EVs.

Despite these advantages, Ni-rich oxides remain susceptible to several intrinsic degradation mechanisms. The relatively weak Ni–O bonding and the highly oxidizing nature of the Ni^4+^ state increase chemical reactivity, thereby accelerating oxygen release, surface reconstruction into spinel- or rock-salt-type phases, and parasitic reactions with the electrolyte [83,84]. Furthermore, high-voltage cycling (>4.2 V) or high-temperature operation promotes the H2 → H3 phase transition, leading to significant crystal lattice contraction along the c-axis and causing microcrack formation within secondary particles [85]. These microcracks facilitate electrolyte infiltration and accelerate the growth of resistive cathode–electrolyte interphase (CEI) layers, ultimately hindering Li^+^ diffusion and compromising long-term capacity retention [86,87,88,89,90].

Studies conducted by Ryu et al. [89] demonstrated that the H2 → H3 phase transition is highly sensitive to nickel content. Specifically, only compositions containing at least 80% Ni exhibited a pronounced H2 → H3 transformation, while the associated d*Q*/d*V* peaks increased progressively with the Ni fraction, reflecting an increasingly pronounced structural response at high states of charge. Consistent with this trend, the LiNi_0.95_Co_0.025_Mn_0.025_O_2_ material displayed the largest variation in the “c” lattice parameter, indicating heightened lattice instability. Further work by Nam et al. [90] showed that both the onset potential and the kinetics of the H2 → H3 transition are accelerated as the Ni content increases. For instance, in LiNi_0.95_Co_0.04_Al_0.01_O_2_ (NCA95) material, the H3 phase emerged at approximately 4.17 V—earlier than in lower-Ni counterparts—and the transition was completed before 4.23 V. Overall, these observations highlight the earlier onset, faster progression, and increased structural severity of the H2 → H3 transformation in highly Ni-enriched cathodes.

To overcome these limitations, advanced strategies for surface engineering and bulk material modification have been developed. Surface coatings—including metals and metal fluorides (e.g., Ag, AlF_3_, MgF_2_) [91,92], metal oxides (e.g., TiO_2_, Al_2_O_3_, ZrO_2_, Y_2_O_3_) [93,94,95], phosphates (Li_3_PO_4_, FePO_4_), and lithium-conducting ceramics (e.g., Li_2_TiO_3_, LiAlO_2_) [91,92,93,94,95,96]—have been shown to inhibit electrolyte decomposition, delay oxygen release, and stabilize the cathode surface during high-voltage operation [91,95,97]. These coatings significantly enhance cycling performance and thermal stability by mitigating surface-induced phase transformations and reducing transition-metal dissolution [93].

In addition to surface coatings, bulk doping provides an intrinsic stabilization strategy for layered-structured cathodes. Substitution with cations such as Al, Ti, Zr, K, or Cr strengthens the layered framework [98,99,100,101], limits the Ni/Li cation mixing, and enhances structural reversibility during high-voltage cycling [100]. For instance, Ti and Zr dopants have been shown to significantly improve capacity retention by preserving structural integrity and mitigating surface degradation, even when cells are cycled up to 4.5–4.7 V [102,103]. Overall, these dopants contribute to enhanced cycling stability and extended battery lifespan. However, some of them—notably Ti, Al, and Zr—complicate the synthesis of the hydroxide/carbonate precursors via co-precipitation. Consequently, they are introduced using a post-wet sol–gel process [103,104,105,106,107,108,109,110,111]. Incorporating these dopants during calcination is not only costly, but can also lead to inhomogeneities or even surface phase segregation in some cases [112,113,114,115,116]. In contrast, Mg is easier to incorporate through a hydroxide co-precipitation synthesis, and is less expensive. For this reason, it is widely used as a dopant improving the cycle life and the thermal stability in all the lamellar compounds, such as LiCoO_2_ [117], LiNiO_2_ [114,115,116,118,119,120,121], NMC [118,122,123], NCA [120,121,124]. However, since Mg is electrochemically inactive, its use leads to a reduction in capacity. This decrease is negligible if the Mg content is limited to 1–1.5 mol%; nevertheless, in this case, the capacity loss of Ni-rich cathode materials remains a challenge that has not yet been fully resolved [123].

*Critical assessment.* Although Li-rich layered oxides offer the highest reported capacities among layered cathodes (<200 mAh g^−1^), these values are generally achieved only during the initial cycles and within extended voltage ranges (>4.5 V) that do not reflect actual operating conditions. Furthermore, irreversible first-cycle capacity loss, continuous voltage decay, and Mn dissolution—phenomena consistently reported in various independent studies [57]—indicate that the truly usable long-term capacity is substantially lower than the values commonly advertised. Consequently, the commercialization of these oxides will require simultaneous mitigation of surface oxygen loss and bulk cation migration, as neither strategy alone is sufficient to ensure long-term electrochemical stability.

#### 3.1.7. Core–Shell Structures and Concentration Gradient Designs in NMC and NCA

The degradation mechanisms discussed in the preceding sections—notably microcracking associated with the H2 → H3 phase transition, the high surface reactivity of Ni^4+^ species, and the growth of the cathode–electrolyte interphase (CEI)—originate primarily at or very near the particle surface. These phenomena are further exacerbated by the Ni-rich bulk compositions required to achieve high specific capacities. Although surface coatings and intraparticle doping have demonstrated the ability to improve electrochemical performance, they do not fully address the fundamental trade-off between the need for a nickel-rich core, which is highly electrochemically active, and a chemically stable surface. Consequently, strategies involving the engineering of internal particle composition have been developed to spatially decouple electrochemical activity from chemical stability within individual secondary particles themselves. Among these strategies, “core–shell” and “concentration gradient” architectures have proven particularly effective. These designs are based on a common principle: a Ni-rich core provides high capacity through the Ni^2+^/Ni^4+^ redox couple, while a peripheral zone enriched with manganese or cobalt enhances structural integrity, thermal stability, and resistance to electrolyte-induced surface degradation [100,125,126,127,128,129,130,131].

- Discrete Core–Shell Structures

The first implementation of compositional control within the particles themselves was the discrete “core–shell” structure with a sharp interface reported by Sun et al. [128]. In this configuration, a Ni-rich core, Li[(Ni_0.8_Co_0.1_Mn_0.1_)_0.8_(Ni_0.5_Mn_0.5_)_0.2_]O_2_, is encapsulated by a Mn-rich shell based on LiNi_0.5_Mn_0.5_O_2_. The Ni-rich core provides high reversible capacity, while the Mn-rich shell enhances thermal stability and mitigates parasitic reactions between the cathode surface and the electrolyte. Compared to Ni-rich cathodes with homogeneous composition, this architecture demonstrated improved thermal stability as well as enhanced cycling performance under moderate testing conditions [125,126].

**Figure 16:**
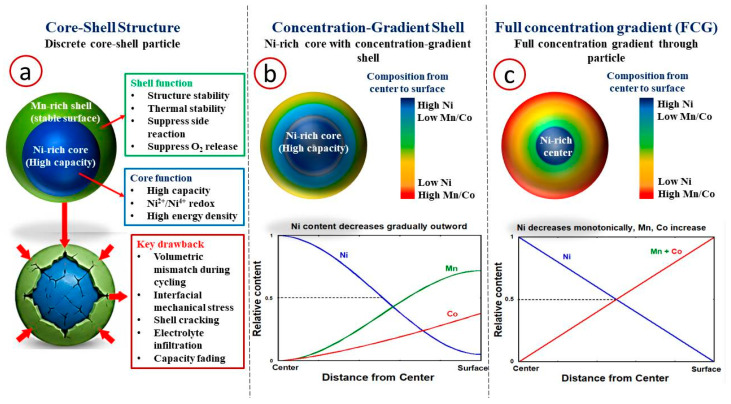
Schematic illustration of the three generations of intraparticle compositional engineering in Ni-rich layered cathode particles. (**a**) First-generation discrete core–shell structure with an abrupt Ni-rich/Mn-rich interface; the red arrow indicates the region of high interfacial stress and the primary crack-initiation site during cycling. (**b**) Second-generation concentration gradient structure with a gradual compositional transition confined to the outer shell of a Ni-rich core particle; the Mn-rich surface layer suppresses oxygen release and maintains Ni-redox reversibility. (**c**) Third-generation full-concentration-gradient structure showing a continuous, monotonic decrease in Ni content and corresponding increase in Mn + Co content from the particle center to the outer surface; radially aligned primary crystallites further reduce intergranular cracking.

Despite these advantages, discrete core–shell structures suffer from a fundamental limitation: the sharp compositional discontinuity at the core–shell interface generates substantial lattice mismatch and mechanical stress during repeated lithiation and delithiation. As the Ni-rich core and Mn-rich shell undergo different volume changes during cycling, interfacial stress progressively accumulates, leading to crack formation and long-term performance degradation (Figure 16a) [100,125,127]. Operando synchrotron X-ray imaging, combined with phase-field simulations, confirmed that this abrupt interface is the primary site for crack initiation under realistic cycling conditions [100]. Furthermore, an in-depth study conducted by Hou et al. [127] identified co-precipitation as the synthesis method best suited for the large-scale production of these core–shell cathodes, while highlighting that interfacial separation (delamination) posed a major obstacle to their commercialization. These limitations drove the development of particle architectures with compositional gradients, designed to reduce interfacial stresses and enhance structural durability.

- Concentration Gradient (CG) Structures

To address the limitations associated with the abrupt compositional transition in conventional “core–shell” architectures, concentration gradient (CG) structures have been developed. In these materials, the Ni content decreases gradually from the particle core toward the surface, while the Mn and Co concentrations increase correspondingly (Figure 16b). This continuous compositional gradient allows for a more homogeneous distribution of mechanical stresses within the particle, thereby reducing stress concentrations and inhibiting crack initiation and propagation during electrochemical cycling. Sun et al. [128] reported that CG LiNi_0.83_Co_0.07_Mn_0.10_O_2_ material exhibited enhanced structural stability and superior capacity retention compared to both conventional NMC622 and discrete core–shell cathodes.

The stabilizing role of the Mn-enriched surface in CG cathodes was further clarified by Bak et al. [129], who combined multiscale synchrotron spectroscopy with electrochemical analysis. Their study showed that the Mn-rich outer region promotes the reversibility of the Ni redox reaction during cycling, while inhibiting oxygen evolution at high states of charge. The Mn–O-rich surface layer acts as a protective chemical barrier, limiting parasitic electrolyte oxidation and inhibiting the formation of resistive rock-salt phases on the particle surfaces. This results in significant improvements in both cycling performance and thermal stability. These findings provide robust mechanistic evidence that surface Mn enrichment is an effective strategy for enhancing the long-term stability and safety of gradient-structured cathode materials.

Similar strategies based on a concentration gradient have also been successfully applied to NCA-based cathodes. Pan et al. [130] demonstrated that gradient Mg and Al co-doping within the LiNi_0.95_Co_0.03_Al_0.01_Mg_0.01_O_2_ material effectively mitigated the H2 → H3 phase transition, thereby enhancing structural stability during cycling. The modified cathode exhibited a capacity retention of 95.6% after 100 cycles and maintained excellent rate performance, delivering a capability of 172.9 mAh g^−1^ at 10C rate. Enriching the surface with Mg and Al—elements that are electrochemically inactive—passivates highly reactive surface sites, suppresses oxygen evolution, and improves interfacial stability, all while preserving the high Ni content in the particle core. Furthermore, a graded NCA–NMC core–shell architecture, consisting of an NCA core encapsulated within an NMC concentration gradient shell, was reported to achieve an outstanding capacity retention of 99.8% after 200 cycles, as well as significantly improved thermal and air stability compared to pristine NCA [131].

- Full-Concentration-Gradient (FCG) Structures

A more advanced gradient-design strategy is the full-concentration-gradient (FCG) architecture, in which the transition-metal composition varies continuously from the particle core to the surface, without forming distinct compositional interfaces [132]. In FCG cathodes, the Ni concentration decreases gradually from the center toward the surface, while the Mn and Co concentrations increase proportionally across the particle radius (Figure 16c). This continuous compositional transition minimizes local stress concentrations and promotes a more uniform distribution of mechanical strain during the repeated volume changes associated with Li^+^ insertion and extraction.

A notable microstructural characteristic of many FCG particles is the radial alignment of primary crystallites. This arrangement facilitates anisotropic Li^+^ transport along the (010) crystallographic planes, while reducing intergranular cracking as strains are absorbed through grain-boundary sliding rather than fracture [133]. Furthermore, the radial grain alignment shortens the effective Li^+^ diffusion pathways between grain boundaries and the particle surface, thereby enhancing rate capability. Park et al. [133] reported that FCG NMC78 (LiNi_0.78_Co_0.10_Mn_0.12_O_2_) retained 86.3% of its initial capacity after 4000 cycles at 0.5C, demonstrating that the synergistic integration of compositional-gradient engineering and microstructural control enables exceptional long-term cycling stability.

Beyond the compositional gradient, it has been demonstrated that a nickel oxidation-state (valence) gradient independently enhances the stability of Ni-rich cathodes. Lin et al. [134] synthesized a LiNi_0.8_Mn_0.1_Co_0.1_O_2_ material with a uniform composition that exhibited a hierarchical Ni valence gradient extending from the surface to the core. They demonstrated that surface enrichment of Ni^2+^ (lower valence), combined with a concentration of more oxidized Ni^3+^ ions in the interior, improves both cycling performance and thermal stability compared to conventional materials. These findings indicate that, in FCG cathodes, stabilization stems from the synergistic effect of the compositional and Ni-valence gradients. They further suggest that future cathode design could benefit from independent engineering of the valence profile, complementing the compositional gradient.

- Advanced Gradient Designs and Doping Strategies

Building on the concept of FCG, further structural and compositional refinements have been developed to optimize electrochemical performance and structural stability. In particular, dual-slope and continuous-concentration-gradient architectures allow for more precise control over the Ni distribution within secondary particles. This enables independent optimization of core and shell compositions, thereby improving the balance between capacity and long-term cycling stability. Such designs are especially relevant for highly Ni-rich cathodes (≥80% Ni), where the H2 → H3 phase transition occurring near the particle surface remains a dominant degradation mechanism, even in gradient-engineered structures [135].

Concurrently, elemental doping strategies have been widely integrated with FCG cathodes to further reinforce structural robustness and mitigate degradation pathways. For example, Zhao et al. [136] demonstrated that in situ Zr doping in LiNi_0.8_Co_0.05_Mn_0.15_O_2_-type FCG significantly improved electrochemical durability, achieving 91.9% capacity retention after 200 cycles at 1 C, while maintaining stability under high cut-off voltage (4.5 V) and elevated temperature conditions (55 °C). From a mechanistic standpoint, substitution with Zr^4+^ ions at transition-metal sites strengthens the layered structure by expanding the Li-slab spacing and suppressing irreversible phase transformations.

Furthermore, synergistic modification approaches combining FCG design, Ti-pillar doping, and Li_2_ZrO_3_ (LZO) surface coating have demonstrated additional gains in both Li^+^ transport kinetics and thermal stability. In this configuration, the LZO coating acts as a protective interfacial layer, effectively mitigating electrolyte-induced side reactions and surface degradation [137]. More recently, dual-doping strategies involving Zr combined with elements such as Al, Mg, or Ti have been proposed; these offer complementary enhancements in structural integrity, thermal resilience, and electrochemical kinetics [136,137].

A significant, though often overlooked, challenge in the synthesis of FCG materials lies in the thermal interdiffusion of transition metals during high-temperature lithiation; this phenomenon can diminish the as-synthesized gradient and weaken surface passivation. Cai et al. [138] demonstrated that SiO_4_^4−^ polyanion doping can suppress Ni and Mn interdiffusion during calcination, thereby preserving the concentration gradient structure and enhancing surface stability at the end of charge. This strategy highlights the importance of pairing gradient design with the use of structural stabilizers capable of resisting thermal homogenization.

The concentration gradient concept has also been extended to single-crystal Ni-rich cathodes, where surface degradation and intragranular stress accumulation remain key challenges. Engineering a low-Ni surface region within the single-crystal particles has been proposed to improve surface passivation and reduce electrolyte reactivity, while the absence of grain boundaries mitigates intergranular crack propagation [139]. Furthermore, in this work, Hu et al. further showed that integrating gradient engineering with single-crystal morphology represents a promising direction for next-generation Ni-rich cathodes, particularly for applications requiring both high energy density and long-term cycling stability.

Overall, core–shell and concentration gradient architectures represent major advances in the design of Ni-rich layered cathode materials. By spatially decoupling electrochemical functions within individual secondary particles themselves, these strategies enhance the trade-off between high energy density and long-term structural stability. Compared to early discrete core–shell configurations, CG and FCG structures ensure a more homogeneous stress distribution and improved resistance to particle cracking during electrochemical cycling. The ongoing integration of concentration gradient engineering with surface coatings, elemental doping, and single-crystal designs is expected to play a central role in the development of next-generation lithium-ion batteries for EV applications.

Another point warranting explicit discussion concerns the experimental verification of the gradients themselves. Rigorous confirmation of a continuous concentration gradient, as opposed to a nominal target profile inferred from the co-precipitation feed protocol, requires compositional characterization with sub-particle spatial resolution. Such evidence is typically obtained via line scans or elemental mapping using scanning transmission electron microscopy coupled with energy-dispersive X-ray spectroscopy (STEM-EDS) on particle cross-sections, electron probe microanalysis (EPMA), atom-probe tomography (ATP), or, where appropriate, depth-resolved X-ray photoelectron spectroscopy (XPS). Although several studies on CG and FCG materials summarized earlier present this level of direct structural evidence (e.g., the multiscale synchrotron spectroscopy mapping in [129] and the microstructure-resolved analysis in Ref. [100]). Many published studies continue to infer the existence and shape of the concentration gradient primarily from the intended co-precipitation feed profile together with indirect bulk characterization, such as broadened XRD reflection or gradual lattice-parameter variations obtained from Rietveld refinements of powder reflection data. While these techniques provide valuable structural information, they cannot, on their own, unequivocally distinguish a truly continuous compositional gradient from a multi-shell architecture with discrete compositional steps, nor can they exclude significant particle-to-particle compositional heterogeneity within the same powder batch. Consequently, although the electrochemical improvements reported for CG and FCG cathodes are generally well established, the proposed gradient architecture should be considered directly confirmed only when supported by compositional mapping resolved at the particle scale. In the absence of such evidence, the description in terms of a concentration gradient should instead be interpreted as a structural model based on the synthesis process; this model is consistent with available experimental data, without, however, being independently proven by them.

In light of these factors, alternative structural design strategies have also emerged to improve the mechanical stability of Ni-rich cathodes, while avoiding the synthetic complexity associated with concentration gradient engineering. The use of single-crystal Ni-rich particles, replacing conventional polycrystalline secondary agglomerates, represents an increasingly prominent example [140,141,142,143]. Single-crystal NMC and NCA particles, typically 1 to 5 µm in size, eliminate the internal grain-boundary network that provides preferential pathways for intergranular crack propagation in conventional polycrystalline secondary particles. Consequently, they exhibit markedly reduced microcracking, a smaller electrode–electrolyte interfacial area, and superior capacity retention under high-voltage and elevated-temperature cycling [140,141]. These benefits are achieved without requiring the compositionally graded, multi-stage co-precipitation employed for CG and FCG cathodes, making single-crystal morphology an attractive and, in several respects, more synthetically accessible cobalt-free Ni-rich compositions [142]. However, the larger particle size of single-crystal materials generally leads to longer solid-state Li^+^ diffusion paths, while their synthesis often requires higher calcination temperatures and longer sintering times than polycrystalline counterparts. These factors remain important considerations for large-scale industrial production and must be weighed against the demonstrated improvements in mechanical and electrochemical stability [143].

Collectively, these insights highlight why Ni-rich layered oxides remain among the most promising cathode materials for next-generation lithium-ion batteries. Their high energy density and reduced reliance on cobalt strongly support the technological, economic, and sustainability requirements of large-scale energy storage applications, particularly electric vehicles. At the same time, continuous advances in surface coatings, elemental doping, and microstructural engineering are progressively mitigating key limitations related to thermal instability, interfacial reactivity, and long-term cycling durability.

*Critical assessment.* The evolution from “core–shell” to concentration gradient and, subsequently, full-concentration-gradient (FCG) architectures represents a significant advancement in mitigating interfacial stress and improving the cycling stability of Ni-rich layered cathodes. However, reported performance gains are generally benchmarked against conventional homogeneous polycrystalline materials rather than their highest-performing counterparts—such as single-crystal or dopant-stabilized materials—making it difficult to assess their true relative advantage. Furthermore, the precise multi-stage co-precipitation required to fabricate these gradient architectures increases synthesis complexity and raises concerns regarding large-scale manufacturing, process control, and batch-to-batch reproducibility. Consequently, the practical value of concentration gradient designs will ultimately depend not only on their electrochemical performance but also on the development of scalable, cost-effective, and industrially reproducible synthesis routes.

#### 3.1.8. Disordered Rock-Salt (DRS) Cathodes: An Emerging Cobalt-Free Alternative

The family of “disordered rock salt” (DRS) cathode materials has attracted increasing interest over the past decade, despite having received limited attention in earlier literature reviews. Unlike the layered oxides discussed throughout Section 3.1, DRS cathodes deliberately leverage cationic disorder rather than seeking to eliminate it: Li^+^ ions and transition-metal cations are randomly distributed over a single cubic rock-salt-type cationic sublattice (space group *Fm*-3*m*), thereby removing the distinction between lithium and transition-metal layers that characterizes conventional layered oxides [144].

Given that completely random cationic disorder would, in principle, significantly hinder Li^+^ ion transport due to the presence of transition-metal cations, effective lithium transport in DRS materials relies on a so-called “0-TM” (transition-metal-free) percolation network—consisting of diffusion channels reserved exclusively for lithium; this network becomes statistically accessible once the Li content exceeds a critical threshold (which generally requires a lithium-rich composition, such as Li_1+x_, combined with a certain degree of short-range cationic order) [144,145]. Consequently, lithium transport in DRS cathodes is fundamentally governed by percolation theory rather than the two-dimensional diffusion pathways characteristic of layered oxides, which explains the relatively poor rate capability observed in many early DRS compositions.

A major advantage of the DRS concept lies in its exceptional compositional flexibility. Since it is cationic disorder—rather than a specific arrangement of transition metals—that defines the crystal structure, DRS cathodes can be designed using abundant, cobalt-free first-row transition metals (such as Mn, Fe, Ti, and Ni) across a wide range of compositions. Like lithium-rich layered oxides (Section 3.1.6), many DRS materials utilize the lattice oxygen redox process to achieve reversible capacities exceeding 250–300 mAh g^−1^, thereby surpassing the capacity limits of conventional cation-redox-based cathodes [145]. However, these high capacities are frequently accompanied by voltage hysteresis, oxygen-related structural degradation, transition-metal migration, and sluggish Li^+^ ion transport kinetics due to percolation-limited diffusion pathways. Consequently, the use of nanoparticles, conductive carbon networks, fluorination, and short-range order engineering has emerged as a primary strategy for enhancing the electrochemical performance of DRS cathodes [144,145].

Despite their distinct crystal structures, DRS materials and lithium-rich layered oxides are increasingly viewed as complementary manifestations of the same fundamental strategy: leveraging lattice oxygen redox to overcome the energy density limits imposed by conventional metal-based redox chemistry. Accordingly, both material families face similar scientific challenges regarding oxygen redox reversibility, structural degradation, and long-term voltage stability.

*Critical assessment*. Despite their exceptional theoretical potential, DRS cathodes exhibit a significantly lower level of technological maturity than conventional layered oxides. Most reported electrochemical performance metrics have been demonstrated using laboratory-synthesized nanomaterials evaluated in half-cell configurations under optimized testing protocols. Furthermore, voltage hysteresis, slow Li^+^ ion transport kinetics, and structural degradation induced by the oxygen redox process continue to limit their actual energy efficiency and long-term cycling stability. Consequently, future progress will depend not only on achieving higher reversible capacities but also on reducing voltage hysteresis, improving energy efficiency, and developing synthesis processes scalable to an industrial level, all while preserving the unique structural characteristics of DRS materials.

### 3.2. LiMn_2_O_4_ (LMO) Spinel Oxides

In the early 1980s, Thackeray and co-workers [146] investigated cubic spinel-structured cathode materials with *Fd*$\bar{3}$*m* symmetry, including LiMn_2_O_4_ (LMO, ~4 V) and LiMn_1.5_Ni_0.5_O_2_ (~5 V), which offer three-dimensional (3D) lithium-ion diffusion pathways. Compared to layered oxide cathodes, spinel materials are generally safer, more cost-effective, and exhibit several favorable electrochemical characteristics, such as a high operating voltage plateau, excellent rate capability, and good cycling stability [147]. Nevertheless, spinel cathodes still experience significant capacity fading during long-term cycling. This performance mainly originates from two factors:

(1) Manganese dissolution into the electrolyte, caused by the disproportionation reaction of Mn^3+^ ions (2Mn^3+^ → Mn^4+^ + Mn^2+^). The generated Mn^2+^ species dissolve into the electrolyte, leading to the gradual loss of electrochemically active material.
(2) Irreversible structural distortion, involving the transformation of the cubic spinel structure into a tetragonal phase. This transition is associated with the presence of Jahn–Teller active Mn^3+^ ions, which induce lattice distortion and structural instability during repeated charge/discharge cycling [148,149].

**Figure 17:**
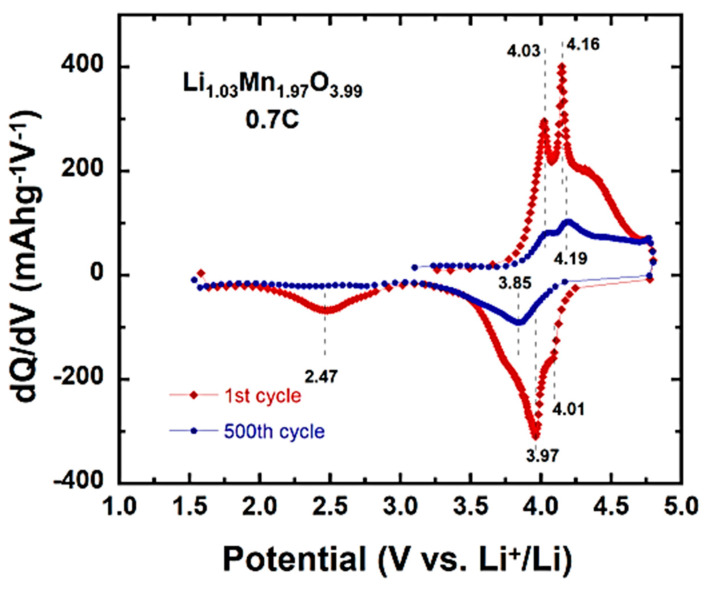
Differential capacity plots (d*Q*/d*V*) vs. *V* for the LLMO electrode tested at the 1st and 500th cycle in the potential window 1.5–4.8 V. The peaks at ~4 and ~3 V in the d*Q*/d*V* plots correspond to Mn^3.5+/4+^ and Mn^3+/3.5+^, respectively. Reproduced from [108]. Copyright 2024 under the Creative Commons Attribution 4.0 International License.

A study examined the electrochemical behavior of Li-rich spinel Li_1+y_Mn_2−y_O_4−δ_ (LLMO, y ≈ 0.03, δ ≈ 0.01) used as a cathode in Li-ion batteries, focusing particularly on the origin of the additional capacity obtained within an extended operating voltage window of 1.5 to 4.8 V vs. Li^+^/Li [137]. The Li-rich LLMO spinel material, synthesized via the sol–gel route and calcined at 900 °C, exhibited an average crystallite size of approximately 380 nm, a specific surface area of 1.68 m^2^ g^−1^, and monodisperse mesopores with an average diameter of 5.1 nm. The electrode delivered an initial discharge capacity of 172 mAh g^−1^. At a current density of 100 mA g^−1^ (~0.7 C), the capacity remained at 123 mAh g^−1^ after 100 cycles, corresponding to a retention of 71.5%. Remarkably, the electrode still delivered 77 mAh g^−1^ after 500 cycles within the wide potential range of 1.5–4.8 V, thereby demonstrating excellent long-term stability. This enhanced cyclability was attributed to the suppression of the Jahn–Teller distortion through Li doping. In contrast, during cycling within the narrower voltage range (3.0–4.5 V), the electrode exhibited a much lower initial capacity of 85 mAh g^−1^ and a capacity retention of only 54.3% after 100 cycles. Differential capacity analysis (d*Q*/d*V*) versus potential confirmed the structural and electrochemical stability of the Li-rich spinel. After 500 cycles, the redox peaks remained at virtually identical potentials and the peak separation showed no significant change, indicating that Li doping effectively mitigates the main mechanisms of electrode degradation and capacity loss (Figure 17) [108].

*Critical note.* The reported BET specific surface area (1.68 m^2^ g^−1^), combined with the theoretical density of the LiMn_2_O_4_ spinel (≈4.31 g cm^−3^), corresponds to an equivalent mean particle diameter of approximately 0.8 to 1.0 µm, based on the relationship d ≈ 6/(ρ·SSA). This value is significantly larger than the average crystallite size (approximately 380 nm) obtained via XRD/Rietveld analysis of the same material, indicating that the powder consists primarily of polycrystalline secondary particles in the micrometer range, formed by the aggregation of smaller primary crystallites. Consequently, it is more appropriate to describe this material as having a hierarchical micro/nanostructure, in which nanoscale primary crystallites are aggregated into micrometer-sized polycrystalline secondary particles; the latter exhibit internal grain boundaries and closed porosity that are largely inaccessible to the electrolyte. Thus, although the external particle size falls mainly within the micrometer range, the internal nanostructure can still influence electrochemical behavior through Li^+^ ion transport at the crystallite scale and grain-boundary effects within the secondary particles.

**Figure 18:**
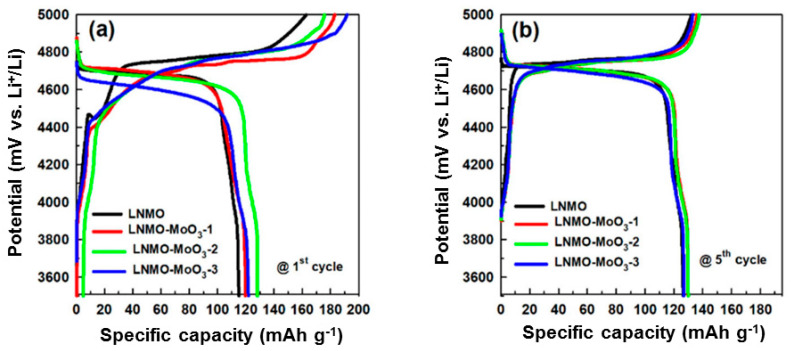
Charge–discharge curves of pristine LiNi_0.5_Mn_1.5_O_4_ and MoO_3_-coated LiNi_0.5_Mn_1.5_O_4_ electrodes at 0.1 C rate for (**a**) initial and (**b**) fifth cycles. Reproduced from [156]. Copyright 2022 under the terms and conditions of the Creative Commons Attribution (CC-BY) license.

Numerous doping strategies have been investigated to limit manganese (Mn) dissolution and mitigate capacity loss. Partial substitution of Mn with transition metals—such as Ni, Mg, Al, Cr, Zn, Ti, Fe, and Cu—effectively reduces the concentration of Jahn–Teller active Mn^3+^ ions, thereby enhancing the structural stability and electrochemical performance of spinel-type cathodes [150,151]. Among these approaches, Ni substitution has attracted particular attention. In the LiMn_1.5_Ni_0.5_O_2_ compound, Ni incorporation increases the average oxidation state of Mn, suppressing Jahn–Teller distortion and stabilizing the spinel framework. This results in improved electrochemical properties, notably higher operating voltage, enhanced cycling stability, and superior rate capability [152]. Depending on synthesis conditions, Ni-substituted spinels can crystallize in either the disordered *Fd*$\bar{3}$*m* structure or the ordered *P*4_3_32 phase [153]. Furthermore, the reversible Ni^2+^/Ni^4+^ redox couple enables an operating voltage of approximately 4.7 V, paving the way for the development of high-voltage LiMn_1.5_Ni_0.5_O_4_ cathodes with significantly improved energy density [154]. However, several challenges remain, particularly the difficulty of obtaining stoichiometric LiNi_0.5_Mn_1.5_O_4_ without the formation of secondary nickel oxide impurities during synthesis [155]. Interestingly, the presence of a small amount of Mn^3+^ in the ordered structure can be beneficial, as it enhances electronic conductivity and facilitates charge compensation via oxygen deficiency. Experimentally, LiNi_0.5_Mn_1.5_O_4_ delivers a capacity of approximately 140 mAh g^−1^, a value slightly lower than its theoretical capacity of 147 mAh g^−1^. To further optimize electrochemical performance, surface modification strategies have also been extensively explored. Protecting coatings based on metal oxides such as ZnO, Al_2_O_3_, Co_3_O_4_, and MoO_3_ effectively stabilize the electrode/electrolyte interface, limit Mn dissolution, and improve cycling stability, as illustrated in Figure 18 [156].

*Critical assessment.* Doping and coating strategies applied to LiMn_2_O_4_ consistently improve capacity retention in half-cell configurations; however, manganese dissolution is a chemical process that persists even when the LMO electrode itself is stabilized, because dissolved Mn^2+^ ions migrate to the graphite anode (and the SEI interface) and degrade it in full cells [148,149]. Consequently, the cycling stability values reported in most of the studies summarized here—obtained in half-cells with a lithium metal anode—likely overestimate the durability that would be observed for an LMO/graphite battery under real-world operating conditions.

### 3.3. Polyanionic LiMPO_4_ Cathodes

#### 3.3.1. Historical Development and Commercialization

**Figure 19:**
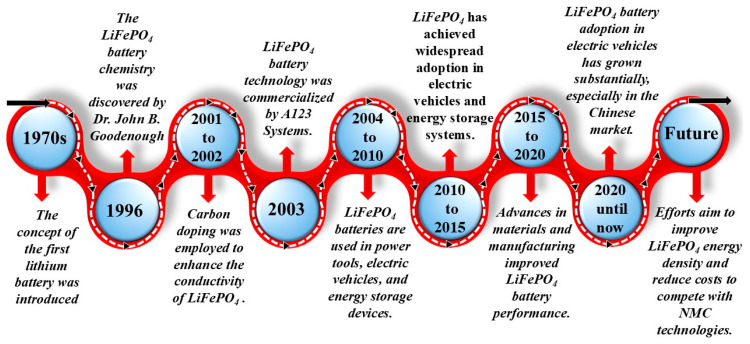
Timeline of LiFePO_4_ battery development from material to system.

Lithium iron phosphate (LiFePO_4_, LFP) has emerged as one of the most commercially successful cathode materials for LIBs, particularly in electric vehicles (EVs) and stationary energy-storage systems. As illustrated in Figure 19, the development of LFP spans more than four decades, beginning with the early conceptual advances in rechargeable lithium batteries during the 1970s and culminating in the mid-1990s with the identification, by Goodenough and co-workers, of olivine-structured LiFePO_4_ as a promising cathode material [157]. Despite its favorable electrochemical stability and safety, the practical implementation of LFP was initially limited by its intrinsically low electronic conductivity (~10^−9^ S cm^−1^), a value approximately six orders of magnitude lower than that of commercial LiCoO_2_ (10^−3^ S cm^−1^) [158]. This limitation was successfully addressed in the early 2000s through the development of carbon-coating strategies and carbon/active-material composites, which significantly improved charge transport and enabled high-rate capabilities and viable electrode performance [159,160].

The large-scale deployment of LFP technology has been driven by several key advantages, notably lower cost compared to layered oxide cathodes, superior thermal and chemical stability, excellent intrinsic safety, and long cycle life—often exceeding 2000 charge–discharge cycles [160,161]. Furthermore, the use of iron—an abundant and non-toxic resource—enhances the environmental sustainability and economic appeal of this chemistry. These characteristics enabled the early commercialization of LFP batteries by A123 Systems in the early 2000s, followed by extensive adoption in power tools, grid-scale energy storage, and, more recently, electric vehicles (Figure 19).

Since 2020, LFP technology has seen a strong resurgence of commercial interest in the electric vehicle sector, particularly in the Chinese market, driven by its intrinsic safety, cost competitiveness, and suitability for large-format prismatic cells used in “cell-to-pack” (CTP) integration strategies. The adoption of LFP chemistry for Tesla Model 3 and Model Y (since 2021), combined with the massive rollout of BYD’s “Blade” battery technology, has confirmed the commercial viability of this cathode chemistry for mass-market electric vehicles [162]. These developments have significantly accelerated global LFP production capacity and reinforced its position as a leading cathode material for energy-storage applications that prioritize cost control and high safety.

#### 3.3.2. Crystal Structure and Fundamental Limitations of LiFePO_4_

**Figure 20:**
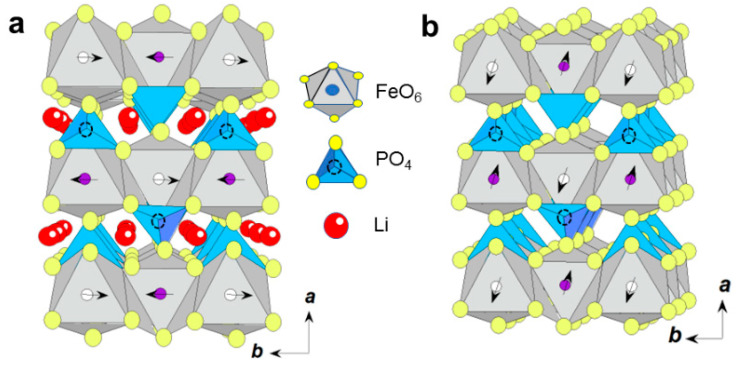
Crystal structure of (**a**) LiFePO_4_ and (**b**) FePO_4_ olivine framework. Corner-shared FeO_6_ octahedra are linked together in the *bc*-plane; LiO_6_ octahedra form edge-sharing chains along the *b*-axis. The tetrahedral PO_4_ groups bridge neighboring layers of FeO_6_ octahedra by sharing a common edge with one FeO_6_ octahedron and two edges with LiO_6_ octahedra.

The intrinsic safety and electrochemical stability of LFP stem from its particularly robust olivine-type crystal structure. LFP crystallizes in an orthorhombic olivine-type lattice belonging to the *Pnma* space group, characterized by a hexagonal close-packed arrangement of oxygen atoms. Within this structure, Li^+^ and Fe^2+^ ions occupy distinct octahedral sites, while P atoms are located in tetrahedral sites [163]. The resulting architecture consists of corner-sharing FeO_6_ octahedra interconnected by highly stable PO_4_^3−^ tetrahedra, thereby forming a rigid three-dimensional polyanionic network (Figure 20).

This unique polyanion framework underpins the characteristic properties of LFP. The very strong P–O covalent bonds firmly stabilize the oxygen sublattice, thereby preventing oxygen release and imparting exceptional thermal stability to the material. This significantly mitigates the risk of thermal runaway, representing a major safety advantage over oxygen-evolving layered oxides such as LiCoO_2_ [164]. At the same time, the olivine-type structure exhibits remarkable mechanical and structural robustness during the two-phase transition between LiFePO_4_ and FePO_4_, undergoing a unit-cell volume change of only ~6.9% upon complete lithium extraction/insertion [165]. This limited lattice strain is directly responsible for the outstanding cycling stability of LFP. Furthermore, this high structural stability gives rise to the characteristic flat electrochemical voltage plateau observed during lithiation/delithiation.

From the electrochemical standpoint, LFP delivers a theoretical specific capacity of approximately 170 mAh g^−1^ and operates via the Fe^2+^/Fe^3+^ redox reaction, featuring a stable voltage plateau near 3.45 V vs. Li^+^/Li [158,166]. This flat voltage response originates from the two-phase transformation between LiFePO_4_ and FePO_4_. Rapid migration of the phase interface facilitates efficient ionic and electronic conductivity during cycling. This mechanism contributes to LFP’s strong rate performance (capability for high currents) and excellent long-term cycle life, with capacity retention often exceeding 90% after 1000 cycles. Nevertheless, irreversible lithium loss at the phase interface remains a primary degradation mechanism, particularly during operation at high current densities.

#### 3.3.3. Fundamental Limitations

Despite its widespread commercial adoption, the olivine structure of LiFePO_4_ imposes several intrinsic limitations on its electrochemical performance. Notably, Li^+^ ion diffusion is confined to one-dimensional (1D) channels along the (010) crystallographic direction, resulting in relatively slow ion transport kinetics compared to the two-dimensional (2D) or three-dimensional (3D) diffusion pathways characteristic of layered or spinel cathode materials, respectively [167]. Furthermore, the strong inductive effect of the highly electronegative PO_4_^3−^ polyanion reduces orbital overlap between Fe 3d states and O 2p states, leading to extremely low intrinsic electronic conductivity. The combined effect of restricted ion diffusion and poor electron transport significantly limits the rate capability and high-power performance of pristine LiFePO_4_, rendering it unsuitable for fast charge–discharge applications without extensive material engineering. Another significant degradation mechanism is the irreversible loss of lithium at the phase boundary during repeated cycling, particularly at high current densities [168]. Consequently, substantial optimization strategies—such as particle nanosizing, conductive carbon coating, cation doping, and advanced electrode architectures—are generally required to achieve competitive electrochemical performance.

#### 3.3.4. Performance Enhancement Strategies for LiFePO_4_

The introduction of carbon-coating techniques in the early 2000s marked a major breakthrough in the development of LiFePO_4_ cathodes, substantially enhancing their intrinsically low electronic conductivity, while preserving structural integrity and cycling stability. Among the most effective approaches, graphene-coated porous LiFePO_4_ nanospheres demonstrated remarkable electrochemical performance, achieving a discharge capacity of 163.8 mAh g^−1^ at 0.1 C rate and retaining over 92% of their capacity after 500 cycles at 10 C [169]. Similarly, ultrathin (1–2 nm) highly graphitized carbon coatings enabled capacities of 143.6 mAh g^−1^ at 1 C with a capacity loss of only 1.47% after 50 cycles [170]. Typically, the carbon content is kept below 10 wt.% to optimize the trade-off between electronic conductivity and volumetric energy density, as excessive carbon loading lowers tap density and reduces overall energy storage efficiency [171].

Beyond conventional carbon coating, heteroatom doping strategies have emerged as an effective approach to further improve the electrochemical behavior of LiFePO_4_. In particular, doping with nitrogen and boron introduces additional defect sites and modifies the electronic structure of the carbon matrix, thereby increasing specific surface area, enhancing charge-transfer kinetics, and facilitating electron transport. Dual N,B-doped carbon-wrapped LiFePO_4_ composites have shown synergistic improvements in conductivity and electrochemical activity, attributed to an enhanced charge-carrier generation and more efficient lithium-ion diffusion pathways [172].

Recent advances in precursor engineering have leveraged metal–organic frameworks (MOFs) as self-sacrificial templates, enabling carbon modification, morphological control, and cation doping to be achieved simultaneously within a single synthetic step [173]. In. particular, precursors derived from Prussian blue analogues have been used to fabricate nitrogen-doped carbon-encapsulated LFP nanocomposites. This strategy generates an in situ nitrogen-doped carbon coating with a thickness of 3–5 nm, enhancing electronic conductivity and accelerating interfacial charge-transfer kinetics. Consequently, the material delivers outstanding electrochemical performance, including a reversible capacity of 153 mAh g^−1^ at 0.5 C over 500 cycles with 90.1% capacity retention, as well as excellent rate charge/discharge capability (120 mAh g^−1^ at 10 C) [174]. Building on this approach, oxygen- and fluorine-co-doped carbon-wrapped LFP composites were synthesized using iron-based MOFs (MIL-53(Fe)) as sacrificial templates. Through controlled pyrolysis under mixed atmospheres, simultaneous heteroatom doping was achieved, leading to enhanced carbon conductivity (~10^−2^ S cm^−1^) and improved electrolyte wettability. As a result, the composite demonstrated a discharge capacity of 160.9 mAh g^−1^ at 1 C after 500 cycles with 94.7% capacity retention, while maintaining 128 mAh g^−1^ at 5 C [175].

#### 3.3.5. Nanostructuring and Morphological Engineering

Morphological engineering through solvothermal synthesis in various reaction media enables precise control over LFP architectures, ranging from nanorods to complex hierarchical structures. For instance, rectangular prismatic nanorods synthesized in a water/glycerol medium delivered a discharge capacity of 163.8 mAh g^−1^ at 0.2 C, a performance attributed to optimized lithium-ion diffusion pathways along the [010] crystallographic direction [176]. These multiscale design strategies effectively reconcile the conflicting requirements of nano-engineering and micro-engineering. At the nanoscale, reducing particle dimensions shortens Li^+^ ion diffusion distances and increases the active surface area available for charge transfer, thereby enhancing rate capability. At the microscale, larger secondary structures increase tap density and volumetric energy density, while limiting excessive electrode–electrolyte interfacial area, which helps suppress parasitic side reactions. To balance these effects, hierarchical architectures have been developed; these consist of nanoscale primary particles (50–200 nm) assembled into microsized secondary particles (1–5 μm). Such structures make it possible to overcome the classic trade-off between high-rate performance and volumetric energy density [177]. However, despite superior electrochemical kinetics, nanostructured materials generally exhibit lower tap density (0.6–1.0 g cm^−3^) than commercial LFP materials (1.3–1.5 g cm^−3^), leading to reduced volumetric energy density—a major limitation for automotive battery applications.

#### 3.3.6. Synthesis Routes and Process Optimization

LFP can be synthesized using several techniques scalable to an industrial level, notably solid-state reactions, sol–gel process, hydrothermal/solvothermal synthesis, co-precipitation, and molten-state methods. Among these, the solid-state route remains the most widely adopted industrially due to its operational simplicity, the high crystallinity achieved, and excellent phase purity. However, this method generally requires high processing temperatures, resulting in significant energy consumption and, under an inert atmosphere, potential toxic gas emissions.

In recent years, hydrothermal and solvothermal techniques have gained considerable attention because they allow for precise control over particle size, morphology, and crystallinity at relatively low reaction temperatures (approximately 80–200 °C). These processes are usually followed by a post-annealing step between 500 and 750 °C to further improve crystal structure and electrochemical performance [178]. Similarly, it has been demonstrated that wet pre-lithiation coupled with carbothermal reduction at 500–600 °C yields highly homogeneous LFP powders with enhanced mass-transfer properties, delivering discharge capacities of approximately 148 mAh g^−1^ at 10 C [179]. Process optimization is crucial, as the synthesis temperature strongly influences phase composition and electrochemical behavior. Excessive firing temperatures above ~800 °C can lead to the formation of unwanted secondary phases such as Fe_2_P. Although Fe_2_P can improve electronic conductivity, its formation is generally associated with capacity loss and poor cycling stability [12]. Solution-based synthesis methods, including hydrothermal and solvothermal routes, offer superior control over particle morphology and size distribution; however, they often yield lower production rates (typically 60–75%) compared to conventional synthesis (>90%). These lower yields primarily due to losses during filtration, incomplete precipitation, and the increased complexity of liquid-phase processing.

#### 3.3.7. Cation Doping Strategies

Cation doping is another effective approach for modulating the phase transition mechanism and enhancing ionic conductivity, while largely preserving tap density. In this strategy, Li^+^ sites are partially substituted by aliovalent cations such as Mg^2+^, Al^3+^, Zr^4+^, Ti^4+^, Nb^5+^, typically at low concentrations (~1 at.%). These substitutions promote the formation of solid solutions and generate lattice defects; both phenomena can facilitate charge-carrier transport and improve the electrochemical performance of the material. Early studies reported dramatic increases in conductivity—spanning up to eight orders of magnitude—with values exceeding 10^−3^ S cm^−1^ [158]. However, subsequent research suggested that these enhancements might not stem solely from increased intrinsic conductivity within the material, but could also be strongly influenced by interfacial effects of the formation of a nano-network.

*Critical assessment.* LiFePO_4_ is one of the highest-performing cathode materials deployed on a commercial scale; however, its remarkable practical performance relies heavily on carbon coating and particle size reduction—measures intended to compensate for its intrinsically very low electronic conductivity (~10^−9^ S cm^−1^) [145]. Although these strategies have yielded excellent charge/discharge performance, they often require higher carbon content and the use of nanoparticles, which reduces the electrode’s tap density and, consequently, limits volumetric energy density [156,157]. Furthermore, improvements attributed to cation doping have, in several instances, been re-evaluated as stemming primarily from interfacial effects or nanoscale conductive networks, rather than from a genuine increase in ionic conductivity within the bulk material. Consequently, the electrochemical performance of LFP remains heavily dependent on electrode architecture and a rigorous interpretation of the underlying enhancement mechanisms.

### 3.4. High-Voltage Olivine Cathodes: Beyond LiFePO_4_

Although LiFePO_4_ has achieved widespread commercial adoption owing to its excellent thermal stability, long cycle life, and low cost [159], its relatively low operating potential (3.45 V vs. Li^+^/Li) inherently limits the achievable energy density in LIBs. Since the battery’s specific energy is approximately proportional to the product of capacity and voltage, increasing the operating voltage constitutes one of the most effective strategies for improving overall cell performance [180]. Within the Li*M*PO_4_ olivine family (*M* = Fe, Mn, Co, Ni), the partial or total substitution of iron by alternative transition metals provides a promising route toward higher-voltage cathode chemistries, while preserving the intrinsic structural robustness and thermal safety associated with the olivine structure [181]. In principle, these materials retain the characteristic one-dimensional lithium diffusion channels and strong covalent phosphate framework that characterize LFP and contribute to its remarkable stability. In polyanion cathodes such as Li*M*PO_4_, the redox potential of the transition-metal center is strongly governed by the inductive effect of the phosphate group (PO_4_^3−^). The highly electronegative phosphate polyanion draws electron density away from the metal–oxygen bonds, thereby reducing the covalency of these bonds and stabilizing the antibonding states involved in the redox process. As a result, the operating voltage generally increases with the electronegativity of the transition-metal species [182]. However, the correlation between transition-metal electronegativity and electrochemical potential is not purely linear; indeed, crystal-field effects, electronic configuration, Jahn–Teller distortions, and metal–oxygen hybridization also play a decisive role in determining the nature of accessible redox couples and their respective voltages.

Although iron exhibits moderate electronegativity (χ = 1.83 on the Pauling scale), the Fe^2+^/Fe^3+^ redox couple in LiFePO_4_ displays a lower-than-expected potential (3.45 V). This behavior is explained by the electronic structure of high-spin Fe^2+^ (3*d*^6^): oxidation to Fe^3+^ (3*d*^5^) requires the removal of an electron from a doubly occupied *t*_2_g orbital, thereby entailing an additional energy cost associated with electron pairing energy [183]. In contrast, the Mn^2+^ ion adopts a high-spin half-filled 3*d*^5^ configuration, in which the five d-electrons remain unpaired. Oxidation to Mn^3+^ (3*d*^4^) therefore occurs without an additional pairing-related energy cost. Combined with the relatively low crystal-field stabilization energy of the high-spin *d*^5^ state, this phenomenon leads to a thermodynamically more favorable oxidation process and, consequently, a higher Mn^2+^/Mn^3+^ redox potential (~4.1 V) in LiMnPO_4_ [184]. These electronic-structure effects form the thermodynamic basis for developing higher-voltage olivine-type cathodes by substituting Fe with alternative transition metals, such as Mn.

#### 3.4.1. Lithium Manganese Phosphate (LiMnPO_4_)

Lithium manganese phosphate is a promising member of the olivine-structured cathode family. Sharing the same olivine-type crystal structure as LiFePO_4_, LiMnPO_4_ substitutes manganese for iron, imparting significantly different electrochemical characteristics to the material. It operates at a higher voltage—approximately 4.1 V vs. Li^+^/Li—driven by the Mn^2+^/Mn^3+^ redox couple, corresponding to an increase of nearly 18% compared to LiFePO_4_ [185]. This higher operating voltage results in a substantially improved theoretical energy density of approximately 701 Wh kg^−1^, surpassing the value ~586 Wh kg^−1^ typically reported for LiFePO_4_ [186]. Despite this higher energy density, LiMnPO_4_ retains a theoretical specific capacity of approximately 171 mAh g^−1^, comparable to that of other olivine phosphate cathodes. Beyond its enhanced energy performance, LiMnPO_4_ preserves the intrinsic safety advantages associated with the olivine framework. The strong P–O covalent bond contributes to excellent thermal stability and high resistance to oxygen release and thermal runaway, making the material particularly attractive for safe LIB applications [156].

**Figure 21:**
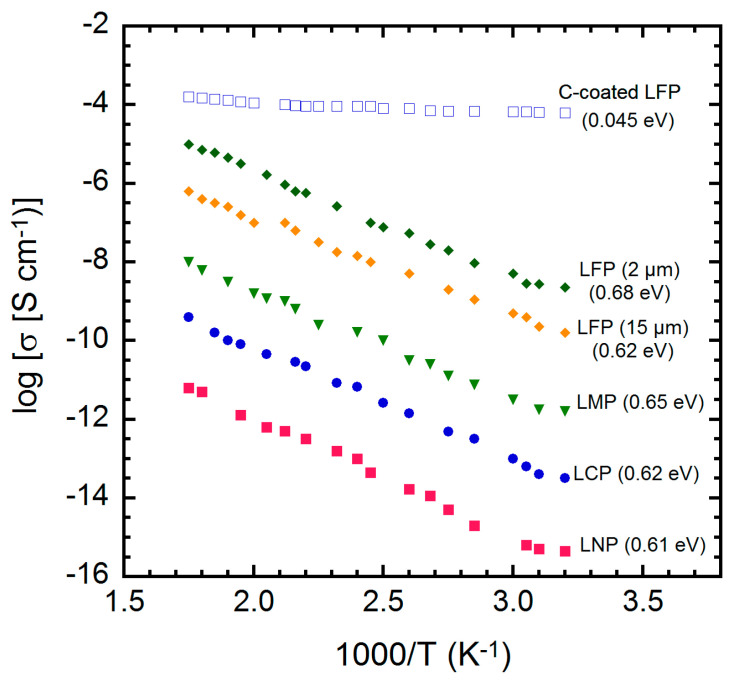
Electrical conductivity of Li*M*PO_4_ (*M* = Fe, Ni, Co, Mn) olivine materials. The numbers indicate the activation energy in eV. The low conductivity is related to the small free volume and separation of *M*O_6_ octahedra by oxygen atoms of the (PO_4_)^3−^ anions. Reproduced from [12]. Copyright 2016 Springer.

Despite these appealing properties, LiMnPO_4_ still faces major hurdles to commercialization. Like other olivine phosphates, it possesses an intrinsically wide bandgap (>3 eV), which leads to extremely low electronic and ionic conductivity; these values are far below the levels required for practical battery operation and notably poorer than those of LiFePO_4_ [186,187]. Figure 21 shows that these transport limitations severely hinder the achievement of high capacities at sustained discharge rates, even when employing carbon-coating strategies similar to those successfully applied to LFP [160]. This difficulty is exacerbated by the one-dimensional diffusion pathway of Li^+^ ions along the [010] crystallographic direction, which creates structural bottlenecks in the kinetics. Furthermore, antisite defects (Li/*M* exchange) additionally impede Li^+^ transport, ultimately limiting rate capability and hindering the widespread practical deployment of LiMnPO_4_ [157,188].

LiMnPO_4_ also exhibits a specific structural limitation linked to the Jahn–Teller activity of Mn^3+^ ions (high-spin *d*^4^, *t*_2g_^3^*e*_g_^1^ configuration) arising from the degeneracy of the *e*_g_ orbitals [189]. Upon delithiation, this electronic instability induces a cooperative tetragonal distortion of the MnO_6_ octahedra, leading to pronounced lattice instability, anisotropic strain, and contraction along the c-axis—all factors that hinder Li^+^ ion transport [190]. These structural distortions promote mechanical degradation, resulting in rapid capacity fading and poor high-rate performance, thereby compromising both high-rate capability and long-term cycling stability. Consequently, carbon-coating strategies that successfully mitigated the comparatively milder phase-boundary limitations in LiFePO_4_ have proven insufficient to overcome the far more severe kinetic constraints inherent to LMP [188].

#### 3.4.2. Enhancement Strategies

Numerous strategies have been developed to overcome the intrinsic limitations of LiMnPO_4_, many of which draw upon optimization methods proven effective for LiFePO_4_. Among these, carbon coating remains the most widely used fundamental method, as conformal carbon layers can significantly enhance surface electronic conductivity. The nature of the carbon precursor plays a decisive role in the structure, conductivity, and overall electrochemical performance of the resulting nanocomposite. For instance, Li et al. [191] reported that the rod-shaped LMP/C composites synthesized using beta-cyclodextrin as the carbon source exhibited a high reversible capacity of 153 mAh g^−1^ at C/10, clearly outperforming analogous materials prepared with more precursors such as glucose, sucrose, or citric acid. Concurrently, solid-state routes combined with various carbon sources have also been extensively explored to create efficient conductive carbon networks [192,193]. In these systems, optimizing the coating, homogeneity, and degree of graphitization is essential to maximize electrochemical performance and ensure effective charge transport [194]. More advanced synthetic approaches, such as microwave-assisted solvothermal synthesis, have yielded particularly promising results. Certain LMP/C nanocomposites prepared using these techniques demonstrated capacities reaching 155 mAh g^−1^ at 0.5 C after 100 cycles and maintaining 118 mAh g^−1^ at a demanding 10 C rate [195].

Reducing particle size through nanostructuring is another key strategy for improving the electrochemical performance of LiMnPO_4_, as it shortens Li^+^ ion diffusion pathways and increases the electrode–electrolyte interfacial area. Drezen et al. [196] demonstrated that crystallite size can be precisely controlled by adjusting the sintering temperature during precursor-based synthesis, thereby establishing a direct correlation between processing conditions, particle morphology, and electrochemical behavior. However, excessive downsizing must be managed carefully, as it risks lowering tap density and exacerbating parasitic side reactions with the electrolyte [197]. Among compositional modification approaches, metal doping—particularly with iron—has shown considerable potential. Fe substitution in LiMnPO_4_ effectively alleviates the Jahn–Teller distortion, while simultaneously enhancing both ionic and electronic conductivities. The resulting LiMn_1−x_Fe_x_PO_4_ solid solutions combine the high operating voltage associated with manganese with the superior transport properties of iron-based olivine frameworks, thereby offering a favorable compromise between energy density and kinetics [198]. Co-doping strategies have also attracted significant interest. Kim et al. [199] demonstrated that the simultaneous incorporation of small amounts of Fe and Co into the LMP structure markedly improved electrochemical performance while preserving the characteristic ~4.0 V redox potential. This enhancement was attributed to the formation of a local solid-solution-like environment within the olivine framework, which lowers the nucleation barrier of the delithiated phase and alleviates structural strain associated with the Jahn–Teller effect. In addition to iron and cobalt, other dopants such as vanadium [200], lanthanum [201], and zirconium [202] have also been investigated for their ability to stabilize the crystal structure and further enhance electrochemical performance.

A wide range of synthesis strategies have been developed to tailor the morphology, crystallinity, and surface characteristics of LiMnPO_4_ in order to improve its electrochemical performance. Among these, hydrothermal and solvothermal methods are particularly noteworthy as they offer excellent control over stoichiometry, crystal growth, particle size, and phase purity [203,204,205]. These approaches yield highly crystalline and uniformly distributed particles, thereby beneficial for enhancing Li^+^ ion diffusion and electrode kinetics. Ionothermal synthesis employing ionic liquid media has also emerged as an effective route for morphology engineering, allowing the formation of diverse nanostructures such as nanorods, nanoplates, and spindle-shaped particles [206,207,208]. Similarly, approaches based on deep eutectic solvent have gained attention as environmentally friendly alternatives; they offer comparable control over particle morphology while adhering to green-chemistry principles [195]. Precipitation-based synthesis routes using precursors such as NH_4_MnPO_4_·H_2_O and MnPO_4_·H_2_O allow for precise control over stoichiometry and precursor homogeneity—two crucial factors for obtaining phase-pure LiMnPO_4_ with improved electrochemical performance [38,192,196,209]. Concurrently, spray pyrolysis combined with wet ball milling has been successfully utilized to fabricate LiMg_x_Mn_1−x_PO_4_/C composite cathodes, offering an effective approach for producing carbon-coated, compositionally modified olivine materials with enhanced structural and electrochemical properties [210,211].

### 3.5. Other High-Voltage Olivine Cathodes

**Figure 22:**
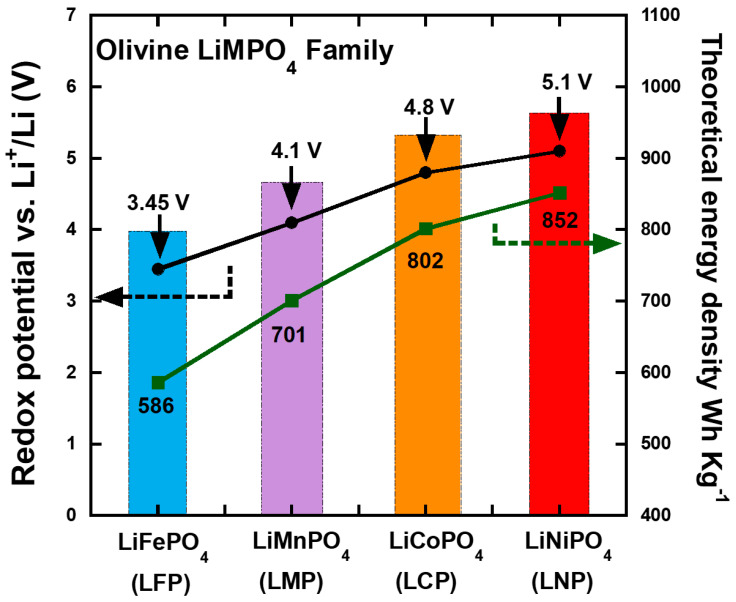
Redox potential and theoretical energy density of olivine Li*M*PO_4_ cathode materials (*M* = Fe, Mn, Co, Ni).

Figure 22 compares the operating voltages and theoretical energy densities within the Li*M*PO_4_ olivine family. The electronic structure effects—responsible for elevating the Mn^3+^/Mn^2+^ redox potential relative to the Fe^3+^/Fe^2+^ couple—are even more pronounced for cobalt- and nickel-based olivine materials. Consequently, LiCoPO_4_ (LCP) operates at approximately 4.8 V vs. Li^+^/Li driven by the Co^2+^/Co^3+^ redox reaction, while LiNiPO_4_ (LNP) reaches an even higher operating voltage around 5.1 V via the Ni^3+^/Ni^2+^ redox couple. These voltages represent increases of roughly 39% and 48%, respectively, compared to LiFePO_4_, leading to remarkable theoretical energy densities of nearly 802 Wh kg^−1^ for LCP and ~852 Wh kg^−1^ for LNP [206,207]. Despite these highly attractive energy characteristics, the substantial increase in operating voltage entails considerably greater technical and electrochemical challenges, which have thus far limited the practical implementation of these high-voltage olivine cathodes.

For both LiCoPO_4_ and LiNiPO_4_, very high operating voltages (>4.5 V) give rise to major compatibility issues with conventional carbonate-based electrolytes. At such potentials, electrolyte oxidation becomes thermodynamically favorable, leading to the formation of resistive solid–electrolyte interphase (CEI/SEI) layers, gas evolution (e.g., CO_2_ and alkyl carbonate species), and continuous parasitic reactions that progressively accelerate capacity loss [212,213]. Consequently, considerable effort has been devoted to mitigating these interfacial instabilities through surface engineering and electrolyte optimization. In particular, protective coatings such as AlPO_4_ and FePO_4_ have been employed to suppress direct electrode–electrolyte reactions, while alternative electrolyte systems—including ionic liquids, fluorinated carbonates, or solid-state electrolytes—are being actively explored to improve high-voltage stability [201,202,203,204,205,206,207,208,209,210,211,212,213,214,215,216,217].

Among these materials, the synthesis of LiNiPO_4_ in a single-phase form remains especially challenging. Conventional high-temperature solid-state routes frequently generate impurity phases such as Li_4_P_2_O_7_ (lithium pyrophosphate) and Ni_3_P (nickel phosphide), primarily due to the thermodynamic instability of LiNiPO_4_ and the volatilization of phosphorus above 600 °C [218]. These secondary phases not only decrease the fraction of electrochemically active material but also increase interfacial impedance and promote undesirable side reactions. Although low-temperature hydrothermal synthesis methods help limit impurity formation, the resulting products often exhibit limited crystallinity and very small particle sizes, which adversely affects tap density and packing efficiency within the electrode [177].

*Critical assessment.* Although LMFP is widely considered to combine the high energy density of LiMnPO_4_ with the excellent cycling stability of LiFePO_4_, the reported performance improvements remain highly composition-dependent. While energy density gains of around 20% compared to LFP have been demonstrated, they are often accompanied by reduced cycling stability relative to pure LFP [218,219]. Furthermore, the optimal Mn:Fe ratio varies significantly across studies, indicating that no universally optimal composition has yet been established. Consequently, LMFP compositions should be tailored to specific application requirements—balancing energy density, rate capability, and service life—rather than seeking a single optimal formulation.

Despite having operating voltages and theoretical energy densities superior to those of LiFePO_4_, LiMnPO_4_, LiCoPO_4_, and LiNiPO_4_ materials exhibit capacities that remain well below their theoretical limits due to persistent electrolyte oxidation, poor interfacial stability, and difficulties in achieving high phase purity [212,213]. The persistence of these limitations after nearly two decades of research suggests that further progress is unlikely to be achieved solely through cathode modification. Instead, unlocking the full potential of these high-voltage polyanionic cathodes will require parallel advances in electrolyte design, particularly regarding solid-state and high-voltage-stable electrolyte systems.

### 3.6. Lithium Manganese Iron Phosphate (LMFP) Solid Solutions

Given the complementary strengths and limitations of LiMnPO_4_ and LiFePO_4_, the LiMn_x_Fe_1−x_PO_4_ (LMFP) solid-solution system has emerged as one of the most promising strategies for developing high-voltage olivine-type cathodes. LMFP combines the higher operating voltage of the Mn^2+^/Mn^3+^ redox couple with the superior electrochemical kinetics, electronic conductivity, and structural stability characteristic of iron-containing olivines. Partial substitution with Fe mitigates Mn^3+^-induced Jahn–Teller distortion, enhances both ionic and electronic transport, and lowers the kinetic barrier associated with the two-phase transition process [198,199].

Representative compositions such as LiMn_0.6_Fe_0.4_PO_4_ and LiFe_0.9_Mn_0.1_PO_4_ have demonstrated discharge capacities approaching 160 mAh g^−1^ at moderate current densities, with average operating voltages in the range 3.85–4.0 V and gravimetric energy densities reaching approximately 559 Wh kg^−1^. These values correspond to an energy-density improvement of nearly 20% compared to commercial LFP, while preserving the intrinsic thermal stability and safety advantages of olivine-structured phosphates [219,220]. In situ synchrotron diffraction investigations of LiFe_0.5_Mn_0.5_PO_4_ during electrochemical cycling have provided important mechanistic insights into the role of iron in modifying structural evolution and phase transition dynamics [221]. It has been shown that iron incorporation limits lattice strain accumulation, facilitates phase-boundary migration, and promotes more homogeneous lithiation/delithiation behavior, thereby improving rate capability and cycling stability relative to pure LiMnPO_4_. Although pure LiMnPO_4_ remains primarily confined to laboratory-scale research because of its intrinsically low conductivity and the sluggish kinetics of the Mn^3+^/Mn^2+^ reaction, LMFP solid solutions are increasingly attracting commercial interest. Several battery manufacturers have initiated pilot-scale production, and next-generation high-voltage LMFP cathodes are expected to occupy a growing share of the lithium-ion battery market in the coming years. Meanwhile, emerging recycling and upcycling approaches have demonstrated the direct conversion of mixed spent LiFePO_4_ and LiMn_2_O_4_ cathodes into high-performance LMFP materials, thereby simultaneously addressing sustainability challenges and electrochemical performance requirements [212].

### 3.7. Other Polyanionic Cathode Families Beyond the Olivine Structure

The landscape of polyanion-based cathodes extends well beyond the LiMPO_4_ olivine family discussed in Section 3.3. Although none of the following material classes has yet achieved the commercial maturity of LiFePO_4_, several exhibit interesting electrochemical characteristics that have sparked keen interest among researchers and broadened the scope of possibilities for the design of next-generation polyanionic cathodes.

*NASICON-type phosphates*. Li_3_V_2_(PO_4_)_3_ (LVP), with either a monoclinic or rhombohedral structure, features a three-dimensional (3D) framework akin to that of NASICON, containing interconnected diffusion channels that facilitate rapid Li^+^ ion transport [222]. In principle, up to three Li+ ions per formula unit can be extracted via the V^3+^/V^4+^ (≈3.6 V) and V^4+^/V^5+^ (≈4.6 V) redox couples, corresponding to a theoretical capacity of approximately 197 mAh g^−1^. However, the extraction of the third Li^+^ ion is accompanied by a significant structural rearrangement that limits practical reversibility. Consequently, actual capacities are generally limited to the 130–160 mAh g^−1^ range, while the relatively high cost and environmental concerns associated with vanadium further hinder its large-scale deployment [222].

*Vanadyl phosphates.* VOPO_4_ (in its various polymorphic forms αI-, αII-, β-, and ε-) and LiVOPO_4_ leverage multi-electron vanadium redox reactions and offer a theoretical capacity exceeding 300 mAh g^−1^ when more than one Li^+^ per formula unit participates in the electrochemical reaction [223]. However, their practical performance remains well below this theoretical potential as extensive lithiation or delithiation induces structural instability specific to each polymorph, while their relatively low electronic conductivity further limits rate performance (rate capability).

*Fluorophosphates.* Incorporating fluorine into the framework strengthens the inductive effect on the transition-metal redox couple, thereby raising the operating voltage compared to the corresponding fluorine-free phosphate. LiVPO_4_F operates at ≈4.2 V with a theoretical capacity of ≈155 mAh g^−1^ [224], while tavorite-related Li_2_FePO_4_F [225] and Li_2_MnPO_4_F represent attractive Fe- and Mn-based alternatives. Compared to conventional phosphates, fluorophosphates generally offer higher operating voltages, while maintaining good thermal stability. However, obtaining phase-pure materials often requires more complex synthesis processes, which currently limits reproducibility and large-scale production.

*Silicates.* In principle, Li_2_FeSiO_4_ and Li_2_MnSiO_4_ are capable of exchanging more than one Li^+^ per formula unit, thereby offering theoretical capacities approaching 330 mAh g^−1^ (approximately double that of LiFePO_4_); this is because the SiO_4_^4−^ polyanion can accommodate the higher formal charge associated with two-electron redox reactions of the transition metals [226]. In practice, however, extraction of the second Li^+^ ion requires substantial structural reorganization, while the low intrinsic electronic and ionic conductivity of silicate structures limits the actually accessible capacities to values well below the theoretical capacity, even when employing extensive carbon coating and nanostructuring. Consequently, overcoming these coupled structural and transport-related limitations remains the major challenge for the practical development of silicate-based cathodes.

*Sulfates.* Tavorite-type LiFeSO_4_F exploits the strong inductive effect of the sulfate group to raise the potential of the Fe^3+^/Fe^2+^ redox couple to approximately 3.6 V—a value significantly higher than in LiFePO_4_—while retaining the low cost and environmental benignity of iron-based chemistry [227]. However, the limited thermal stability of the sulfate precursors necessitates low-temperature ionothermal synthesis, which increases processing complexity and limits both practical capacity (typically 120–140 mAh g^−1^) and large-scale reproducibility.

*Borates*. LiFeBO_3_ contains the lightest possible polyanion group (BO_3_^3−^), giving it the highest theoretical capacity (≈220 mAh g^−1^) among Fe-based polyanionic cathodes [228]. This advantage is offset by very poor intrinsic electronic conductivity and a relatively narrow synthesis window; these two factors have restricted the study of borates to early-stage laboratory research rather than practical development.

Overall, these additional polyanion families illustrate that the design based on the inductive effect—which underpins olivine-type phosphates (Section 3.4)—can be successfully extended to a much wider range of crystal structures. Despite their structural diversity, these materials share remarkably similar challenges, notably intrinsically low electronic conductivity, sluggish Li^+^ transport kinetics, and increasing electrolyte instability at elevated operating voltages. Consequently, their limited commercial adoption reflects not a lack of theoretical energy-storage capability, but rather the difficulty of reconciling high conductivity, structural stability, and electrolyte compatibility under practical operating conditions.

*Critical assessment.* Although NASICON-type phosphates, fluorophosphates, silicates, sulfates, borates, and vanadyl phosphates offer attractive theoretical capacities or operating voltages, none has yet achieved the commercial success of LiFePO_4_. Most reported performance improvements rely on nanostructuring, carbon coating, or increasingly complex synthesis strategies aimed at compensating for intrinsically poor electronic conductivity and limited electrolyte compatibility. Furthermore, the electrochemical performance of these materials remains highly sensitive to phase purity, electrode architecture, and testing conditions, making direct comparisons between different studies difficult. Thus, future progress will likely not stem solely from crystal structure engineering, but will require the simultaneous optimization of cathode chemistry, electrode architecture, industrially scalable synthesis processes, and high-voltage-compatible electrolyte systems.

### 3.8. Cross-Family Comparative Perspective and Future Outlook

The continuous evolution of Li-ion battery cathodes, discussed in Section 3.1, Section 3.2, Section 3.3, Section 3.4, Section 3.5, Section 3.6 and Section 3.7, clearly demonstrates that no single chemical composition simultaneously meets all the requirements for next-generation energy storage. Instead, each cathode family occupies a distinct position within the multidimensional trade-off space defined by specific energy, power, thermal stability and cycling performance, cost, and manufacturing process maturity. Consequently, the choice of cathode material must be guided primarily by the specific requirements of the intended application, rather than by the maximization of any single electrochemical parameter in isolation.

Layered oxides (Section 3.1.1, Section 3.1.2, Section 3.1.3, Section 3.1.4, Section 3.1.5, Section 3.1.6, Section 3.1.7 and Section 3.1.8) remain the dominant high-energy applications because of their high operating voltage and specific capacities. Nevertheless, their widespread practical implementation remains constrained by Li^+^/transition-metal cation disorder [23,36,37,38], oxygen evolution, structural degradation at high states of charge [83,84,85,86,87], and progressively reduced thermal stability with increasing Ni content [85,86,87,88,89]. Although bulk doping, surface coatings, concentration gradient architectures, and single-crystal particle design (Section 3.1.7 [140,141,142,143]) each mitigate specific degradation mechanisms, none provides a universal solution. Instead, the highest electrochemical performance is consistently achieved through the synergistic integration of multiple modification strategies.

Conversely, spinel LiMn_2_O_4_ (Section 3.2) delivers excellent performance under rapid charge/discharge conditions, facilitated by a three-dimensional Li^+^ ion diffusion network; it remains an attractive option due to its relatively low cost and cobalt-free composition. Nevertheless, manganese dissolution and Jahn–Teller distortion continue to limit long-term cycling stability [148,149,151]. Meanwhile, olivine-type cathodes and other polyanionic structures (Section 3.3, Section 3.4, Section 3.5, Section 3.6 and Section 3.7) remain the benchmark for thermal stability, safety, and cycle life [157,160,161]. However, their widespread adoption remains hindered by intrinsically low electronic conductivity and—for most emerging polyanion families other than olivines—by practical validation that is still limited to laboratory studies [222,223,224,225,226,227,228].

A recurring observation across all cathode families is that many of the electrochemical improvements reported in the literature are achieved under optimized laboratory conditions, including half-cell measurements, low electrode loading, moderate current density, and relatively short cycling protocols. Furthermore, differences in voltage windows, electrolyte formulations, electrode preparation, and testing protocols often account for a significant portion of the discrepancies observed between nominally similar materials (see the critical assessment paragraphs in each subsection above). Consequently, a direct comparison of absolute electrochemical performance between independent studies should only be undertaken when comparable experimental protocols are used.

Another major trend emerging from this study is the shift from composition-focused optimization to multiscale structural engineering. Rather than relying on a single modification strategy, current cathode development increasingly integrates—within unified design frameworks—bulk doping, particle morphology control, surface or interface coatings, the design of particles with internal concentration gradients or single-crystal structures, and advanced “operando” characterization techniques (Section 5.1). This evolution reflects a broader shift from empirical material development to the engineering of structure–property relationships guided by an understanding of underlying mechanisms.

Looking ahead, progress will likely depend less on the discovery of entirely new compounds than on the convergence of industrially scalable synthesis methods (Section 4), interface engineering compatible with solid-state electrolytes [229,230,231], and sustainable recycling and direct regeneration technologies [232,233,234,235]. At the same time, the continued exploitation of anionic redox chemistry mechanisms—in both lithium-rich layered oxides (Section 3.1.6 [62,63,64,65]) and disordered rock-salt cathodes (Section 3.1.8 [144,145])—is expected to play a key role in overcoming the energy density limitations of conventional transition-metal-redox-based cathodes. Ultimately, the future success of Li-ion battery cathodes will depend not only on maximizing electrochemical performance but also on the ability to reconcile long-term stability, large-scale production, economic viability, and environmental sustainability.

## 4. Synthesis of Nanostructured Cathode Materials

**Figure 23:**
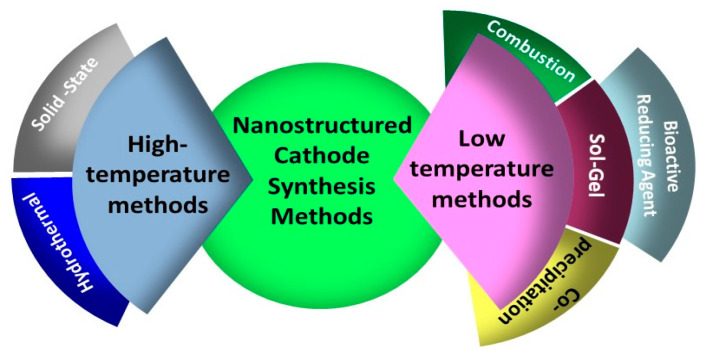
Methods for the synthesis of nanostructured cathode materials.

The morphological characteristics of cathode materials—specifically structure, particle size, grain size distribution, surface area, and crystallinity—are strongly influenced by the synthesis method employed. These parameters directly affect lithium-ion diffusion pathways during electrochemical processes, thereby profoundly influencing the battery’s overall performance. Consequently, the electrochemical behavior of cathode materials can vary significantly depending on the synthesis route adopted. One of the most promising approaches to enhancing the performance of nanostructured electrode materials is nanostructuring itself [236]. Reducing particle dimensions shortens lithium-ion diffusion distances, increases electrode–electrolyte contact area, and improves reaction kinetics—all of which contribute to higher capacity and superior rate capability. However, the fragility of nanoscale lattice structures must also be considered during synthesis, as the structural properties of cathode materials are highly sensitive to processing conditions. Based on synthesis temperature, preparation methods can generally be classified into high-temperature and low-temperature approaches, as illustrated in Figure 23.

- *High-temperature synthesis*: The solid-state reaction (SSR) method remains the most widely employed high-temperature synthesis technique because of its simplicity and suitability for large-scale industrial production. However, it often produces relatively large particles and offers limited control over particle morphology.
- *Low-temperature synthesis*: Low-temperature methods are generally more sophisticated and provide improved control over particle size and morphology, enabling the preparation of highly pure and homogeneous phases. These approaches typically utilize organic additives or chelating agents—such as citric acid, ethylene glycol, polyvinyl alcohol, and EDTA—to construct the lattice framework at the molecular level, thereby enhancing crystallinity and compositional uniformity.

Thus, the choice of synthesis route plays a decisive role in tailoring the structural, morphological, and electrochemical properties of cathode materials. In particular, nanostructuring and wet-chemistry approaches have emerged as highly attractive strategies for next-generation lithium-ion batteries, owing to their ability to enhance lithium-ion diffusion kinetics, improve electronic conductivity, and provide precise control over particle size and compositional homogeneity. These synthesis methods not only enable superior electrochemical performance but also facilitate the development of high-energy-density cathodes with improved cycling stability and rate capability.

### 4.1. Fundamentals of Nanostructuring Effects on Electrochemical Performance

Nanostructuring modifies the cathode performance of lithium-ion battery cathodes through several interconnected mechanisms operating at the particle, interface, and lattice-defect levels. Although the magnitude of these effects varies with crystal structure and intrinsic transport properties, the same fundamental principles apply across layered, spinel, olivine, and other polyanionic cathodes, explaining why nanoscale engineering has become a common strategy throughout the cathode families discussed in this review.

*Particle size effects.* In most intercalation cathodes, Li^+^ transport within an individual particle is diffusion-controlled, and the characteristic diffusion time scales approximately with the square of the diffusion length (τ ≈ *L*^2^/*D*, where *D* is the chemical diffusion coefficient of Li^+^ in the host lattice) [187]. Consequently, reducing the primary particle size from the micrometer to the nanometer scale shortens the Li^+^ diffusion pathway and substantially improves lithiation/delithiation kinetics. This effect is particularly important for cathodes with intrinsically low Li^+^ diffusivity and electronic conductivity, such as LiFePO_4_ and LiMnPO_4_ [158,187], where nanosizing is essential for achieving practical high-rate performance (Section 3.3 and Section 3.4). However, decreasing particle size simultaneously increases the electrode–electrolyte contact area, improving charge-transfer kinetics while also increasing susceptibility to parasitic surface reactions.

*Interface and surface engineering.* As particle size decreases, the fraction of atoms residing at or near the surface increases substantially, making surface reactivity an increasingly dominant factor in overall electrochemical behavior. Protective surface coatings, including metal oxides, metal fluorides, phosphates, and conductive carbs, perform several complementary functions: (i) suppressing electrolyte decomposition and transition-metal dissolution by physically isolating the active material from the electrolyte [90,91,92,93,94,95,96,97]; (ii) mitigating surface reconstruction into electrochemically inactive rock-salt- or spinel-like phases during repeated cycling [83,84,85,86,87]; and (iii) maintaining or even enhancing interfacial Li^+^ ion transport across the electrode–electrolyte interface when ionically conductive coatings are employed. Because nanoparticles possess a much larger surface-to-volume ratio than micron-size particles, the effectiveness of thin protective coatings becomes significantly more pronounced in nanostructured cathodes.

*Defect engineering.* Nanoscale synthesis and low-temperature processing strongly influence the formation of lattice defects, including Li^+^/transition-metal antisite disorder, oxygen vacancies, and stacking faults. Whether these defects improve or degrade electrochemical performance depends on their type, concentration, and spatial distribution. For example, Li^+^/Ni^2+^ cation mixing disrupts layered ordering and impedes Li^+^ diffusion in Ni-rich layered oxides [23,36,37,38]. Whereas a controlled degree of oxygen deficiency in ordered LiNi_0.5_Mn_1.5_O_4_ spinel enhances electronic conductivity through mixed Mn^3+^/Mn^4+^ valence states [154,155]. Consequently, nanostructuring alone is insufficient to optimize cathode performance and must be combined with appropriate defect-control strategies such as cation doping, controlled-atmosphere calcination, or post-synthesis annealing.

*Surface chemistry and the nanostructuring trade-off.* Although nanosizing generally improves reaction kinetics, it also increases the specific surface area available for oxygen evolution, transition-metal dissolution, electrolyte decomposition, and cathode–electrolyte interphase (CEI) formation, particularly under high voltage or elevated temperature. Furthermore, nanostructured powders generally exhibit lower tap density than micron-sized materials, reducing volumetric energy density and complicating electrode processing. Consequently, successful nanostructured electrode fabrication requires careful optimization of particle size and morphology rather than indiscriminate particle size reduction. This design philosophy is reflected in hierarchical nano-in-micro architectures, concentration gradient particles, and single-crystal cathodes, which combine rapid nanoscale Li^+^ ion transport with the mechanical robustness, packing density, and long-term stability of larger secondary particles.

*Critical Perspective.* Although nanostructuring is one of the most effective approaches for improving lithium-ion transport and rate capability, it should not be regarded as a universally applicable solution. Excessive particle size reduction inevitably increases surface reactivity, accelerates interfacial degradation, lowers tap density, and may reduce practical cell-level energy density despite improvements in intrinsic material kinetics. Consequently, the optimum particle size is highly material-dependent and reflects a balance among transport kinetics, structural stability, electrode architecture, and manufacturing practicality rather than the smallest achievable particle dimensions. Modern cathode design therefore increasingly integrates nanoscale engineering with compositional optimization, interface stabilization, and scalable synthesis to maximize practical electrochemical performance.

### 4.2. Solid-State Reaction (SSR)

The solid-state reaction (SSR) method is one of the most classic and widely used techniques for synthesizing powdered materials from solid precursors. This method relies on high temperature and is valued for its simplicity, ease of implementation, low cost, and minimal equipment requirements. In SSR synthesis, the physicochemical properties of the raw materials—such as reactivity, specific surface area, and free energy—as well as external parameters (temperature, pressure, and reaction atmosphere) strongly influence the characteristics of the final product. Using the SSR approach, Jiang et al. [237] successfully prepared a layered cathode material of the Li(Ni_1/3_Co_1/3_Mn_1/3_)O_2_ type. The resulting compound exhibited a low degree of cation mixing and delivered a specific discharge capacity of 218.9 mAh g^−1^ with a Coulombic efficiency of 99.41% within the voltage range of 2.8–4.3 V (vs. Li^+^/Li). These results demonstrate the capability of the SSR method to produce layered cathode materials with promising electrochemical performance. Despite these advantages, SSR also presents several limitations compared to solution-based synthesis techniques. Although the method is facile, scalable to an industrial level, and compatible with mass production, it often yields materials characterized by heterogeneous morphology, large particle sizes, and broad particle size distribution. Furthermore, the prolonged high-temperature calcination process, combined with repeated grinding, mixing, and annealing steps, can lead to particle coarsening and oxygen deficiency. These drawbacks can promote the formation of impurity phases and, consequently, compromise the structural integrity and electrochemical performance of the resulting cathode materials.

### 4.3. Hydrothermal Methods (HTMs)

The hydrothermal method (HTM) is one of the most widely employed synthesis techniques for preparing metal oxide nanostructures intended for cathodes, owing to its ability to produce materials with high crystallinity, fine particle size, uniform distribution, and limited particle agglomeration [238]. In this process, precursor compounds are dissolved and subsequently recrystallized in a sealed autoclave with an aqueous medium under high temperature and pressure, with water serving as the reaction medium. The physicochemical properties of the resulting nanostructures are strongly governed by synthesis parameters, such as reaction temperature, pressure, precursor concentration, pH, and reaction time. Precise control of these parameters promotes rapid nucleation and regulated crystal growth, enabling the formation of nanostructured cathode materials with tailored morphology, particle size, and lattice structure. Consequently, the HTM is particularly effective for synthesizing highly crystalline and compositionally uniform metal oxides with controlled microstructural features. A key advantage of the HTM lies in its ability to achieve excellent control over stoichiometry, high phase purity, and desirable crystal architectures while using relatively low-cost raw materials. Compared to conventional solid-state or high-temperature synthesis routes, the HTM offers enhanced control over particle size distribution, crystallinity, and structural homogeneity, which are critical factors for improving electrochemical performance. Despite these advantages, the HTM also exhibits several limitations. The process requires specialized high-pressure equipment and prolonged reaction times at elevated temperatures, leading to increased energy consumption and operational complexity. Furthermore, its industrial-scale implementation remains complex due to challenges associated with reactor design, safety requirements, and process scale-up. Thus, although the HTM is highly effective for producing advanced nanostructured cathode materials with superior electrochemical properties, its large-scale industrial application remains limited by economic considerations and constraints related to scaling up.

### 4.4. Low-Temperature Wet-Chemical Route

Low-temperature wet-chemical synthesis methods, commonly referred to as “chimie douce”, rely on alcoholic or aqueous precursor solutions to produce cathode materials characterized by high phase purity, controlled stoichiometry, and enhanced homogeneity. Unlike conventional solid-state reactions, these approaches promote mixing at the molecular level, resulting in improved compositional uniformity, reduced particle size, lower processing temperatures, and superior crystallinity.

Due to these advantages, wet routes are widely used to produce lithium-based cathode materials. The most commonly employed techniques include the following:

- *Sol–Gel Process (SGP)* [239,240,241]: A versatile solution-based method that enables precise control over composition, particle morphology, and microstructure through gel formation followed by thermal treatment.
- *Co-Precipitation Method (CPM)* [242,243]: A scalable technique in which metal ions are simultaneously precipitated from solution to form homogeneous precursor particles that are subsequently converted into cathode oxides.
- *Combustion Method (CM)* [244]: A rapid synthesis approach based on highly exothermic redox reactions between metal nitrates and organic fuels, producing fine powders with high surface area and good chemical homogeneity.

**Figure 24:**
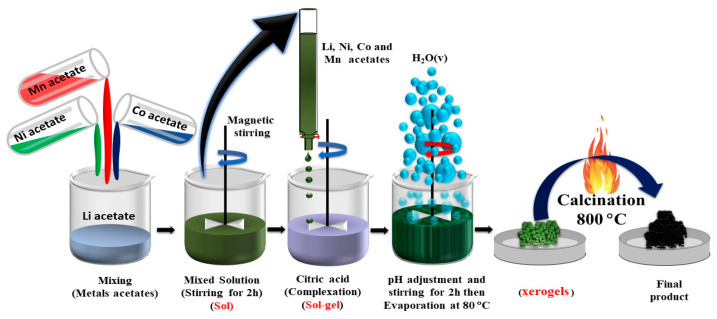
Schematic diagram for the synthesis of cathode materials by the sol–gel method. Reproduced from [245]. Copyright 2025 distributed under the terms and conditions of the Creative Commons Attribution (CC-BY) license.

Given their advantages, wet-chemical synthesis methods are considered effective strategies for tailoring the morphology and electrochemical performance of advanced cathode materials. Among these approaches, the sol–gel method is one of the most widely employed and effective techniques for synthesizing cathode materials, owing to its ability to produce high-purity phases with controlled stoichiometry and morphology. In this process, stoichiometric amounts of precursor materials are initially dissolved in distilled water to form a homogeneous solution. The precursor solutions are then gradually introduced into an aqueous solution—kept under continuous stirring—containing a suitable complexing agent in the desired ratio. Prolonged stirring, typically lasting at least three hours, ensures uniform mixing and promotes complexation between the metal ions and the chelating agent. Following gelation, the solution undergoes slow evaporation, heating, and drying, leading to the formation of a transparent gel. This gel is subsequently transformed into a xerogel, which serves as the precursor for further heat treatment. The xerogel is then calcined to decompose organic constituents and convert metal carboxylates into their corresponding oxides. After cooling to room temperature, the resulting powders are subjected to a second calcination step under controlled conditions, either in air or under vacuum, to obtain the desired crystalline phase and enhance structural stability. This multi-step synthesis process yields cathode materials with uniform particle size, high crystallinity, and excellent phase purity. A schematic illustration of the cathode growth mechanism through the sol–gel synthesis process is presented in Figure 24 [245].

The sol–gel process (SGP) enables precise control over the homogeneous mixing of precursor components, ensuring uniform distribution and promoting the formation and growth of crystalline phases with a narrow particle size distribution. Due to its relatively low reaction temperature, the SGP is generally considered a cost-effective synthesis route compared to other methods operating under similar conditions [239]. Moreover, it is widely recognized as one of the most time- and cost-efficient approaches for producing high-purity cathode materials. Despite these advantages, the SGP also presents several significant limitations. The incorporation of organic chelating agents during synthesis leads to the release of substantial volumes of gaseous by-products during calcination, complicating processing and raising heightened environmental and safety concerns. Moreover, these factors hinder the scalability of the process for industrial applications. Consequently, although the SGP is highly effective for the laboratory-scale preparation of high-purity cathode materials, its large-scale industrial implementation remains challenging.

An innovative variation of the sol–gel process involves the use of bioactive reducing agents derived from natural sources such as green tea, aloe vera, or fruit peels (e.g., orange, tangerine). This environmentally friendly strategy employs plant-based extracts as natural chelating agents, providing a sustainable alternative to conventional organic reagents. The process generally comprises two main stages:

- *Preparation of the bio-reducing agent*: For instance, orange peel extract can be prepared by boiling small pieces of thoroughly washed discarded peels in distilled water at 100 °C for 10 min. The resulting mixture is then filtered to obtain a clear extract suitable for further use [241].
- *Integration into the sol–gel process*: The obtained bio-extract is subsequently introduced into the sol–gel synthesis as a chelating and complexing agent. It promotes metal-ion coordination, enhances precursor homogeneity, and facilitates the formation of uniform gels, thereby improving the sustainability of the synthesis route.

**Figure 25:**
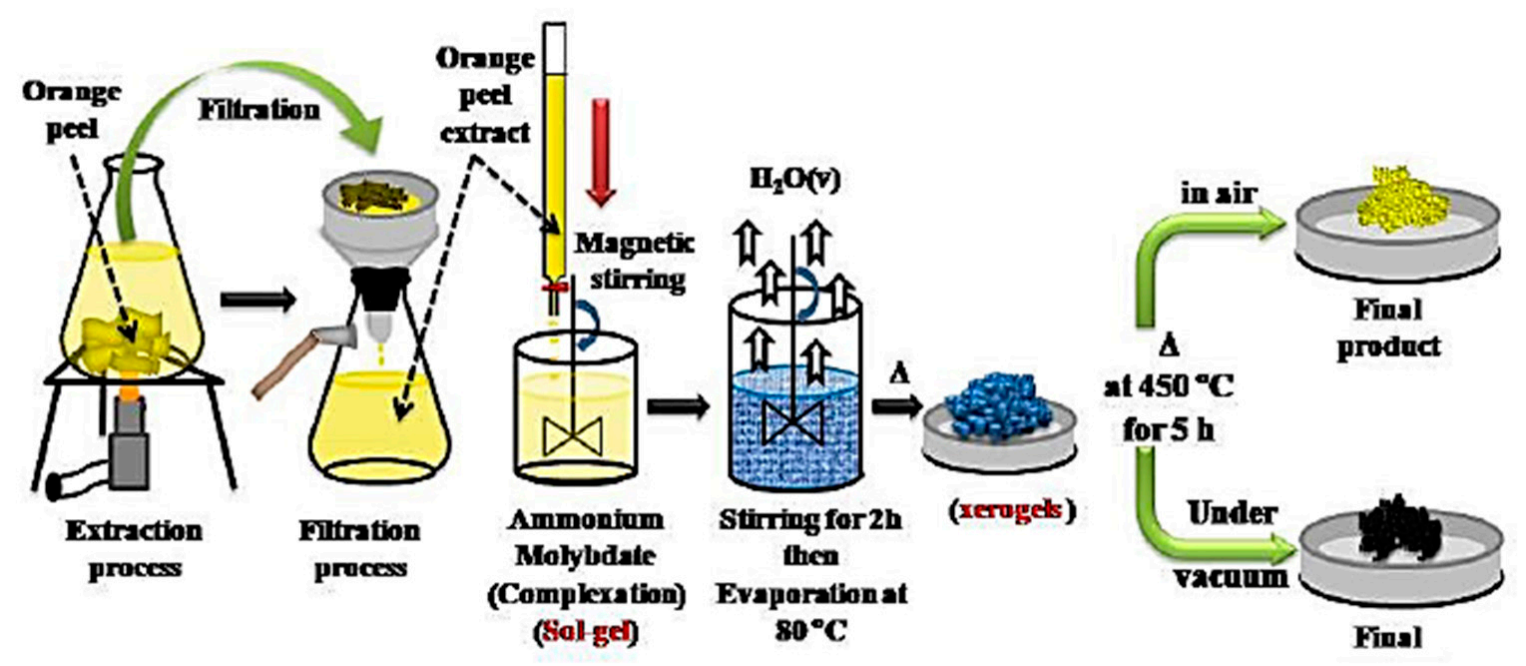
Schematic diagram for the synthesis of cathode materials by the bioactive reducing agent-assisted sol–gel method. Reproduced from [241]. Copyright 2021 under the terms and conditions of the Creative Commons Attribution (CC-BY) license.

This bio-assisted sol–gel approach not only reduces reliance on synthetic organic chelating agents, but also offers a more eco-friendly, cost-effective, and potentially scalable route for the synthesis of cathode materials. Figure 25 schematically illustrates the cathode formation and growth process using orange peel extract within the sol–gel method [241].

### 4.5. Co-Precipitation Methods (CPMs)

**Figure 26:**
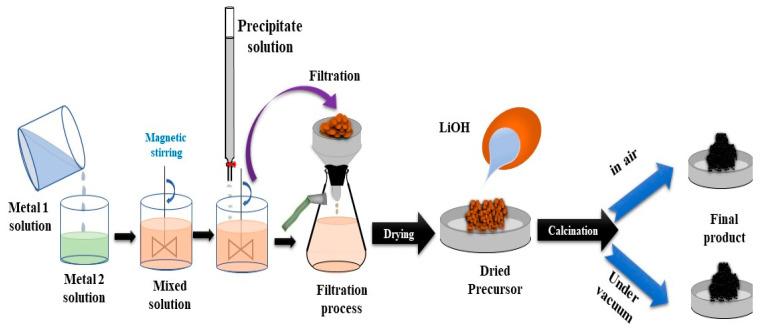
Schematic diagram for the synthesis of cathode materials by the co-precipitation method.

The co-precipitation method (CPM) is widely recognized as an effective synthesis route for obtaining highly crystalline cathode materials, thanks to the control of nucleation and growth kinetics in a homogeneous solution. This method enables the formation of fine, uniformly distributed particles with high specific surface area, which facilitates lithium-ion diffusion and enhances electrochemical performance. Other advantages of the CPM include reduced synthesis time, low energy consumption, and high yield [242]. In a typical CPM, stoichiometric amounts of precursor salts are dissolved in distilled water to obtain a homogeneous, saturated solution. Subsequently, a precipitating agent is added dropwise under continuous stirring, causing the precursor compounds to precipitate. The obtained precipitate is then separated and dried through filtration or evaporation [98]. To synthesize the final cathode material, the precursor is mixed with a stoichiometric amount of LiOH and calcined at an appropriate temperature under a controlled atmosphere (air or vacuum), with intermittent grinding to ensure compositional and structural uniformity. Figure 26 schematically illustrates the cathode growth mechanism during the co-precipitation process. An advanced variant of the CPM involves the hydrogen peroxide-assisted co-precipitation, where H_2_O_2_ acts as both an oxidizing agent and a dispersing agent. This approach improves precursor dispersion and promotes more effective oxidation during synthesis, resulting in enhanced electrochemical performance. For instance, NCM materials prepared via the H_2_O_2_-assisted CPM exhibited higher specific capacity and better cycling stability compared to those synthesized using conventional co-precipitation [243].

Although the co-precipitation method offers several advantages over the sol–gel process—notably precise control of stoichiometry, simplicity in implementation, ease of adjusting synthesis parameters, and relatively short synthesis time—it also presents certain limitations. A major challenge lies in the formation of zones with high precipitant concentrations during the reaction, which can promote particle agglomeration and compositional heterogeneity in the final product. These phenomena can impair the structural uniformity of the synthesized cathode material and, ultimately, compromise its electrochemical performance.

### 4.6. Combustion Method (CM)

The combustion method (CM) is a low-temperature synthesis route for producing cathode materials that eliminates the need for additional calcination steps. In this process, organic fuels such as glycine, urea, or citric acid undergo self-ignition upon heating, thereby generating the thermal energy required to form the target crystal structure. This method is particularly attractive due to its simplicity, low equipment requirements, ability to effectively limit particle agglomeration, and ability to promote the homogeneous incorporation of dopant into the final material.

Requiring a single annealing step, the CM can be applied to both liquid and solid precursors, thereby enabling the synthesis of nanoscale cathode materials [244]. Typically, metal salt precursors are mixed with a suitable fuel to form a xerogel at relatively low temperatures. Upon heating, the xerogel undergoes a highly exothermic combustion reaction, yielding precursor powders composed of ultrafine particles. These powders are subsequently calcined at high temperatures to produce the final crystalline cathode material. Due to its simplicity, low cost, and effective control over particle size and morphology, the CM has attracted considerable interest for cathode synthesis. However, the technique also presents notable drawbacks. In particular, its strong dependence on combustion behavior makes process control challenging and limits reproducibility during scale-up, thereby restricting its suitability for large-scale industrial production.

To facilitate comparison, the main synthesis methods discussed in this section are summarized comparatively in Table 2, highlighting their implementation conditions, particle size control, crystallinity, scalability to an industrial level, and cost, as well as their associated advantages and limitations.

## 5. Structural Optimization

Despite the remarkable advantages of cathode materials in electrochemical cells, several inherent challenges continue to limit their long-term performance. These include low electronic conductivity, irreversible structural degradation, oxygen evolution during charge–discharge cycling, capacity fading at high current densities, thermal and structural instability, and associated safety risks. To overcome these limitations, surface modification has been widely explored as an effective strategy for enhancing cathode performance by minimizing undesirable side reactions between the electrode and the electrolyte. In addition to lattice doping, surface engineering has emerged as one of the most effective approaches for improving cycle structural stability and extending cycle life. Among the various techniques employed, the deposition of thin protective coatings on cathode particles is particularly attractive. These coatings typically consist of metal oxides, metal fluoride, metal polyanionic compounds, or metallic layers [245]. Acting as physical and chemical barriers, they suppress electrolyte decomposition, stabilize the electrode–electrolyte interface, reduce transition-metal dissolution, and mitigate structural degradation during repeated cycling.

**Figure 27:**
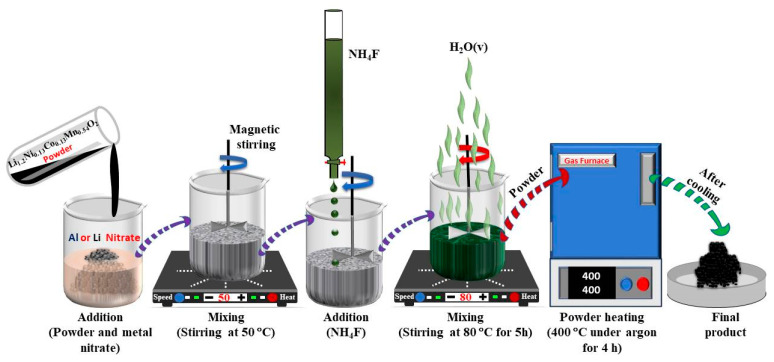
Schematic representation of the AlF_3_/LiF coating process of Li-rich layered cathode nanoparticles. Reproduced from [246]. Copyright 2025 under the terms and conditions of the Creative Commons Attribution (CC-BY) license.

Although coating procedures generally follow similar principles, specific synthesis routes and deposition conditions vary depending on the nature of the coating material and the selected fabrication method. Among the most extensively studied surface coatings, aluminum fluoride (AlF_3_) and lithium fluoride (LiF) have demonstrated significant effectiveness in enhancing interfacial stability and suppressing undesirable parasitic reactions at the cathode–electrolyte interface. As schematically illustrated in Figure 27, the AlF_3_/LiF coating process provides a robust surface-engineering strategy that mitigates degradation mechanisms, improves cycling stability, and prolongs the operational lifetime of lithium-ion batteries [246].

### 5.1. Characterization Techniques

Thermal analysis methods, including thermogravimetric analysis (TG), differential thermal analysis (DTA), and differential scanning calorimetry (DSC), are widely used to assess the thermal behavior and stability of both precursor and synthesized powders. These techniques provide valuable information on mass-loss processes, thermal decomposition, phase transformations, and crystallization phenomena. The resulting data enable the determination of suitable heat-treatment conditions, identification of exothermic and endothermic reactions during calcination, and estimation of crystallization temperatures. Such information is crucial for optimizing synthesis parameters and ensuring the structural stability and phase purity of cathode materials.

**Figure 28:**
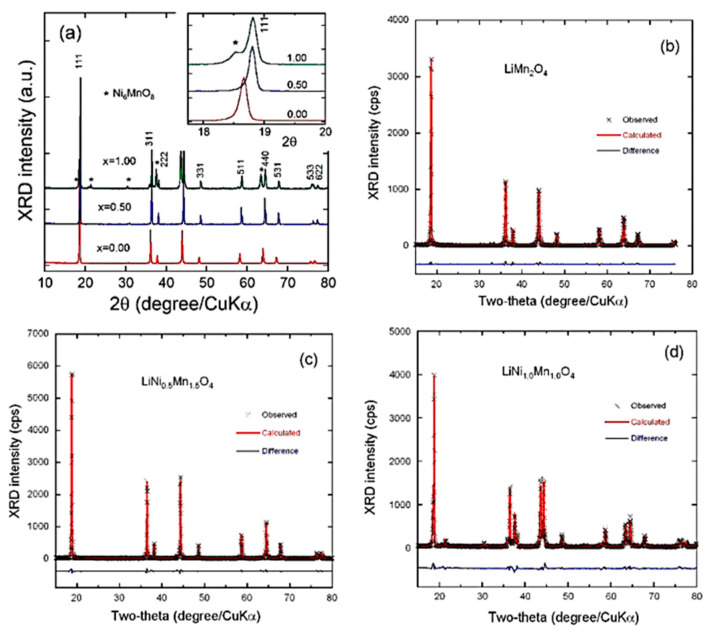
(**a**) XRD patterns of the as-prepared LiNi_y_Mn_2−y_O_4_ (*y* = 0.0, 0.5 and 1.0) spinel samples. The insert shows the reflections at ca. 2*θ* = 18.8°. Stars (*) indicate reflections of Ni_6_MnO_8_ impurity. (**b**) Rietveld refinement of Ni-rich LiNiMnO_4_. (**c**) Rietveld refinement of the 4-volt spinel LiMn_2_O_4_. (**d**) Rietveld refinement of the 5-volt LiNi_0.5_Mn_1.5_O_4_. Reproduced from [247]. Copyright 2021 under the terms and conditions of the Creative Commons Attribution (CC-BY) license.

X-ray diffraction (XRD) remains the most powerful technique for the structural characterization of cathode materials, enabling precise assessment of phase purity, crystallinity, and structural evolution during synthesis. Analysis of diffraction patterns provides valuable information on phase transitions and lattice distortions. To obtain detailed structural parameters, XRD data are commonly analyzed using the Rietveld method, which minimizes the differences between calculated and experimental diffraction profiles. This approach enables the determination of atomic coordinates, displacement parameters, site occupancies, and phase fractions, providing a comprehensive description of the crystal structure. For instance, XRD was used to examine the influence of increasing Ni content in the crystal structure of LiNi_y_Mn_2−y_O_4_ spinels with *y* = 1, synthesized by an EDTA-assisted sol–gel route (Figure 28) [247]. The Ni-rich sample was found to crystallize as a biphasic material consisting of a non-stoichiometric LiNiMnO_4−δ_ spinel phase (space group *Fd*$\bar{3}$*m*) combined with a secondary Ni_6_MnO_8_ phase (space group *Fm*3*m*). Rietveld refinements indicated a composition (1 − *z*) LiNiMnO_4_·*z*Ni_6_MnO_8_ with *z* = 37.2. Furthermore, the results showed that only 15.5% of the Ni^2+^ ions were incorporated into the *Fd*$\bar{3}$*m* spinel structure when 0.5Ni was introduced into the LiNi_0.5_Mn_1.5_O_4_ lattice, highlighting the limited solubility of excess nickel in the spinel framework.

**Figure 29:**
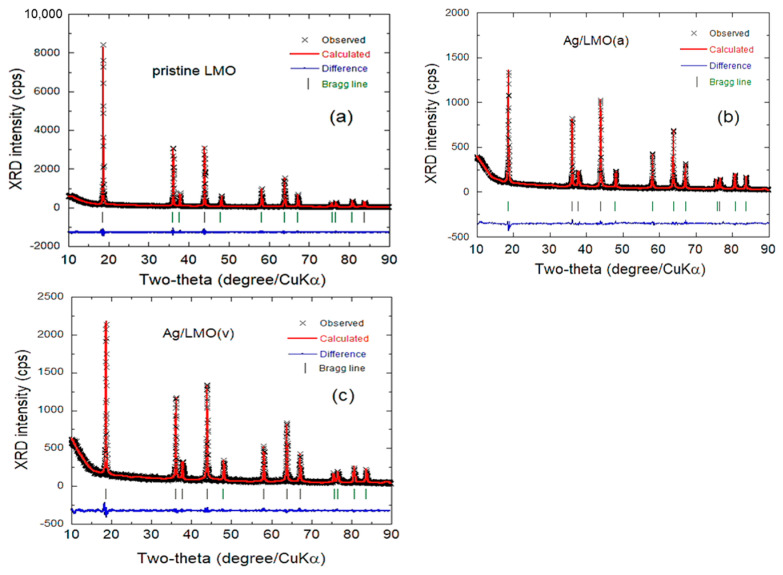
Rietveld refinements of XRD patterns of (**a**) as-prepared pristine LMO, (**b**) Ag/LiMn_2_O_4_ treated in air, and (**c**) Ag/LMO calcined in vacuum. Reproduced from [248]. Copyright 2020 under the Creative Commons Attribution (CC-BY) license.

XRD has been widely used to investigate the influence of calcination conditions on Ag-coated LiMn_2_O_4_, particularly for determining the oxidation state and phase distribution of silver [248]. Rietveld refinement revealed that calcination in air led to the formation of a predominantly insulating AgO layer (3.2%) with only trace amounts of metallic Ag (0.1%). In contrast, vacuum calcination produced mainly metallic Ag nanoparticles (2.6%), present as nanospheres, together with a smaller fraction of AgO (0.8%) coating the LiMn_2_O_4_ particles. These results demonstrate the strong impact of processing atmosphere on the surface chemistry of modified cathode materials and highlight the effectiveness of XRD in characterizing associated structural and compositional changes (Figure 29).

**Figure 30:**
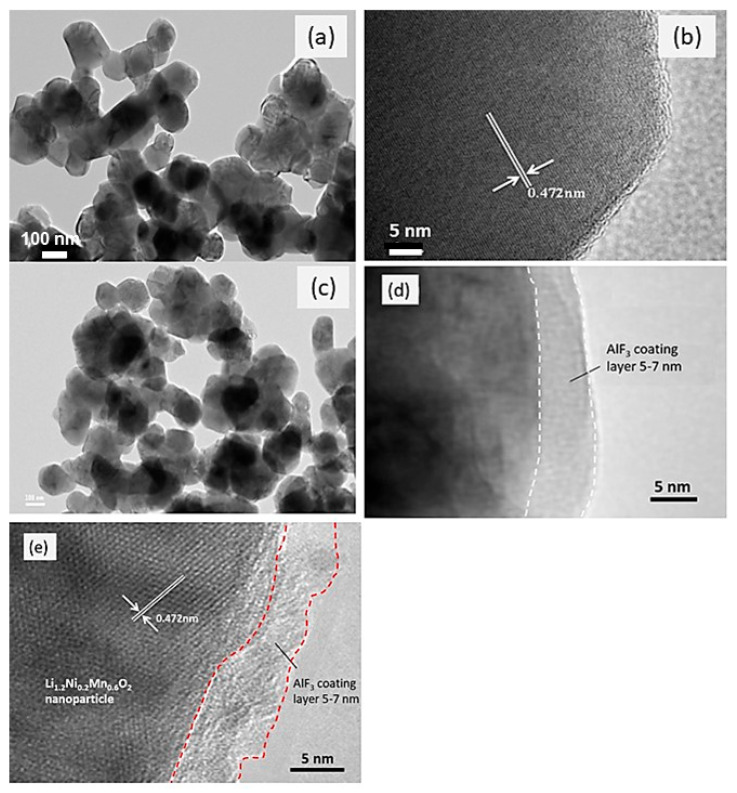
TEM images of (**a**) pristine Li_1.2_Ni_0.2_Mn_0.6_O_2_ and (**b**) AlF_3_-coated Li_1.2_Ni_0.2_Mn_0.6_O_2_. HRTEM images of (**c**) pristine Li_1.2_Ni_0.2_Mn_0.6_O_2_ and (**d**) AlF_3_-coated Li_1.2_Ni_0.2_Mn_0.6_O_2_. Image (**e**) shows the morphology of the AlF_3_ coating [249]. Copyright 2022 under the Creative Commons Attribution (CC-BY) license.

The morphology of cathode particles is primarily determined by the synthesis method, which significantly influences their electrochemical performance. Scanning electron microscopy (SEM) is widely used to examine surface morphology and compositional features by scanning the sample with a focused electron beam at the micro- and nanoscale. SEM provides detailed information on particle size, distribution, and surface texture. Transmission electron microscopy (TEM) and high-resolution TEM (HRTEM) offer deeper insights into material structure through electron–matter interactions, enabling the characterization of morphology, crystallinity, strain, and defects. TEM reveals structural features such as dislocations, grain boundaries, and layer growth, while HRTEM allows for the direct visualization of crystal lattice fringes and defects at the atomic scale. Furthermore, the successful incorporation of dopants or surface coatings can be verified by energy-dispersive X-ray spectroscopy (EDX), a technique commonly coupled with TEM/SEM analyses. As an example, Figure 30 presents TEM images of pristine and AlF_3_-coated Li-rich cathode materials (Li_1.2_Ni_0.2_Mn_0.6_O_2_).

**Figure 31:**
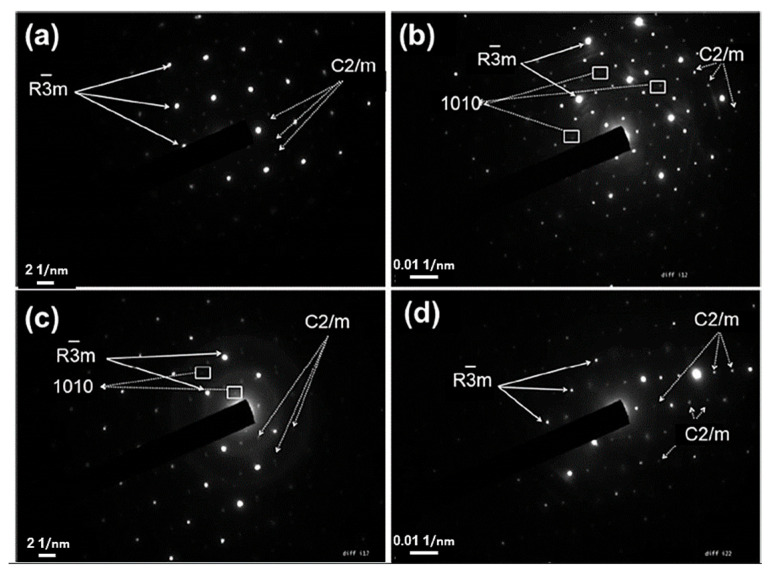
SAED patterns of Li_1.2_Ni_0.13_Mn_0.54_Co_0.13_O_2_ powders synthesized with and without chelating agent-assisted sol–gel method: (**a**) without chelating, (**b**) CA/EDTA, (**c**) CA and (**d**) EDTA. Reproduced from [70]. Copyright 2020 Springer.

SEM and TEM are widely employed not only to differentiate samples based on their morphology and grain size but also to evaluate surface characteristics of coated materials, including coating thickness, uniformity, and homogeneity. For example, TEM and HRTEM analyses performed on an AlF_3_-coated layered cathode with the composition Li_1.2_Ni_0.2_Mn_0.6_O_2_ (synthesized via the hydrothermal route) demonstrated that the coating process altered neither the particle size nor the morphology. A uniform AlF_3_ layer, approximately 5–7 nm thick, was observed, indicating that the coating had no detrimental structural impact and preserved the original particle architecture [249]. Furthermore, selected area electron diffraction (SAED) provides valuable structural information by enabling the identification of multiple phases and crystallographic distortions through characteristic diffraction patterns. By way of illustration, SAED analyses conducted on Li-rich layered powders (Li_1.2_Ni_0.13_Mn_0.54_Co_0.13_O_2_)—synthesized via the sol–gel method using different chelating agents (citric acid, EDTA, and CA/EDTA)—revealed disruptions to the rhombohedral symmetry in samples prepared with citric acid and EDTA (Figure 31). These distortions were attributed to the presence of stacking faults and dislocations within the crystal lattice [70].

Raman scattering (RS) and Fourier transform infrared (FTIR) spectroscopy are highly sensitive probes of the short-range oxygen coordination environment surrounding cations in oxide lattices. Their sensitivity to local structure makes them particularly valuable for phase identification when multiple local environments coexist. The frequencies and relative intensities of vibrational bands are primarily governed by the cation coordination geometry and oxidation state, whereas grain size and long-range structural order have a comparatively minor influence. Moreover, vibrational spectroscopies are well suited for the characterization of amorphous materials, for which conventional X-ray diffraction (XRD) provides limited structural information [250].

RS probes the local oxygen environment via molecular vibrational modes, thereby providing information on structural distortions, phase transitions, and local bonding configurations in cathode materials. FTIR spectroscopy yields complementary data on metal–oxygen interactions within the cathode lattice; it is often combined with other characterization techniques to evaluate lattice anisotropy and the degree of covalency in interlayer regions. This information is essential for understanding and improving the structural stability of framework materials, particularly those deviating from cubic symmetry.

**Figure 32:**
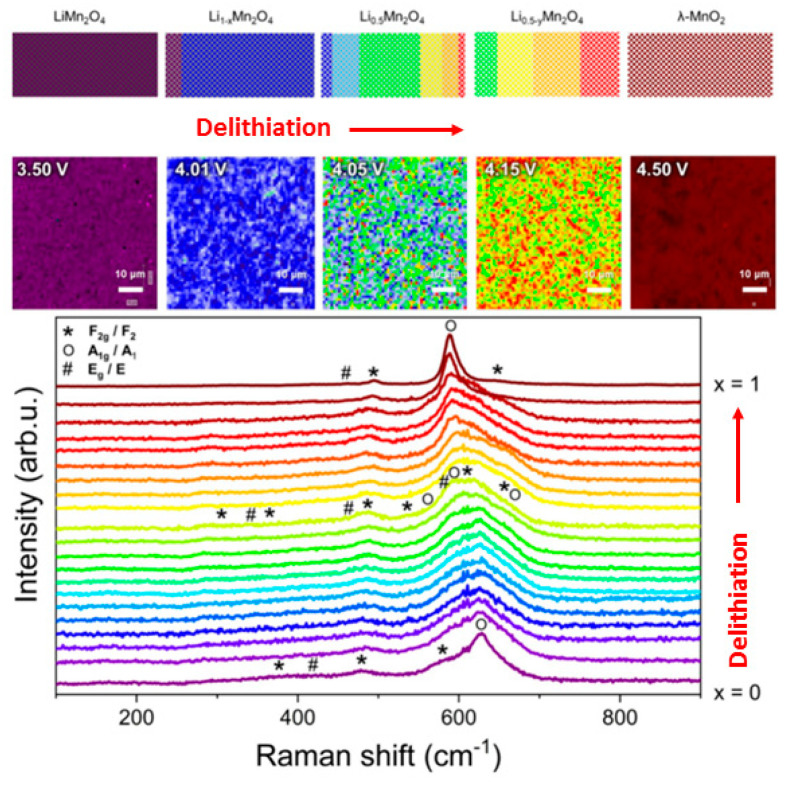
Ex situ Raman mapping on LMO electrodes at different states of charge. Raman maps and corresponding spectra showing clearly that Li deintercalation is a three-phase process in the Li_1−x_Mn_2_O_4_ structure (colors on the maps correspond to the colors of spectra below them). The schematic pictures of the delithiation process are also presented at the top. Reproduced from [251]. Copyright 2024 under the terms of the CC-BY 4.0 license.

For example, ex situ Raman mapping was employed to investigate the structural changes in LMO during delithiation [251]. Raman maps clearly reveal stable structural states at each electrochemical potential (Figure 32). In the fully discharged state, the majority phase corresponds to LiMn_2_O_4_ (purple zone), while a minority population of Li-rich Li_1+z_Mn_2_O_4_ crystallites is identified by a shift in the high-frequency Raman band from 627 to 635 cm^−1^. This shift indicates a shortening of the Mn−O bond and a distortion of the MnO_6_ octahedra, resulting from the occupation of 16*d* octahedral sites by Li^+^ ions within the spinel framework. Another study focused on the atomic displacements associated with Raman- and IR-active vibrational modes in LiMn_2_O_4_ spinels synthesized via the sol–gel method using various chelating agents (citric acid and EDTA) [237].

Although the Raman and FTIR spectra of the two samples were largely similar, slight peak shifts observed for the EDTA-assisted sample were attributed to variations in lattice parameters, which contribute to the stabilization of non-cubic structural motifs. Analyses also revealed local lattice distortions associated with Jahn–Teller active Mn^3+^ ions, manifested by an increase in unit-cell volume and peak broadening. Furthermore, the breaking of translational symmetry induced by Mn^3+^ ions activated additional vibrational modes, observable through spectral decomposition. These studies demonstrate the effectiveness of Raman and FTIR spectroscopy in identifying subtle lattice distortions, structural instabilities, and local symmetry changes in LiMn_2_O_4_-type cathode materials.

X-ray photoelectron spectroscopy (XPS) is a versatile surface analysis technique that enables both qualitative and quantitative characterization of materials. Qualitatively, it provides information on elemental composition, empirical formulas, and the chemical and electronic states of the elements present at the surface. Quantitatively, XPS peak intensities can be deconvoluted to estimate the relative concentrations of various chemical species, thereby allowing the determination of average oxidation states. In the field of LIBs, XPS proves particularly valuable for elucidating the intercalation mechanisms of cathode materials. By monitoring changes in the oxidation states of 3d transition-metal cations and oxide species as a function of lithium content within the host structure, XPS provides essential insights into the structural and electronic destabilization processes occurring during charge–discharge cycles. For instance, XPS analysis of lithium manganese oxide reveals peaks at 642.1 eV and 643.6 eV, assigned to Mn^3+^ and Mn^4+^ species, respectively [239]. These values align with those reported for Mn^3+^ in Mn_2_O_3_ and Mn^4+^ in MnO_2_ [252]. Deconvolution of Mn 2p spectra enables quantification of the relative proportions of Mn^3+^ and Mn^4+^, from which average valence states can be derived. In the case of LiMn_2_O_4_ synthesized using EDTA and citric acid, the calculated average Mn valence states were 3.505 and 3.56, respectively, confirming the expected mixed-valence character of manganese in the spinel structures.

**Figure 33:**
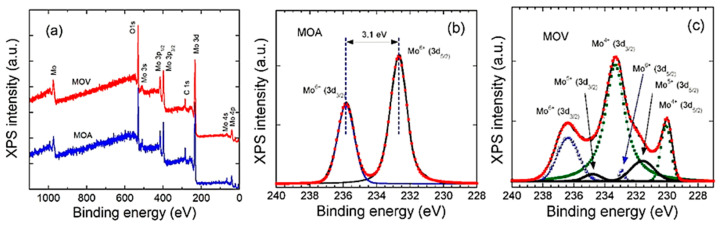
(**a**) XPS survey spectra of the MOA and MOV samples. High-resolution XPS spectra of (**b**) Mo 3d in MOA and (**c**) Mo 3d in MOV. Reproduced from [241]. Copyright 2021 under the terms and conditions of the Creative Commons Attribution (CC-BY) license.

The XPS technique is also powerful for probing the influence of calcination conditions on surface chemistry. For example, Figure 33 compares MoO_3_ (MOA) calcined in air with MoO_2_ (MOV) calcined under vacuum at 450 °C [241]. XPS spectra reveal the presence of minor Mo-suboxides (MoO_2_, Mo_4_O_11_, Mo_8_O_23_, Mo_9_O_26_), indicating mixed valence states at the MOV surface. These results highlight the sensitivity of XPS to subtle changes in oxidation state and surface composition—parameters that are critical for understanding cathode stability and electrochemical performance.

### 5.2. Electrochemical Properties

**Figure 34:**
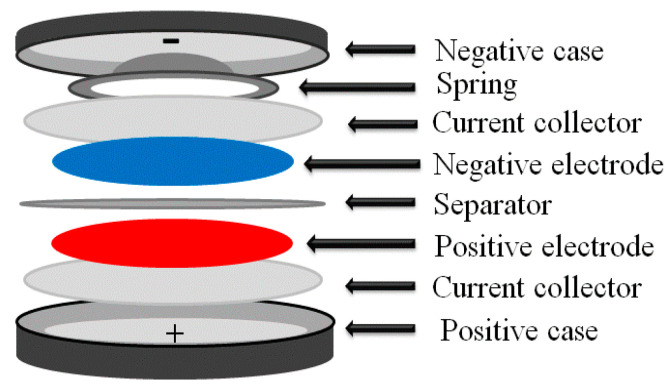
Schematic representation of the coin cell assembly.

Evaluating electrochemical properties is a crucial step in determining the suitability of cathode materials for LIBs, as these properties directly determine their performance and practical applicability. The most commonly employed techniques are galvanostatic charge–discharge (GCD) cycling and cyclic voltammetry (CV). These methods provide data on the evolution of the electrode potential relative to lithium content during charge and discharge processes, thereby enabling the study of redox reactions, electrochemical reversibility, and structural stability. Electrochemical measurements are typically conducted under various operating conditions, including different current rates, temperatures, and voltage ranges, to evaluate the material’s behavior under real-world usage conditions. Furthermore, electrochemical impedance spectroscopy (EIS) is frequently performed on both pristine and cycled electrodes to investigate the kinetic processes governing battery operation. Via analysis of Nyquist diagrams (-Im(*Z*) versus Re(*Z*) plots), EIS yields valuable insights into charge-transfer resistance, lithium-ion diffusion, and electrode–electrolyte interfacial stability, thereby contributing to a deeper understanding of the factors that determine cathode performance. Conducting these electrochemical investigations requires the assembly of an experimental battery in either half-cell configuration. The synthesized cathode material is generally deposited onto a metallic current collector (copper), while a metallic lithium metal sheet serves as the counter and reference electrode. Figure 34 illustrates the assembly diagram of a coin cell prepared in an argon-filled glove box, highlighting the configuration commonly used for the electrochemical characterization of cathode materials.

**Figure 35:**
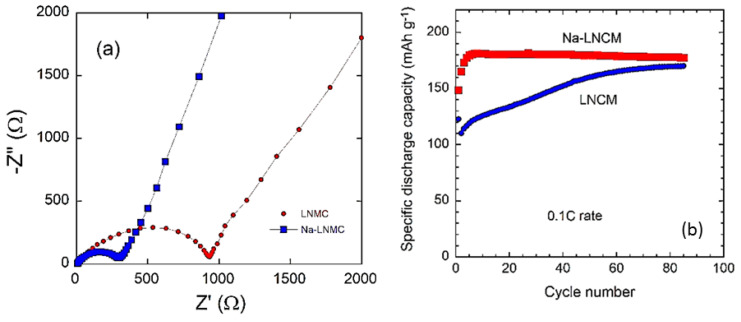
(**a**) Nyquist plots and (**b**) Comparison of cycling behaviors of the pristine Li_1.2_Ni_0.13_Co_0.13_Mn_0.54_O_2_ (LNMC) and Na-doped Na-Li_1.2_Ni_0.13_Co_0.13_Mn_0.54_O_2_ (Na-LNMC) electrodes. Reproduced from [253]. Copyright 2022 under the terms and conditions of the Creative Commons Attribution (CC-BY) license.

It has been demonstrated that incorporating a small amount of sodium into lithium-rich cathode materials significantly improves their electrochemical performance [253]. As shown in Figure 35a, the introduction of 3 mol.% Na reduces the charge-transfer impedance by more than three orders of magnitude while simultaneously increasing the lithium-ion diffusion coefficient by a similar margin [253]. These enhancements in electronic conductivity and ion transport kinetics directly contribute to improved electrochemical reversibility and lead to better capacity retention. As illustrated in Figure 35b, the discharge capacity of the Na-doped Li_1.2_Ni_0.13_Co_0.13_Mn_0.54_O_2_ electrode increases progressively over successive cycles. This gradual activation behavior indicates an increase in the amount of electrochemically active lithium at the particle surface during repeated charge–discharge cycles. These results suggest that Na doping not only facilitates lithium-ion transport, but also helps stabilize the electrode–electrolyte interface, thereby promoting sustained electrochemical activity and enhancing the long-term cycling performance of lithium-rich layered cathode materials.

## 6. Insights and Future Prospects

### 6.1. Insights

Nanostructuring of cathode materials has emerged as an effective strategy to address several intrinsic limitations encountered in conventional lithium-ion batteries, including sluggish Li^+^ diffusion, low electronic conductivity, and structural degradation during cycling. Through nanoscale engineering, significant improvements have been achieved in charge/discharge capacity, reversible capacity utilization, rate capability, and cyclic stability across various cathode families, such as layered oxides (e.g., LCO, NMC, and NCA), spinel oxides (LMO), and polyanionic compounds (LFP, LMP). The main insights drawn from recent developments are summarized as follows:

- *Shortened lithium-ion diffusion pathways:* Reducing particle size to the nanoscale significantly decreases Li^+^ ion diffusion lengths, while increasing the electrode–electrolyte contact area. Consequently, nanostructured cathodes can deliver superior rate performance, even for materials traditionally limited by slow ion transport kinetics, notably LFP.
- *Improved interfacial stability through surface engineering*: Surface coatings, including carbon, metal oxides, and phosphate layers as well as advanced interface engineering approaches, can effectively mitigate interfacial side reactions, limit transition-metal dissolution, and stabilize the cathode/electrolyte interface, particularly under high-voltage operating conditions.
- *Enhanced structural integrity during cycling:* Nanosized particles can better accommodate the mechanical strain associated with repeated lithiation and delithiation processes. Their ability to tolerate volume changes reduces microcrack formation and structural degradation, thereby minimizing capacity fading. This effect is particularly important for high-nickel layered oxides and spinel cathodes subjected to aggressive cycling conditions.
- *Higher electronic conductivity through carbon integration*: The incorporation of conductive carbon materials, such as carbon coatings, carbon nanotubes (CNTs), and graphene networks, creates efficient electron-transport pathways. These conductive frameworks improve overall electronic conductivity while limiting the amount of inactive conductive additives required in the electrode.
- *Trade-offs associated with nanostructuring:* Despite their numerous advantages, nanostructured cathodes often exhibit increased surface reactivity, accelerated parasitic reactions, lower tap density, and higher manufacturing costs. These drawbacks highlight the importance of optimizing particle size and morphology rather than pursuing indiscriminate size reduction.

Overall, the successful implementation of nanostructured cathodes requires a delicate balance between enhancing electrochemical performance and addressing practical considerations such as stability, energy density, scalability, and cost.

The primary advantage of the LFP cathode lies in its outstanding thermal stability and very good cycle life, albeit at the expense of energy density. Concerning NMC and NCA cathodes, the “best” performance depends on the priority criterion: energy density, power, cycle life, safety, or cost. A practical comparison of NCA and NMC cathodes used in LIBs has been reported in recent works [254,255,256], and is synthesized in Table 3 below.

In terms of energy density, NCA comes out on top. Currently, this value does not exceed 260 Wh kg^−1^ for NMC811. This is why Tesla has historically used NCA for its long-range EVs. NCA performs better because aluminum stabilizes the structure at high voltage. Conversely, NMC excels in terms of cycle life. Typical ranges are 500 to 1500 cycles for NCA, 1500 to 2000 cycles for NMC. The reason is that Mn improves crystal stability, resistance to cracking, and reduces oxygen release, reducing degradation during cycling. NMC is also safer to manage thermally and benefits from a lower thermal runaway tendency. This is why the market is evolving toward high-nickel NMC chemisties, justifying the research efforts currently dedicated to recycling of Li-ion batteries with NMC cathodes [257].

### 6.2. Future Prospective

The future development of nanostructured cathode materials should focus on bridging the gap between laboratory-scale research and commercially viable battery technologies. Several promising directions can be identified:

- *Advanced Nanostructure Design:* Future research should move beyond conventional nanoparticles toward hierarchical, mesoporous, and oriented nanostructures that can simultaneously provide rapid lithium-ion transport, high tap density, and enhanced mechanical stability.
- *Surface and Interface Engineering:* The development of advanced surface passivation strategies, including ultrathin ion-conductive coatings and artificial cathode -/electrolyte interphases, is crucial for minimizing parasitic side reactions while preserving fast Li^+^ transport kinetics and improving long-term cycling stability.
- *Stabilization of Next-Generation Cathodes:* Nanostructuring is expected to play a crucial role in enabling high-energy-density cathode materials, notably high-nickel layered oxides, lithium-rich oxides, and cobalt-free compounds. These materials often suffer from structural degradation and interfacial instability, challenges that can be mitigated through rational nanoscale engineering.
- *Scalable and Sustainable Manufacturing:* The commercialization of nanostructured cathodes requires cost-effective, scalable, and environmentally sustainable synthesis approaches. Techniques such as green synthesis, spray drying, sol–gel processing, hydrothermal synthesis, and solid-state methods with precise nanoscale control offer significant potential for industrial-scale production.
- *Integration with Advanced Electrolytes:* The co-development of nanostructured cathodes with solid-state electrolytes or high-voltage liquid electrolytes will be essential for achieving safer lithium-ion batteries with wider electrochemical stability windows, higher energy densities, and improved operational reliability.

Overall, the combination of nanostructure engineering, interface optimization, scalable manufacturing, and advanced electrolyte integration is expected to accelerate the commercialization of high-performance lithium-ion batteries for electric vehicles, grid storage, and portable electronics. Table 4 highlights the most familiar cathode materials for lithium-ion batteries. The compact overview is expanded below into a quantitative, family-wide comparison: It extends the same set of cathode materials with theoretical and practical capacity, energy density, capacity retention, thermal-stability onset temperature, relative cost, commercial maturity, dominant failure mechanism, and the most effective modification strategy for each, and additionally incorporates the non-olivine polyanionic and disordered rock-salt families introduced in Section 3.7 and Section 3.1.8.

In summary, the key scientific trends and current research converging on several strategies are as follows:

- Doping to stabilize crystal structures;
- Nanoscale coatings to protect interfaces;
- Single-crystal particles to reduce cracking;
- Defect engineering (vacancies, substitutions) to improve lithium diffusion;
- Controlled activation of anionic redox to increase capacity without sacrificing cycle life.

Outlook and next-generation cathodes are expected to combine the following:

- Specific gravimetric capacity > 250 mAh g^−1^;
- An operating voltage of 4.5–5.0 V;
- Very low or zero cobalt content;
- Compatibility with solid-state electrolytes.

The main challenge remains balancing high energy, stability over thousands of cycles, safety, and controlled costs. To date, nickel-rich cathodes and LFP are the most industrially mature technologies, while lithium-rich materials and anionic redox represent the most promising avenues for achieving a new milestone in energy density (see Refs. [258,259,260]). Table 5 provides a comprehensive comparative summary of cathode materials for lithium-ion batteries.

Note that solid electrolytes introduce new challenges for cathodes in all-solid-state batteries such as interface chemical stability, ion transport, and mechanical stresses. Also, current research focuses on developing ultrathin protective layers, cathode–electrolyte composites, and single-crystal particles that limit interfacial degradation [261,262,263,264,265,266,267,268].

## 7. Conclusions

The development of LIBs is essential for addressing current environmental and technological challenges, particularly due to their widespread use in electric vehicles and advanced electronic devices. This review highlights recent progress in cathode materials, which remain the key determinants of LIB performance. Comprehensive characterization techniques—such as XRD, TEM, SEM, Raman spectroscopy, FTIR, XPS, EIS, and electrochemical testing—have been extensively employed to evaluate the structural, morphological, surface, and electrochemical properties of cathode materials. The combined use of these methods provides valuable insights for identifying and optimizing high-performance cathode candidates.

Nanotechnology has emerged as a critical driver of battery innovation, enabling the development of nanostructured cathode materials with enhanced conductivity, faster ion diffusion, and improved structural stability. This review has summarized the principal nanostructure designs and synthesis strategies, highlighting their advantages and limitations in improving electrochemical performance and mitigating capacity degradation. Overall, the synergy between advanced synthesis techniques, surface engineering, and nanoscale material design offers significant opportunities for the development of next-generation cathode materials. These advances are expected to play a crucial role in meeting the growing energy storage demands of sustainable transportation, portable electronics, and future clean-energy technologies.

Beyond identifying stable compounds, artificial intelligence (AI) has become an effective tool for optimizing the elemental composition of cathode materials. Traditionally, cathode development has progressed through incremental adjustments in transition-metal ratios, followed by repeated synthesis and electrochemical testing. This iterative process becomes prohibitively expensive as the number of compositional variables increases. Machine learning algorithms efficiently explore multidimensional composition spaces by learning correlations between elemental composition and electrochemical performance. For Ni-rich layered oxides, AI models have been employed to identify compositions that simultaneously maximize specific capacity while minimizing structural degradation and thermal instability. Similar approaches have accelerated the search for cobalt-reduced and cobalt-free cathodes, which are increasingly important because of cost, resource availability, and environmental concerns.

## Figures and Tables

**Table 1:** Comparison between LIBs and other types of batteries.

Battery	Specific Energy (Wh kg^−1^)	Energy Density (Wh L^−1^)	Specific Power (W kg^−1^)	Cycle Life (Cycles)
Pb-acid	30–45	60–90	200–300	400–600
Ni-Cd	40–60	80–110	150–350	600–1200
Ni-MH	60–70	130–170	150–300	300
Li-ion	90–130	140–200	250–450	800–1200
Li-polymer	155	220	315	600

**Table 2:** Comparative summary of synthesis methods for nanostructured cathode materials.

Method	Typical Temp.	Reaction/Processing Time	Particle Size Control	Crystallinity	Industrial Scalability	Rel. Cost	Key Advantages	Key Limitations
Solid-state reaction (SSR)	800–1000 °C (calcination)	Long (multi-step grinding/annealing, 10–24 h)	Poor (large, non-uniform particles)	High	Excellent (industry standard)	Low	Simple, scalable, minimal equipment, mass-production compatible	Particle coarsening, broad size distribution, possible impurities
Hydrothermal/solvothermal (HTM)	80–250 °C (+optional post-annealing at 500–750 °C)	Moderate–long (12–48 h, autoclave)	Excellent (fine, homogeneous)	High (with post-annealing)	Limited (autoclave capacity, batch)	Moderate–high	Precise morphology/size control, high phase purity, low agglomeration	High-pressure equipment; high energy input; scale-up difficulty
Sol–gel process (SGP)	Low–moderate + 400–800 °C calcination	Moderate (gelation + drying + calcination, 1–3 days)	Good (narrow distribution)	High	Limited (chelator cost, gas evolution)	Moderate	Excellent compositional homogeneity; tunable stoichiometry	Gaseous by-products; chelator cost; scale-up difficulty
Co-precipitation (CPM)	RT precipitation + 700–900 °C calcination	Short–moderate (fast precipitation; calcination adds hours)	Good (fine, uniform precursor)	High (after calcination)	Good (industrially proven, e.g., NMC precursors)	Low–moderate	Short synthesis time; low energy; high yield; industrially proven	Local supersaturation agglomeration/heterogeneity
Combustion method (CM)	Self-ignition, 300–600 °C (no separate calcination)	Short (single exothermic step + brief post-treatment)	Moderate	Mod–High	Limited (reproducibility at scale)	Low	Simple, low equipment cost; suppresses agglomeration; homogeneous doping	Difficult process control; strong fuel/combustion dependence

**Table 3:** Practical comparison between NCA and NMC cathodes used in lithium-ion batteries.

Property	NCA Cathode	NMC Cathode
Energy density	Very high	High
Typical cell energy	200–300 Wh kg^−1^	150–260 Wh kg^−1^
Power capability	Excellent	Very good
Cycle life	Moderate	Better overall
Thermal stability	Lower	Better
Safety	Requires strong BMS/cooling	More stable
Cost	Higher	Lower
Main use	Tesla, aerospace, EVs	Most EVs, ESS, tools

**Table 4:** Most familiar cathode materials for lithium-ion batteries.

Cathode Material	Crystal Structure	Operating Voltage (V vs. Li/Li^+^)	Capacity (mAh g^−1^)	Key Advantages	Main Challenges	Relevance of Nanostructuring
LiCoO_2_	Layered α-NaFeO_2_	3.9–4.2	~140	High energy density	High cost, thermal instability	Improves rate capability and cycling stability; mitigates surface degradation
LiNi_x_Mn_y_Co_z_O_2_	Layered	3.6–4.3	160–200	Balanced energy density, cost, and safety	Structural degradation at high Ni content	Enhances Li^+^ diffusion and suppresses microcracking
LiNi_0.8_Co_0.15_Al_0.05_O_2_	Layered	3.6–4.3	~200	High specific energy, long cycle life	Thermal instability, moisture sensitivity	Stabilizes structure and improves high-rate performance
LiFePO_4_	Olivine	3.2–3.5	~170	Excellent thermal stability, long cycle life, low cost	Low electronic conductivity, moderate energy density	Essential to overcome poor kinetics via nanosizing and carbon coating
LiMn_2_O_4_	Spinel	~4.0	~140	Low cost; high power capability	Mn dissolution, capacity fading	Reduces strain and improves cycling stability
xLi_2_MnO_3_·(1 − x)LiMO_2_	Layered/composite	4.3–4.8	>250	Very high capacity	Voltage fade; structural instability	Controls phase transformation and oxygen loss
LiMnPO_4_	Olivine	~4.1	~170	High voltage, good safety	Extremely low conductivity	Nanostructuring enables practical rate performance
LiV_3_O_8_, V_2_O_5_	Layered	2.5–4.0	250–300	High capacity, low cost	Poor cycling stability	Enhances structural integrity and kinetics
LiNi_0.5_Mn_1.5_O_4_	Spinel	~4.7	~147	High voltage, Co-free	Electrolyte decomposition at high voltage	Stabilizes electrode–electrolyte interface

**Table 5:** Comprehensive comparative summary of cathode materials for lithium-ion batteries.

Cathode Materials	Energy Density (Wh kg^−1^)	Capacity Retention	Thermal Stability (°C)	Relative Cost	Commercial Maturity	Major Degradation Mechanisms	Most Effective Modification Strategies
LiCoO_2_ (LCO)	230–570	~80%@150 (3.6–4.2 V)	~200	High	Commercial	Phase transition, oxygen evolution, electrolyte oxidation	Doping, surface coatings, particle engineering
LiNiO_2_ (LNO)	700–800	Poor undoped (<70%@100)	190–210	Moderate	Limited	Li/Ni cation mixing, structural instability	Doping, optimized synthesis, coatings
NMC333	600–650	~90% @500–1000	250–300	High–moderate	Highly commercial	Surface reconstruction, microcracking, oxygen release	Gradient design, single crystal, doping, coatings
NMC622/ NMC811	230–570	70–90% @500–1000	190–240	Low–moderate	Fully commercial	H2 → H3 transition; microcracking; O release	Gradient/FCG design; Zr/Ti/Al doping
NCA	230–570	Moderate (500–1500)	210–230	High	Commercial	Thermal instability, surface degradation	Al doping, coatings, single crystal
LLNMC	230–570	Poor–voltage fade	250–280	Moderate	Emerging	Voltage fade, oxygen redox instability	Surface coating, defect engineering, doping
LiMn_2_O_4_ (LMO)	230–570	70–95% @100–500	~250	Low	Commercial	Mn dissolution, Jahn–Teller distortion	Doping, coatings, electrolyte optimization
LiNi_0.5_Mn_1.5_O_4_ (LNM)	230–570	Moderate–good	280–300	Low	Emerging	Electrolyte oxidation, Mn dissolution	Surface modification, electrolyte additives
LiFePO_4_ (LFP)	230–570	>90% @1000–2000+	Excellent (>270)	Low	Highly commercial	Low electronic conductivity	Carbon coating, nanostructuring, conductive additives
LiMn_x_Fe_1−x_PO_4_	230–570	85–98%@500–2500	Excellent	Low	Early commercial	Low conductivity, Mn dissolution	Carbon coating, Mn/Fe optimization
LiMnPO_4_ (LMP)	230–570	Moderate (rate-limited)	Excellent	Low–moder.	Lab scale	Very low conductivity; JT distortion; antisite defects	Tailored carbon coating; Fe/Co co-doping
LiCoPO_4_/ LiNiPO_4_	230–570	Poor (electrolyte instability)	Excellent (framework)	High–moder.	Lab scale only	Electrolyte oxidation > 4.5 V; impurity phases	AlPO_4_/FePO_4_ coating; high-voltage electrolytes
Other polyanionic	230–570	Good (framework-dependent)	Excellent	Moderate	Emerging	Low conductivity, synthesis complexity	Doping, carbon coating, morphology control
Disordered rock-salt (DRS)	230–570	Poor–moderate (percolation-limited)	Moderate	Moderate	Research stage	Oxygen loss, voltage hysteresis, sluggish Li diffusion	Fluorination, short-range-order engineering, cation disorder control

## Data Availability

No new data were created or analyzed in this study. Data sharing is not applicable to this article.

## Abbreviations

The following abbreviations are used in this manuscript:

ATP	Atom-probe tomography
CA	Citric acid
CG	Concentration gradient
CM	Combustion method
CPM	Co-precipitation method
CV	Cyclic voltammetry
DFT	Density functional theory
DRS	Disordered rock salt
DSC	Differential scanning calorimetry
DTA	Differential thermal analysis
EDTA	Ethylene diamine tetra-acetic acid
EDX	Energy-dispersive X-ray spectroscopy
EIS	Electrochemical impedance spectroscopy
EPMA	Electron probe microanalysis
EV	Electric vehicle
FCG	Full concentration gradient
FTIR	Fourier transform infrared
HRTEM	High-resolution transmission electron microscopy
HTM	Hydrothermal method
LCO	LiCoO_2_
LFP	LiFePO_4_
LIB	Lithium-ion battery
LLMO	Li_1+y_Mn_2−y_O_4−δ_
LMCN	Li_1.2_Ni_0.13_Mn_0.54_Co_0.13_O_2_
LMO	LiMn_2_O_4_
LMP	LiMnPO_4_
LMFP	LiMn_1−y_Fe_y_PO_4_
LNO	LiNiO_2_
LZO	Li_2_ZrO_3_
NCA	LiNi_0.8_Co_0.15_Al_0.05_O_2_
NMC	LiNi_1−x−γ_Mn_x_Co_γ_O_2_
NMC333	LiNi_1/3_Mn_1/3_Co_1/3_O_2_
NMC532	LiNi_0.5_Mn_0.3_Co_0.2_O_2_
NMC622	LiNi_0.6_Mn_0.2_Co_0.2_O_2_
NMC811	LiNi_0.8_Mn_0.1_Co_0.1_O_2_
RIXS	Resonant inelastic X-ray scattering
RS	Raman spectroscopy
SAED	Selected area electron diffraction
SEM	Scanning electron microscopy
SSG	Self-sol–gel
SSR	Solid-state reaction
TEM	Transmission electron microscopy
TG	Thermal gravimetry
XPS	X-ray photoelectron spectroscopy
XRD	X-ray diffractometry
